# Male apoE*3‐Leiden.CETP mice on high‐fat high‐cholesterol diet exhibit a biphasic dyslipidemic response, mimicking the changes in plasma lipids observed through life in men

**DOI:** 10.14814/phy2.13376

**Published:** 2017-10-16

**Authors:** Yared Paalvast, Albert Gerding, Yanan Wang, Vincent W. Bloks, Theo H. van Dijk, Rick Havinga, Ko Willems van Dijk, Patrick C. N. Rensen, Barbara M. Bakker, Jan Albert Kuivenhoven, Albert K. Groen

**Affiliations:** ^1^ Department of Pediatrics University Medical Center Groningen Groningen The Netherlands; ^2^ Department Medicine Division Endocrinology Leiden University Medical Center Leiden The Netherlands; ^3^ Einthoven Laboratory for Experimental Vascular Medicine Leiden University Medical Center Leiden The Netherlands; ^4^ Department of Human Genetics Leiden University Medical Center Leiden The Netherlands; ^5^ Department of Laboratory Medicine University Medical Center Groningen Groningen The Netherlands; ^6^ Department of Vascular Medicine Amsterdam Medical Center Amsterdam The Netherlands

**Keywords:** Biphasic, dyslipidemia, insulin resistance, metabolic syndrome

## Abstract

Physiological adaptations resulting in the development of the metabolic syndrome in man occur over a time span of several decades. This combined with the prohibitive financial cost and ethical concerns to measure key metabolic parameters repeatedly in subjects for the major part of their life span makes that comprehensive longitudinal human data sets are virtually nonexistent. While experimental mice are often used, little is known whether this species is in fact an adequate model to better understand the mechanisms that drive the metabolic syndrome in man. We took up the challenge to study the response of male apoE*3‐Leiden.CETP mice (with a humanized lipid profile) to a high‐fat high‐cholesterol diet for 6 months. Study parameters include body weight, food intake, plasma and liver lipids, hepatic transcriptome, VLDL – triglyceride production and importantly the use of stable isotopes to measure hepatic de novo lipogenesis, gluconeogenesis, and biliary/fecal sterol secretion to assess metabolic fluxes. The key observations include (1) high inter‐individual variation; (2) a largely unaffected hepatic transcriptome at 2, 3, and 6 months; (3) a biphasic response curve of the main metabolic features over time; and (4) maximum insulin resistance preceding dyslipidemia. The biphasic response in plasma triglyceride and total cholesterol appears to mimic that of men in cross‐sectional studies. Combined, these observations suggest that studies such as these can help to delineate the causes of metabolic derangements in patients suffering from metabolic syndrome.

## Introduction

Physiologic adaptations resulting in the development of the metabolic syndrome in man occur over a time span of several decades. For example, central adiposity builds up over a time‐frame of years and decades (Reaven 1988; Lebovitz and Banerji 2005). Furthermore, studies suggest that parameters of the metabolic syndrome such as insulin resistance, muscle mass, plasma triglycerides, and low‐density lipoprotein cholesterol (LDL‐c) are age dependent (Janssen et al. 2000; Murakata et al. 2015). Moreover, the relation between these parameters and age appears nonlinear. A cross‐sectional study in Japan showed biphasic responses for body mass index, serum triglycerides and LDL‐c in men, but not for women (Murakata et al. 2015). Finally, a cross‐sectional study performed in a predominantly Caucasian cohort showed nonlinear relationship between age and skeletal muscle mass (Janssen et al. 2000). It is not clear why these age‐associated changes occur, but decreased physical activity and a decrease in growth‐hormone and gonadal hormones have been proposed as possible explanations (Janssen et al. 2000; Murakata et al. 2015). In contrast, insulin resistance appears to only increase with increasing age (Muller et al. 2014). In any case, time is an important factor, and a longitudinal setup should therefore be preferred above a cross‐sectional setup to elucidate the mechanisms orchestrating the metabolic syndrome.

Although scarce, some longitudinal studies of metabolic syndrome in man have been performed. Scuteri et al. (2009) found that triglycerides in men increased with time, however, decreased in healthy men in the 65–75 years age group, while in women triglycerides increased in all age groups. Furthermore, Meigs et al. (2003) found that increased fasting glucose does not necessarily progress to diabetes when subjects are followed up for over 10 years. Hamer et al. (2015) found that 45% of subjects classified as being “healthy obese” progressed to an unhealthy state (e.g., high triglycerides, impaired glycemic control) compared with 16% of normal‐weight subjects over a period of 8 years. While these longitudinal studies are very valuable, a major limitation is that (more laborious) metabolic flux measurements (i.e., VLDL – triglyceride [TG] production, sterol balance) have not been carried out, and for obvious reasons such studies can only sample a fraction of the human lifespan.

Animal models of metabolic syndrome allow for studying metabolic alterations during development of the metabolic syndrome in a shorter time‐frame (Hariri and Thibault 2010). However, translation is limited by species‐specific characteristics and responses to perturbations designed to induce the metabolic syndrome (Hackam and Redelmeier 2006). For example, the fact that mice have very efficient clearance of triglyceride‐rich lipoproteins limits extrapolation to humans, who generally achieve higher plasma TG concentrations (Van Vlijmen et al. 1998). Similarly, while mice carry most of their plasma cholesterol in high‐density lipoprotein (HDL), humans have a large LDL‐c fraction (Wang et al. 2011). To mitigate these species‐specific differences, we make use of “humanized” apoE*3‐Leiden.CETP (E3L.CETP) mice, in which the apoE*3‐Leiden transgene confers reduced clearance of triglyceride‐rich lipoprotein (i.e., chylomicron‐ and VLDL‐remnants), and the cholesteryl ester transfer protein (CETP) transgene further adds to a cholesterol profile more closely resembling that of humans (Westerterp et al. 2006). Interestingly, in contrast to other mouse models of atherosclerosis, the E3L.CETP mouse has been shown to respond similar to treatment with statins, fibrates, and ezetimibe as humans in terms of changes in plasma cholesterol and atherosclerotic progression (Zadelaar et al. 2007). Moreover, E3L.CETP mice respond similar to humans to antidiabetic drugs as well (van den Hoek et al. 2014). However, whether E3L.CETP mice are a good model to study the development of metabolic syndrome through time is unclear to date.

Here, we study whether male E3L.CETP mice are an adequate model to study the metabolic syndrome in man, by following their response to a high‐fat high‐cholesterol diet (HFCD) for 6 months. We found that both dyslipidemia and parameters of insulin resistance followed a biphasic trajectory. A biphasic trajectory in dyslipidemia is also found in man during aging. These data indicate that male E3L.CETP mice on HFCD may be an adequate model to study the age‐dependent changes of plasma lipids in man.

## Methods

### Animals, diet, and housing

Male E3L.CETP mice (van den Maagdenberg et al. 1993; Wang et al. 2011) were housed individually and fed a synthetic HFCD diet containing 60% fat in energy and 0.25% cholesterol in weight (D12429, Research Diets) in a light (lights on at 7:00 AM–lights off at 7:00 PM) and temperature‐controlled (21°C) facility. Prior to the start of the experimental period, mice were co‐housed with siblings and fed chow ad libitum with unlimited access to drinking water. At least 1 week prior to the start of the experiment animals were housed individually to acclimatize. Experimental procedures were approved by the Ethics Committees for Animal Experiments of the University of Groningen.

### Experimental setup

Mice were fed a HFCD diet at the age of 4 months. Four cohorts of mice were fed HFCD diet ad libitum for 4 (*n* = 20), 9 (*n* = 19), 13 (*n* = 20), and 28 weeks (*n* = 30), respectively. At the end of the dietary intervention, mice in the respective cohorts were distributed evenly in two groups, where one group underwent measuring of VLDL‐TG production and the other group was used for measuring hepatic de novo lipogenesis, for bile cannulation and tissue collection. Animals were distributed among the two groups in the week of termination by placing every consecutive age‐sorted mouse in a different group. An additional cohort (*n* = 16) was used specifically for measuring endogenous glucose production at week 3, 9, 15, and 27 of the dietary intervention. These animals also underwent either VLDL‐TG production measurement or bile cannulation and tissue collection. In all groups, blood samples were obtained by tail bleeding at 4–6 week intervals, to determine plasma TG, plasma total cholesterol (TC), HDL‐c, and glucose. In addition, 24‐h feces were collected from all groups at 4–6 week interval. All flux measurements and blood sample collections started at 1 PM in the fasting condition, with food being removed at 9 AM.

### Drop outs

Of the 115 animals included in the study, in total 10 animals (2 in the 13 week cohort, and 8 in the respective 28 week cohorts) had to be terminated prematurely because of rapid weight loss, which in seven cases coincided with excessive grooming, scratching, and scratch marks. No cause was found for the other three cases. Data of these animals obtained prior to termination were not excluded for analysis, since including or excluding these measurements had virtually no effect on the mixed models. Readers who wish to examine the difference in modeling results may consult Table S1 and the corresponding matlab scripts.

### Plasma metabolite concentrations

Plasma TG was measured using a commercially available kit (Roche Diagnostics). Plasma insulin was measured by ELISA (Alpco). Glucose was measured on whole blood using an Accu‐Check^®^ glucose meter. Plasma TC and free cholesterol were measured by a colorimetric assay according to the CHOD‐PAP method (Allain and Poon 1974). HDL‐c was measured by measuring total cholesterol in the supernatant after precipitating apoB‐containing lipoproteins with polyethylene glycol 6000 (PEG6000) (Izzo et al. 1981).

### Hepatic metabolite concentrations

Liver lipids were extracted using a Bligh and Dyer (1959) procedure. Liver lipids were then redissolved in 2% Triton‐X100 and measured using a commercially available kit for measurement of triglyceride (Roche) and the CHOD‐PAP method for TC and free cholesterol measurement (Allain and Poon 1974). Phospholipids after Bligh and Dyer extraction were measured according to Bottcher et al. (1961).

### RNAseq analysis

RNA was isolated using a commercially available kit (RNeasy, Qiagen). Initial quality check and RNA quantification of the samples was performed by capillary electrophoresis using LabChip GX (Perkin Elmer). Nondegraded RNAsamples were used for subsequent sequencing analysis. Sequence libraries were generated using the 3′QuantSeq sample preparation kits (Lexogen). The obtained cDNA fragment libraries were sequenced on an Illumina HiSeq2500 using default parameters (single read 1 × 50 bp) in pools of multiple samples. The obtained fastQ files were aligned to build Mus_musculus.GRCm38 ensembleRelease 82 reference genome using hisat/0.1.5‐beta‐goolf‐1.7.20 (Dobin et al. 2013) with default settings. Before gene quantification, SAMtools/1.2‐goolf‐1.7.20 was used to sort the aligned reads (Li et al. 2009). The gene level quantification was performed by HTSeq‐count HTSeq/0.6.1p1 using –mode=union (Anders et al. 2014). Analysis of gene expression was performed in R (Law et al. 2016). Counts were normalized with the trimmed‐mean of *M*‐value normalization (TMM) method provided in the edgeR package (Robinson and Oshlack 2010). Analysis of differential gene expression was done using voom transformation provided by the limma package (Law et al. 2014). A false discovery rate of 0.05 and a log 2 fold change of 1 were taken as cutoff values for differentially expressed genes. To account for a batch effect introduced during RNA isolation, this was included in the design of the linear model. For visualization of batch‐controlled gene expression, batch effect was removed using the ComBat function of the R SVA‐package (Leek et al. 2012). Plots of gene expression in this article were produced by making use of the R packages ggplot2 (Wickham 2011) and superheat (Barter 2016).

### Liver histology

Paraffin sections were stained with hematoxylin and eosin stain and Sirius Red. The slides where then scored for NASH according to Kleiner et al. (2005). The percentage of fibrosis was determined as follows: Three x4 digital images were obtained randomly from every section using the polarized light option. The three pictures covered approximately between 70% and 90% of the specimen's total surface area. The images were imported to ImageJ and subsequently analyzed using an in‐house developed macro which was written to quantify the area, percentage, and integrated density of positively stained collagen fibers in every image using a camera specification with 226.7 msec exposure, sensitivity set to ISO 200, and snapshot resolution of 2560 × 1920.

### VLDL‐TG production

Animals were injected intraperitoneally with poloxamer 407, an inhibitor of lipoprotein lipase, at a dose of 1000 mg/kg BW (Millar et al. 2005). After 30, 60 and 180 min, a small volume of blood was collected for the measurement of triglycerides. VLDL‐TG production was then calculated by multiplying the slope of increased TG concentration by the theoretical plasma volume based on their body weight, where a blood volume of 75 mL/kg is assumed with a hematocrit of 0.45.

### Hepatic de novo lipogenesis

Animals were provided with drinking water containing 2% C^13^‐acetate at 9 AM. Food was removed the following day at 9 AM and animals sacrificed at 1 PM. Liver lipids were isolated using the Bligh and Dyer procedure. Isolated lipids were derivatized in acidic methanol at 90°C for 4 h. Mass‐isotopomer distribution of methylated fatty acids was then measured by LC–MS. Hepatic de novo lipogenesis and elongation was calculated as described previously (Hellerstein et al. 1993; Hellerstein and Neese 1999; Oosterveer et al. 2009).

### Endogenous glucose production

Endogenous glucose production was determined as described previously with minor modifications (van Dijk et al. 2013): Food was removed at 9 AM and the experiment started at 1 PM. At 1 PM, animals received 0.1 mg/g [6,6]‐D2‐glucose intraperitoneally at a concentration of 30 mg/mL. Immediately before, and 10, 20, 30, 40, 50, 60, 75, and 90 min after the intraperitoneal injection, a small amount of blood was collected on a filter paper and glucose measured with an Accu‐Check^®^ glucose meter. Glucose was then extracted from the dried blood with a water/ethanol mixture. After the solution was evaporated under nitrogen flow at 60°C, the residue was derivatized to glucose penta‐acetate by adding 100 *μ*L pyridine and 200 *μ*L acetic anhydride to the extracted glucose and heating at 60°C for 30 min. After evaporation under nitrogen flow, the residue was then dissolved in 200 *μ*L ethyl acetate for analysis by GC–MS. GC–MS was performed with a Zebron ZB‐1701 30 m × 250 *μ*m × 0.25 *μ*m (Phenomenex) column under positive chemical ionization with ammonia ions monitored at *m*/*z* 408–412 (*m*_0_–*m*_4_). After corrections for natural abundances according to Lee et al., the kinetics of the enrichment of glucose with D2‐glucose were then used to estimate endogenous glucose production and related parameters according to van Dijk et al. (2013).

### Bile metabolite concentrations

Gallbladder cannulation was performed under fentanyl–fluanisone anesthesia (1 mL/kg). After allowing the gallbladder to empty for 5 min, bile was collected for 20 min while mice were placed in an incubator to maintain body temperature. Bile production was measured by weighing the collected bile. Quantification of biliary bile acids was performed as follows. Bile samples were 1000× diluted with MilliQ. After homogenization, 25 *μ*L diluted bile was aliquoted into a clean tube for bile acid analysis. For every 10 samples prepared, one quality control standard plasma was included. To each sample 250 *μ*L internal standard solution was added and vortexed for 1 min. D4‐cholic acid, D4‐chenodeoxycholic acid, D4‐glycochenodeoxycholic acid, D4‐glycocholic acid, D4‐taurochenodeoxy‐cholic acid, and D4‐taurocholic acid were used as internal standards. Samples were then centrifuged at 15,800*g* and the supernatant transferred to a clean glass tube. The fluid was evaporated under nitrogen at 40°C. If samples were not measured immediately, they were stored at −20°C until further analysis. Samples were then reconstituted in 200 *μ*L 50% methanol in water, vortexed for 1 min, and centrifuged for 3 min at 1800*g*. The supernatant was transferred into a 0.2 *μ*m spin‐filter and centrifuged at 2,000*g* for 10 min. After filtering, the samples were transferred into LC–MS vials and analyzed (10 *μ*L injection volume). For separation, a Nexera X2 Ultra High Performance Liquid Chromatography system (Shimadzu, Kyoto, Japan), coupled to a SCIEX QTRAP 4500 MD triple quadrupole mass spectrometer (SCIEX, Framingham, MA) (UHPLC‐MS/MS) was used, with an ACQUITY UPLC BEH C18 Column (1.7 *μ*m × 2.1 × 100 mm) equipped with a ACQUITY UPLC BEH C18 VanGuard Pre‐Column (1.7 *μ*m × 2.1 × 5 mm) (Waters, Milford, MA). Separation was achieved in 28 min using 10 mmol/L ammonium acetate in 20% acetonitrile (mobile phase A) and 10 mmol/L ammonium acetate in 80% acetonitrile (mobile phase B), total flow rate: 0.4 mL/min. To quantify biliary lipids, lipids were isolated from 15 *μ*L of bile using Bligh and Dyer (1959) extraction and split in 10 *μ*L for cholesterol measurement via a homemade assay according to Gamble et al. (1978) and 5 *μ*L for phospholipid measurement according to Bottcher et al. (1961).

### Feces metabolite concentrations

Neutral sterols and bile acids were measured by GC–MS as described previously (van Meer et al. 2008), with a minor modification, where fecal neutral sterols are extracted from 50 mg of feces with 2 × 2 mL petroleum ether after saponification in 1 mL of alkaline methanol, instead of 3 × 3 mL.

### Statistical analysis

While we aimed for a fully longitudinal study, some parameters are currently not accessible for multiple measurements in the same animal, since they require terminating the animal. Such cross‐sectional data between different time points were compared with one‐way ANOVA, or when a non‐parametric test was indicated, by a Kruskal–Wallis test and the *P*‐value adjusted for multiple comparisons by Tukey–Kramer correction. For all truly longitudinal data, mixed‐linear effects models were used to establish whether data were best described as biphasic or as either linearly increasing or decreasing, using the MATLAB and Statistics Toolbox Release 2015b software. Values versus fitted‐value plots were used to check for the presence of heteroscedasticity and non‐normal distribution of the residuals. When the model was best described as biphasic, best linear unbiased estimates were used to calculate the peak of the fitted model. To calculate marginal and conditional *R* ^2^, we made use of the formulas described by Johnson (2014).

$$\text{marginal}\;R^{2}={\text{var}}_{-}\text{fixed}/\left({\text{var}}_{-}\text{fixed}+{\text{var}}_{-}\text{random}+{\text{var}}_{-}\text{resid}\right)$$

$$\text{conditional}\;R^{2}=\left({\text{var}}_{-}\text{fixed}+{\text{var}}_{-}\text{random}\right)/\left({\text{var}}_{-}\text{fixed}+{\text{var}}_{-}\text{random}+{\text{var}}_{-}\text{resid}\right)$$

In the setting of a mixed‐effect model, the marginal *R* ^2^ reflects the variation explained by the fixed‐effects of the linear model. In our case, by using time as the only variable, it reflects how much of the variation is explained by the coefficients that fit the group mean response through time. The conditional *R* ^2^, in contrast, reflects how much of the variation in time is explained when including the individual response to the diet. That is, when allowing coefficients for individual mice to be normally distributed around the estimate for the group mean response.

## Results

### Body weight and food intake

**Figure 1:**
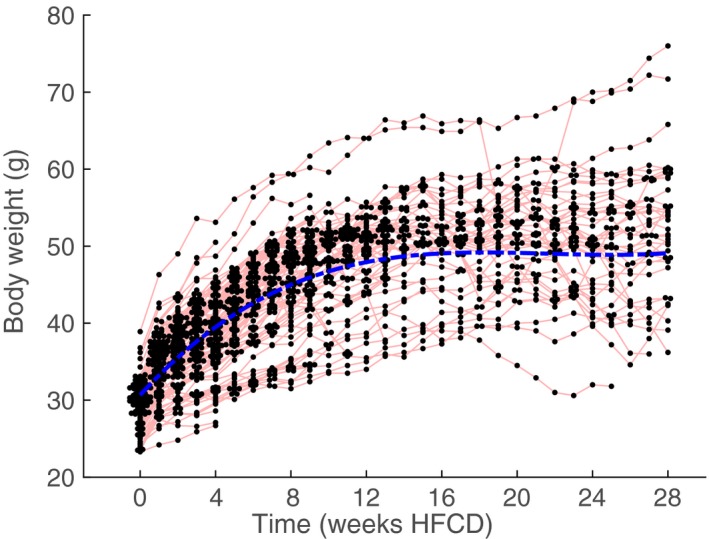
Body Weights of male E3L.CETP mice on HFCD over time. Note that body weight plateaus around 12–15 weeks. Blue‐dotted lines represents the group mean response of the mixed model. Red lines connect measurements performed on the same individual animal. Mixed modeling suggests a maximum at 18 weeks HFCD.

**Figure 2:**
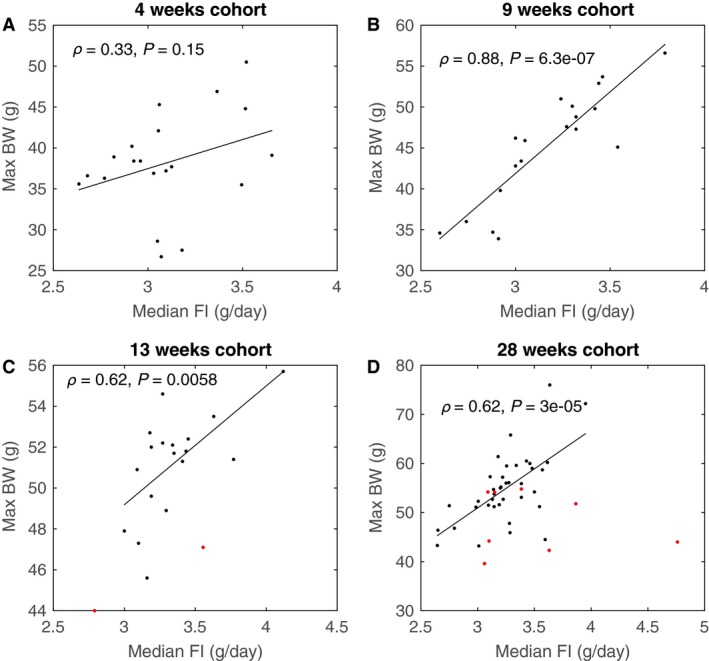
Correlations between individual median food intake over time with the maximally attained body weight of the respective cohorts. Red points indicate animals that dropped out before the end of the experimental period. Pearson correlations shown are with dropouts excluded. When including dropouts correlations are *ρ* = 0.62 (*P* = 0.0037) and *ρ* = 0.24 (*P* = 0.10) for the 13 and 28 weeks cohort, respectively.

Body weight rapidly increased in response to the HFCD and then plateaued around 12–16 weeks (Fig. 1). Mixed modeling suggests a maximum at 18 weeks, with a marginal and conditional *R* ^2^ of 0.47 and 0.98, respectively, illustrating the large variation in body weight between animals. Food intake was higher the first few weeks after introduction to the diet. Toward the end of the diet some animals showed increased food intake as well. We note that food intake is difficult to determine accurately, as the food pellets are crumbly and some animals are prone to spillage, especially immediately after introduction to the diet (Fig. S1). When applying a mixed model to the food intake, we find a better fit for a biphasic curve where the marginal response indicates a minimum at 13 weeks with a week‐averaged daily food intake of 3.2 g and a daily food intake of 3.7 and 3.8 g at the start and end of the experiment, respectively. These results should be interpreted with caution since the marginal *R* ^2^ is only 0.03. Median daily food intake was 3.1, 3.2, 3.3, and 3.2 g for the 4, 9, 13, and 28 week cohorts, respectively. We noted that individual median food intake correlated with maximum body weight attained for the 9, 13 and 28 week cohort (Fig. 2) but not for the 4 week cohort. The correlations of the 28 week cohort are only significant when drop outs are excluded. All in all, these results indicate that at least part of the variation in body weight observed may be explained by individual differences in food intake.

### Plasma lipid parameters

**Figure 3:**
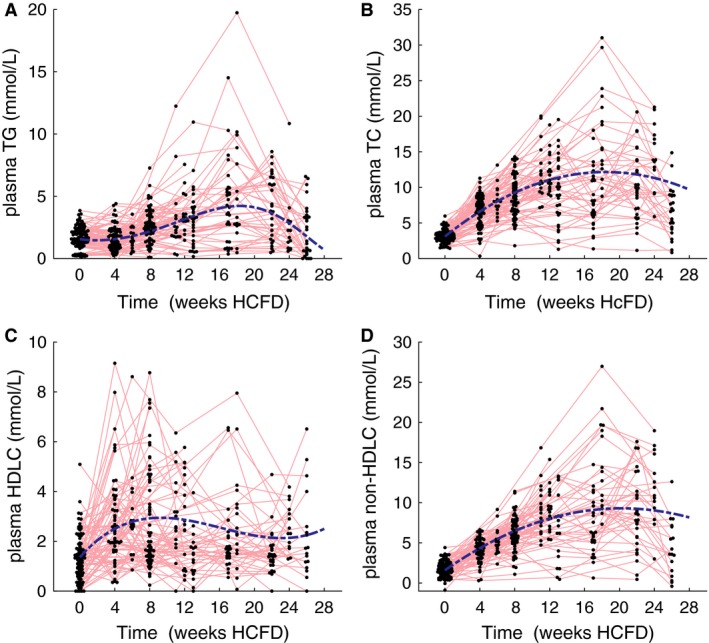
Plasma triglyceride (A), total cholesterol (B), HDL‐C (C) and non‐HDLC (D), for male apoE*3L.CETP mice as measured at the indicated time points. Data for the respective cohorts was pooled when measured on the same time point. Blue‐dotted lines represents the group mean response of the mixed model. Red lines connect measurements performed on the same individual animal.

Plasma triglyceride (Fig. 3A) and plasma total cholesterol (Fig. 3B) showed considerable changes and an interesting biphasic response to the HFCD. Inspection of the data in Fig. 3 suggests a maximum in plasma triglyceride and cholesterol between 16 and 20 weeks after start of the diet. To test and quantify these observations, mixed linear effect models were used. Using time as the sole predictor variable, we found that for plasma triglyceride and total cholesterol model specifications with third power and quadratic fixed effects, respectively achieved a better fit to the data than the corresponding mixed effect models with time as a linear predictor. This supports that the observed response in plasma triglyceride and total cholesterol is indeed biphasic. Plasma TG was transformed by taking the square root to mitigate heteroscedasticity in the residuals. We found a marginal and conditional *R* ^2^ of 0.19 and 0.9 for plasma triglyceride and 0.50 and 0.87 for plasma total cholesterol, underlining that the random effects (i.e., individual contribution) explain a large proportion of the variance. The terms of the fixed effects point to a maximum at 18 weeks for both plasma triglyceride and total cholesterol. Inspection of the HDL‐c data does not convey a clear trend through time (Fig. 3C). However, similar to plasma TG, mixed modeling results in the best data fit for third power model, leading to a marginal and conditional *R* ^2^ of 0.14 and 0.57, respectively and a maximum at 9 weeks. Consistent with the findings for plasma TC, mixed modeling of non‐HDL‐c shows a maximum at 20 weeks, with a marginal and conditional *R* ^2^ of 0.51 and 0.83, respectively (Fig. 3D).

### Plasma glucose and insulin

**Figure 4:**
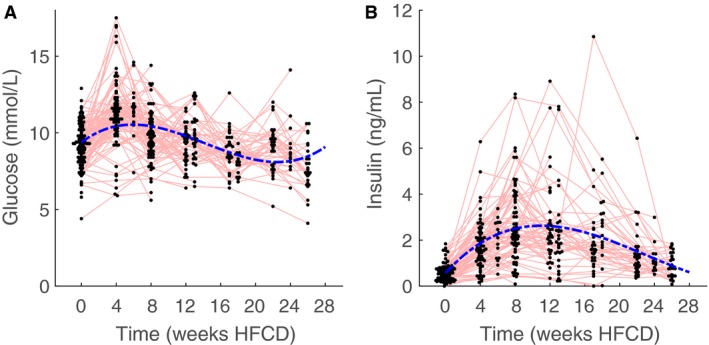
Whole blood glucose (A) and plasma insulin (B) of male apoE*3L.CETP mice as measured at the indicated time points. Data for the respective cohorts was pooled when measured on the same time point. Blue‐dotted lines represents the group mean response of the mixed model. Red lines connect measurements performed on the same individual animal. Note the biphasic response and that glucose peaks earlier than plasma insulin levels.

In line with effects on TG and cholesterol levels, plasma glucose and insulin also reveal a biphasic response (Fig. 4), yet the response peaked much earlier and was apparently not directly coupled to changes in lipids. Mixed modeling shows that for both glucose and insulin a third power mixed effect model fits the data best, with a peak for glucose at 6 weeks and a peak for insulin at 10 weeks. Marginal and conditional *R* ^2^ for plasma glucose and insulin were 0.18 and 0.28, and 0.28 and 0.8, respectively.

### Liver lipid content

**Figure 5:**
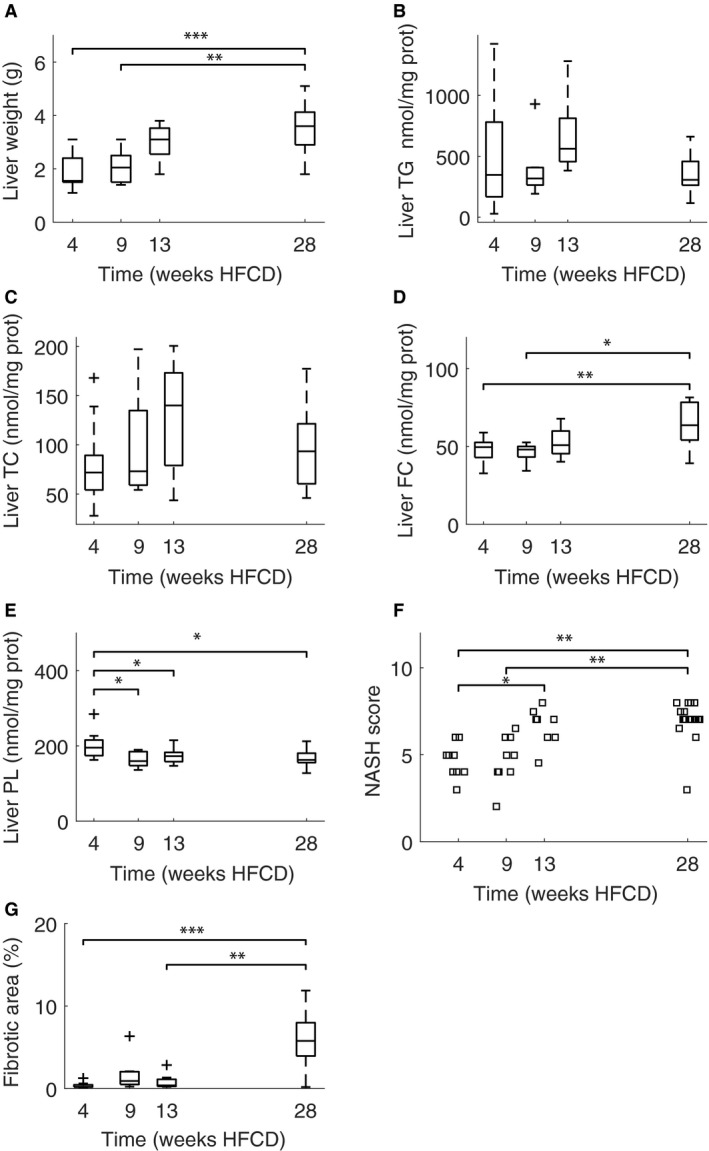
Liver weights (A), liver triglyceride (B), total cholesterol (C), free cholesterol (D) and phospholipid (E) concentrations, and NASH scores (F) and fibrotic area (G) of livers in male apoE*3L.CETP cohorts sacrificed after 4, 9, 13, and 28 weeks HFCD, respectively. NASH scores show a statistically significant difference both between 4 weeks and 13 (*P* = 0.047) and 28 weeks (*P* < 0.01) and between 9 weeks and 28 weeks (*P* < 0.01), using the one‐way Kruskal–Wallis test. Fibrotic area was remarkably similar for the cohorts sacrificed up to 13 weeks while fibrotic area after 28 weeks HFCD diet was notably increased (*P* < 0.01). *N* = 10, 10, 9 and 21 for 4, 9, 13, and 28 weeks HFCD diet, respectively. Significant differences are denoted with brackets and asterisks: *P* < 0.05 (*), *P* < 0.01 (**), *P* < 0.001 (***).

Liver weight increased with the time spent on the HFCD (Fig. 5A). There appears a trend for liver TG to first increase and then decrease. However, even the most marked difference in liver TG between 13 weeks and 28 weeks does not reach statistical significance (*P* = 0.053) (Fig. 5B). Similarly, liver total cholesterol follows the trend of liver triglycerides (Fig. 5C). One‐way ANOVA shows no significant differences. Liver free cholesterol remains constant through time, with a trend for an increased free cholesterol concentration at the latest time point (Fig. 5D). One‐way ANOVA shows that the difference between 28 weeks and both 4 (*P* = 0.01) and 9 weeks (*P* = 0.02) were statistically significant. In contrast, liver phospholipids show a trend for being decreased at the last time point when compared with the first time point (Fig. 5E). One‐way ANOVA shows that the liver phospholipid concentrations after 4 weeks HFCD differed significantly from 9 weeks (*P* = 0.01), 13 weeks (*P* = 0.04), and 28 weeks (*P* = 0.02).

### Liver NASH scores

Liver NASH scores and fibrotic area increased with time spent on HFCD (Fig. 5F–G). NASH scores show a statistically significant difference both between 4 weeks and 13 (*P* = 0.047) and 28 weeks (*P* < 0.01) and between 9 weeks and 28 weeks (*P* < 0.01), using the one‐way Kruskal–Wallis test. Fibrotic area was remarkably similar for the first 3 months while fibrotic area after 28 weeks HFCD was notably increased (*P* < 0.01).

### RNAseq analysis

**Figure 6:**
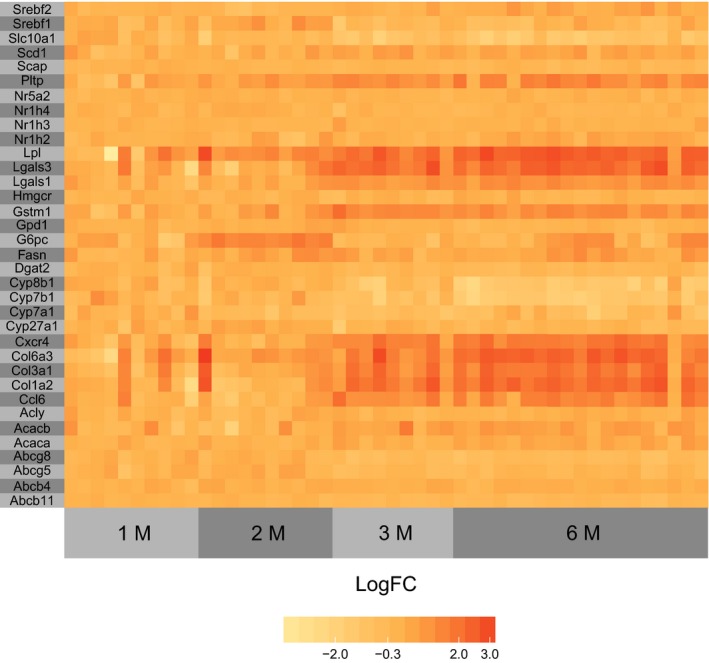
Heatmap of selected gene expressions over time.

To investigate the effect of 6 months HFCD feeding on gene expression in the metabolic networks of the mice, we determined global mRNA levels in the livers using the Lexogen platform. The number of differentially expressed genes after 9 weeks, 13 weeks, and 28 weeks HFCD diet (compared with 4 weeks HFCD) was surprisingly low; 1, 63 and 159, respectively, against 6843 genes with sufficient sequencing depth. Of differentially expressed genes, many were associated with inflammation and fibrosis and increased in expression over time. For example, the collagen subunits *Col1a1, Col1a2, Col3a1, Col6a3* and the chemokines *Ccl6, Cxcr4* were all increased in expression at 13 weeks and 28 weeks HFCD. Furthermore, we note high expression of glutathion transferases (*Gstm1*,* Gstm3*) and galectins (*Lgals1*,* Lgals3*) at 13 and 28 weeks of HFCD. When focusing on differentially expressed genes with roles in metabolism we note the following. *G6pc* expression was increased (logFC = 1.4, FDR = 0.04) in the 9 weeks HFCD cohort, *Lpl* expression was increased in the 13 week cohort (logFC = 1.4, FDR < 0.001) and 28 week cohorts (logFC = 1.9, FDR < 1e‐6), *Pltp* expression was increased in the week 13 cohort (logFC = 1, FDR < 0.001) and at 28 weeks HFCD (logFC = 1.1, FDR < 1e‐5), while *Cyp7b1* (logFC = −1.5, FDR < 1e‐6), *Cyp8b1* (logFC = −1.5, FDR < 1e‐6), and *Slc10A1* (logFC = −1.3, FDR < 1e‐6) expression was decreased after 28 weeks HFCD. Although it may be expected that many genes important in the regulation of cholesterol homeostasis (*Nr1 h3*,* Nr1 h2*,* Nr5a2*,* Srebf2*,* Insig*,* Scap*,* Hmgcr*,* Abcg5*,* Abcg8*) and of triglyceride synthesis (*Srebf1*,* Fasn*,* Dgat2*,* Acly*,* Acaca*,* Acacb*,* Scd1*,* Gpd1*) would be differentially expressed over time, this was actually not the case. A heat map is provided with the genes mentioned above (Fig. 6), a full list of differentially expressed genes over time may be found in a supplemental file (Suppl. File *RNA_analysis*).

### VLDL‐TG production and hepatic de novo lipogenesis

**Figure 7:**
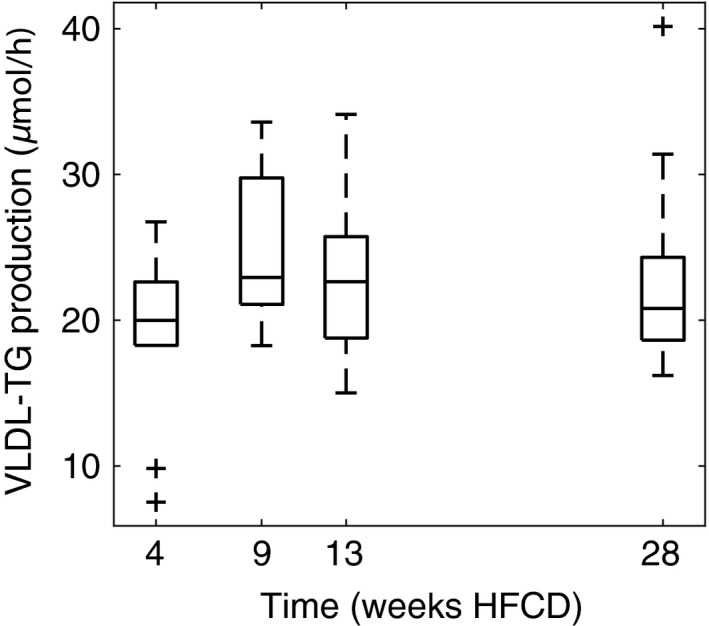
VLDL‐TG production in the respective cohorts does not convey a clear up or downward trend. One‐way ANOVA shows no significant differences between the time points. *N* = 10, 9, 9 and 16 for 4, 9, 13, and 28 weeks HFCD diet, respectively.

**Figure 8:**
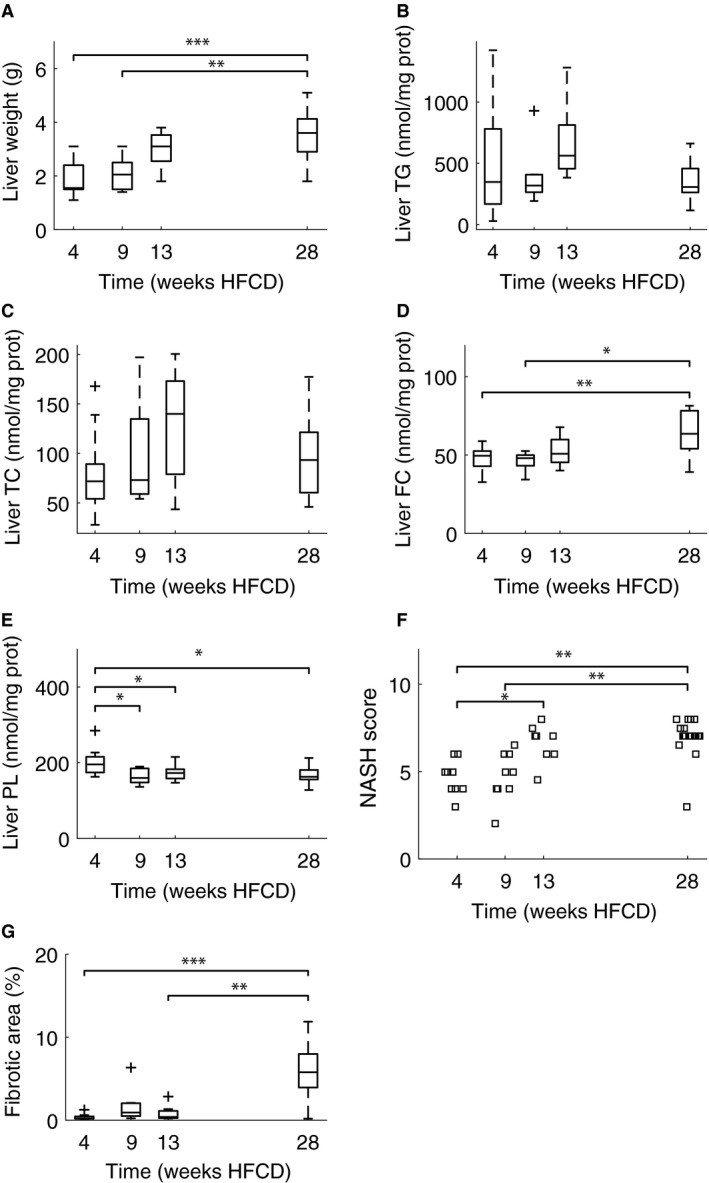
Fractional de novo lipogenesis for palmitate (C16:0) (A), stearate (C18:0) (B), oleate (C18:1) (C), and chain elongation of palmitate to stearate (C18:0) (E) and to oleate (C18:1) (F), across the respective cohorts. *N* = 10, 9, 10 and 17 for 4, 9, 13 and 28 weeks HFCD diet, respectively. Significant differences, according to one‐way ANOVA, are denoted with brackets and asterisks: *P* < 0.05 (*), *P* < 0.01 (**), *P* < 0.001 (***).

In spite of both liver lipid content and histology showing large changes over time, we found no differences in VLDL‐TG production. VLDL‐TG production was 162 (46), 176 (29), 151 (44) and 136 (54) *μ*mol TG/kg/h for 4, 9, 13 and 28 weeks, respectively (SDs are given within brackets), thus showing no clear trend over time (Fig. 7). Fractional de novo lipogenesis (i.e., the percentage of newly synthesized fatty acids) appears higher at 13 and 28 weeks, with significant differences between 4 and 9 weeks on one hand and 13 and 28 weeks on the other hand for de novo production of palmitate (C16:0, Fig. 8A) and stearate (C18:0, Fig. 8B). Oleate (C18:1, Fig. 8C) fractional de novo lipogenesis was also significantly different between 9 weeks compared with 13 and 28 weeks. Fractional de novo lipogenesis attributable to chain elongation of palmitate (Fig. 8D–E) shows a mixed picture, with stearate contribution of chain elongation being different only between 9 weeks and the 4, 13, and 28 week time points, whereas the oleate contribution to chain elongation showed significant differences between 4 and 28 weeks.

### Endogenous glucose production

**Figure 9:**
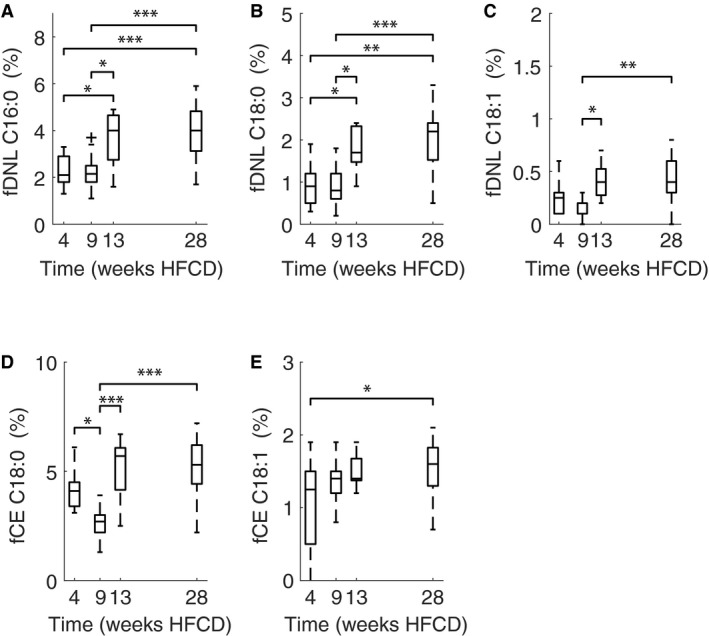
Steady state glucose (A), post‐test plasma insulin (B), endogenous glucose production rate (C) of male E3L.CETP mice on HFCD. From these values we can calculate insulin sensitivity (D), peripheral insulin sensitivity (E) and hepatic insulin sensitivity (F). Note that peripheral insulin resistance decreases and increases over time while hepatic insulin resistance does not. Blue‐dotted lines represents the group mean response of the mixed model. Red lines connect measurements performed on the same individual animal. *n* = 16, 16, 15 and 14 for 3, 9, 15 and 27 weeks, respectively.

Tracer kinetics showed a trend for decreasing AUC and mean residence time over time, whereas for bioavailability, apparent volume of distribution, metabolic clearance rate, and glucose pool size the parameters decreased from 3 to 15 weeks HFCD followed by an increase at the 27‐week time point (Fig. S3). Similarly, and in line with the decreasing trend for plasma glucose and the behavior of the metabolic clearance rate with time spent on the HFCD (Fig. 9A), it was seen that endogenous glucose production decreased over time during the first 3 months, followed by an increase toward 27 weeks of HFCD (Fig. 9B). We observed no trend for overall insulin sensitivity (Fig. 9D). However, peripheral insulin sensitivity showed a decrease with a minimum around 9 weeks followed by an increase towards 27 weeks. In contrast, the hepatic insulin sensitivity showed a very mixed picture, with values diverging the most at 9 weeks HFCD and then converging slightly at 27 weeks HFCD. Mixed modeling suggests a minimum of endogenous glucose production at 17 weeks of HFCD. Marginal and conditional *R* ^2^ were 0.53 and 0.53, respectively. Peripheral insulin sensitivity showed a minimum at 15 weeks and marginal and conditional *R* ^2^ of 0.16 and 0.47, respectively.

### Biliary bile acid and cholesterol secretion

**Figure 10:**
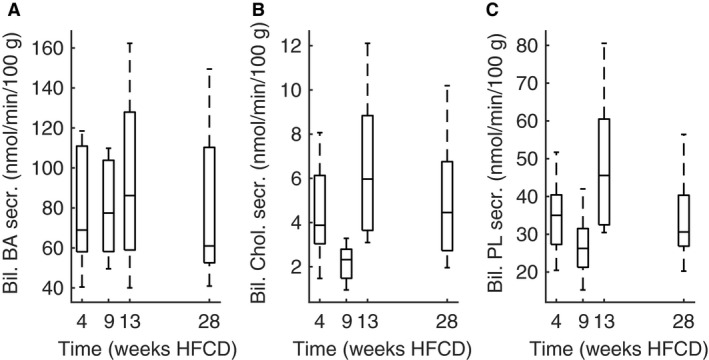
Biliary bile acids (A), biliary neutral sterol (B) and biliary phospholipid secretion rate (C) in male E3L.CETP mice on HFCD diet for 4 weeks (*n* = 10), 9 weeks (*n* = 10), 13 weeks, (*n* = 9), or 28 weeks (*n* = 16), respectively.

In line with the biphasic curve observed for plasma and liver lipids, biliary bile acid production, cholesterol production, and phospholipid production also show a biphasic trend (Fig. 10). One‐way ANOVA shows that differences over time for bile acid production do not reach statistical significance. Biliary cholesterol production is different between 9 and 13 weeks (*P* < 0.001), and 28 weeks (*P* = 0.03). Biliary phospholipid production is also different between 9 and 13 (*P* = 0.002) on one hand and between 13 and 28 weeks (*P* = 0.04) on the other hand.

### Fecal neutral sterol and bile acid excretion

**Figure 11:**
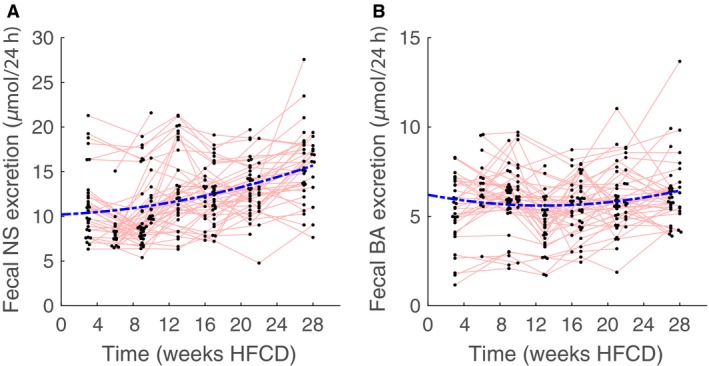
Fecal neutral sterol (NS) and bile acid excretion in male E3L.CETP mice on HFCD diet at 3 weeks (*n* = 30), 6 weeks (*n* = 16), 9 weeks (*n* = 30), 10 weeks (*n* = 16), 13 weeks (*n* = 30), 16 weeks (*n* = 15), 17 weeks (*n* = 29), 21 weeks (*n* = 29), 22 weeks (*n* = 14), 27 weeks (*n* = 24) and 28 weeks (*n* = 14). The blue‐dotted line represents the group mean response of the mixed model. Red lines connect measurements performed on the same individual animal.

Fecal neutral sterol excretion appears to increase with time. In contrast, fecal bile acid excretion does not show an obvious trend over time (Fig. 11). Mixed linear modeling indicates, however, that in both cases a quadratic model is appropriate. We then arrive at a minimum of fecal neutral sterol excretion at minus 4 weeks, indicating that the group mean response only increases in the experimental time‐frame, and a minimum of bile acid production at 9 weeks. Marginal and conditional *R* ^2^ for fecal cholesterol excretion was found to be 0.18 and 0.75, respectively, whereas marginal and conditional R^2^ for fecal bile acid production was 0.02 and 0.69, respectively. Clearly, there is a large individual contribution to variation in fecal sterol excretion.

## Discussion

ApoE*3L.CETP mice fed an HFCD show a biphasic course for plasma TG, TC, HDL‐c, glucose, insulin, endogenous glucose production and peripheral insulin sensitivity, which at least for the changes in plasma lipids, is reminiscent of similar observations in cross‐sectional studies in man. At the same time we must note that the response of animals to the HFCD is extremely heterogenic for which the reasons are not known. We further found no change in VLDL‐TG production between the different time points. This indicates that differences in plasma lipids over time are likely the result of changes in clearance. Another interesting observation was that plasma insulin peaked before plasma TG peaked, which suggests that these two hallmarks of metabolic syndrome are not similarly linked to body weight gain over time.

### Inter‐individual variation in response to HFCD is large in E3L.CETP mice

A large degree of inter‐individual variation was observed in this study. The mouse strain used here, E3L.CETP and the related apoE*3‐Leiden strain, are known for their heterogeneity in response to Western‐type diets. In fact, animals that do not respond to a diet containing 16% fat and 0.1–0.25% cholesterol with respect to a sufficient raise in TC are usually excluded from subsequent studies on atherosclerosis development. While we cannot currently explain the heterogeneity, the observation that median food intake correlated with the maximum attained body weight, suggests that part of the observed heterogeneity may be attributed to individual differences in food intake. Furthermore, it should be noted that phenotypic heterogeneity is also observed in its background strain C57BL/6J, and therefore not solely attributable to the transgenes in E3L.CETP. C57BL/6J non‐uniform response to high‐fat diets have been brought to the attention before, and has prompted researchers to stratify individuals for subsequent study (Burcelin et al. 2002; Peyot et al. 2010). In fact, homozygosity in inbred mice has long been known to increase variability (Phelan and Austad 1994).

### Biphasic response in plasma insulin and peripheral insulin sensitivity

We found that plasma insulin increases on HFCD and peaks early, while notably peripheral insulin sensitivity, as derived from stable‐isotope tracer studies, increased over time after an initial decrease. The majority of studies in mice find increased insulin resistance with age on high‐fat diet (Kleemann et al. 2010). However, decreasing insulin resistance with age beyond 24 weeks of high‐fat diet has also been observed in C57BL/6J‐mice (Van Der Heijden et al. 2015). Cross‐sectional studies in humans show that insulin resistance increases with age (Janssen et al. 2000; Dubowitz et al. 2014; Murakata et al. 2015). In our study overall insulin sensitivity did not change while peripheral insulin sensitivity increased in the second phase, indicating that E3L.CETP mice on HFCD do not behave similar to humans with regard to changes in insulin resistance beyond approximately 4 months of HFCD. It is unclear what caused the increased peripheral insulin sensitivity in this study and whether this finding is specific for this mouse strain or a more general phenomenon of mice subjected to HFCD for longer periods of time. Assuming the latter, a potential mechanism may be that over the course of the HFCD, cholesterol and oxysterol metabolites accumulate in adipose tissue and lead to increased activation of NR1H3 (a.k.a. LXR), in turn leading to increased peripheral insulin sensitivity (Baranowski 2008).

### Biphasic response in dyslipidemia reminiscent of decrease in plasma TG in mouse models of accelerated aging

We observed a biphasic response in dyslipidemia to HFCD with plasma TG and TC. Data regarding the development of dyslipidemia in E3L.CETP mice on HFCD over an extended time period have not previously been reported. However, a longitudinal study in apoE3*Leiden mice on a high‐fat diet without added cholesterol up to 3 months showed a gradual increase in plasma TG and plasma TC, which is in agreement with the current study (Kleemann et al. 2010). Interestingly, plasma TG in the *Ercc1* ^*−/*Δ^ mouse model of accelerated aging at 21 weeks of age is similar to wild‐type mice aged 26–34 months, and decreased compared to 7‐week‐old wild‐type mice (Gregg et al. 2012). In other words, a decrease in plasma TG in mice is associated with aging, and the observed changes in lipoprotein metabolism of E3L.CETP mice on HFCD may share some mechanisms with those active in the aging process.

### VLDL‐TG production does not explain biphasic pattern of dyslipidemia

In this study, VLDL‐TG production was not significantly different between measured time points, suggesting that the differences observed in dyslipidemia are likely due to differences in the clearance of apoB‐lipoproteins. In contrast, earlier studies in apoE3*Leiden mice on a HFCD showed increased VLDL‐TG production leading to a peak at 45 days compared to 100 days of age (van Vlijmen et al. 1996). In our experiment, however, animals were aged >100 days at the start of the experiment. Together these findings suggest that VLDL‐TG production in apoE3*Leiden and E3L.CETP mice are increased at a younger age on a HFCD, however, may decrease and stabilize to a steady value once animals reach maturity. Given that VLDL‐TG production was not changed over time, the observed changes in plasma TG are likely to be the result of changes in triglyceride hydrolysis activity. VLDL‐TG production as primary cause for variation in plasma TG can, however, not be completely excluded, given the heterogeneity observed between animals and that with the currently available methods, VLDL‐TG production in an animal can be measured only once. However, the fact that we observed no correlation between basal plasma TG and VLDL‐TG production rate (Fig. S4) argues against this possibility.

### Contribution of VLDL‐production and clearance to dyslipidemia in humans

Since E3L.CETP mice have a “humanized” lipoprotein metabolism, it is of interest to compare findings in lipid parameters of this study to those found in humans. Whereas in this study differences in dyslipidemia appear to be mediated by differences in lipoprotein clearance, dyslipidemia observed in metabolic syndrome in men is considered to be caused by an increase in VLDL‐production (Adiels et al. 2008). Furthermore, increased age is associated with increased VLDL‐production as well as reduced LDL‐clearance in men (Millar et al. 1995). On the other hand, studies in women and mixed groups suggest that age‐related increases in triglycerides and cholesterol are due to decreased catabolism of lipoproteins (Pinto et al. 2001; Matthan et al. 2005). All in all, we must conclude that in humans both VLDL‐TG production and clearance can contribute to differences in plasma TG. Although a limitation of this study was that triglyceride hydrolysis activity as such was not evaluated, the lack of significant differences in VLDL‐TG production over time suggests that changes in triglyceride hydrolysis activity was the predominant factor in the dyslipidemia observed here.

### Gene expression profiles do not follow the response to HFCD in metabolic fluxes

Gene expression analysis of E3L.CETP mice on HFCD for 4, 9, 13, and 28 weeks showed an increase in expression of genes involved in inflammation and fibrosis. Furthermore, we observed that metabolic differentially expressed genes encoding metabolic enzymes showed little correlation with the measured metabolic fluxes. First, the increased G*6pc* expression after 2 months HFCD does not coincide with increased endogenous glucose production. It should be noted that *G6pc* expression is mostly linked to increased glucose production in primary hepatocytes, whereas in vivo studies show that changes in G*6pc* expression do not necessarily result in changes in endogenous glucose production (Van Dijk et al. 2001; Doi et al. 2007). Secondly, a decrease in *Cyp7b1*, C*yp8b1*, and S*lc10a1* in expression over time, does not coincide with a concomitant decrease in biliary bile acid secretion. This is in agreement with earlier findings where changes in bile acid gene expression may affect composition of bile acids but has little bearing on the total biliary bile acid secretion (Sokolovic et al. 2010; Boesjes et al. 2014). Finally, absence of *Pltp* has previously been shown to impair VLDL‐TG production in mice (Yazdanyar and Jiang 2012). Here, we observed that *Pltp* was decreased in expression over time, with, however, no changes in VLDL‐TG production. Overall, the findings in this study may serve as another reminder that gene expression can generally be considered as a poor predictor of metabolic fluxes (Daran‐Lapujade et al. 2007; Morandini 2009, 2013).

### Linear relation between body weight and liver weight

While the liver weight increased over time, hardly any significant changes through time on HFCD for the liver lipids were observed. Free cholesterol, however, increased with time, which is in line with other studies, in which free cholesterol accumulates in the liver on high‐fat high‐cholesterol diet and contributes to NASH development (Van Rooyen et al. 2011). Interestingly, we furthermore observed a near linear relation between body weight and liver weight, suggesting a redistribution of fat from adipose tissue to liver. In contrast, the relation between body weight and liver triglyceride concentrations is less clear.

### Modest increase in hepatic de novo lipogenesis over time

Changes in hepatic de novo lipogenesis were more evident, being increased at 13 and 28 weeks HFCD compared with 4 and 9 weeks time points. This is in line with other mouse studies where high‐fat diet stimulated hepatic de novo lipogenesis and this effect was stronger with longer time spent on high‐fat diet (Pierce et al. 2016). In human studies, the relation between age and hepatic de novo lipogenesis has received little attention to date, with one study reporting increased hepatic de novo lipogenesis in elderly compared to younger controls (Flannery et al. 2012). Furthermore studies are necessary to establish these relations.

### Biliary bile acid and cholesterol fluxes

We observed no clear pattern in biliary bile acid secretion over time. Dietary cholesterol increases bile acid production in mice within 3 weeks (Jolley et al. 2000). On the other hand, aging has been reported to decrease bile acid production in both the humans and mice (Einarsson et al. 1985; Wang 2002). In contrast, biliary cholesterol production increases with age in man (Einarsson et al. 1985). While we observed an increase in biliary cholesterol production between 28 weeks and 9 weeks, the difference between 28 weeks and 4 weeks HFCD was not statistically significant. All in all, there appears to be no aging effect in bile production in E3L.CETP mice on HFCD within 28 weeks.

### Role of cholesterol absorption in dyslipidemia in mice and humans

Dyslipidemia in mice is generally elicited through feeding HFCD, since high‐fat diet without added cholesterol is not sufficient (Kleemann et al. 2007). It follows that cholesterol absorption may be an important factor in the response to the HFCD. Here, we found that fecal neutral sterol excretion increased with time on HFCD, indicating that dietary cholesterol absorption of E3L.CETP mice on HFCD decreases as mice age. Of note, C57BL/6J‐mice on a standard diet show the opposite, that is, an increase in cholesterol absorption with increasing age (Duan et al. 2006). In humans, cholesterol absorption is an important predictor for dyslipidemia (Matthan et al. 2009; Cederberg et al. 2013). Moreover, a cross‐sectional study in Finland found that healthy 75‐year‐old men show decreased cholesterol absorption compared with 50‐year‐old men (Gylling et al. 1994). Taken together, it appears that E3L.CETP mice on a HFCD follow a similar trajectory in cholesterol absorption as humans. While at this point it is not clear whether these observations are caused by comparable mechanisms, it is intriguing that a “humanized” lipid profile may also lead to a “human” trajectory in intestinal cholesterol absorption.

### Insulin resistance precedes dyslipidemia

We observed in this study that insulin resistance preceded dyslipidemia. Similar observations have been made in other animal as well as human studies. Barnard et al. observed that first fasting insulin levels increased, that is, within 2 weeks, and then plasma TG increased in unison with glycerol release and fat cell size in female Fischer rats on high‐fat high‐sucrose diet (Barnard et al. 1998). Similarly, in the San Antonio Heart Study, fasting plasma insulin was shown to be predictive for increased plasma TG at 8 years follow‐up in humans (Haffner et al. 1992; Mitchell et al. 1992). Assuming that a common cause for insulin resistance and dyslipidemia exists, these findings imply that insulin resistance is triggered first and dyslipidemia only occurs beyond a threshold. The idea of a threshold is consistent with the paradigm of insulin resistance being caused by free fatty acids leaking into the system once the capacity of white adipose tissue to store fat is exceeded. Recently, van Beek et al. (2015) found that in C57Bl/6J mice, plasma TG increases sharply once mice exceed a body weight around 40 g. While here a similar pattern is observed (Fig. S5), the variation is higher, suggesting the presence of another factor that modulates plasma TG. Similarly, while van Beek et al. (2015) find a close relationship between body weight and plasma insulin, here we find that this relationship is less distinct, and that body weight in the first 3 months of HFCD is associated with higher plasma insulin levels than in the second 3 months of HFCD (Fig. S5). This change in relation between body weight and both insulin resistance and dyslipidemia implies that, if fatty acids are considered the culprit, a fatty acid sink must develop over time, mitigating both dyslipidemia and insulin resistance.

### In conclusion

Overall, E3L.CETP mice on a HFCD show a biphasic response in metabolic parameters through time, and may prove to be an adequate model to study age‐dependent changes in plasma lipids in men.

## Conflict of Interest

The authors declare no competing interests.

## Data Accessibility

## Supporting information




**Figure S1**. Food intake of E3L.CETP – mice on HFCD diet through time.
**Figure S2**. Liver weight correlates with body weight (*ρ* = 0.9, *P* > 0.001).
**Figure S3**. Tracer kinetics of male E3L.CETP mice that were used to measure endogenous glucose production: Mean residence time (A), Bioavailibility (B), AUC (C), apparent volume of distribution (D), metabolic clearance rate (E) and pool size (F).
**Figure S4**. No correlation between basal plasma TG and VLDL‐TG production was observed (rho =‐0.08, *P* =0.59).
**Figure S5**. The relation between body weight and plasma TG (A), and body weight and plasma insulin (B).

**Table S1**. Summary of results from the mixed modeling analysis.

**Data S1**. The R‐code to reproduce the gene expression analysis.

**Data S2**. Matlab code to reproduce the mixed modeling results.

**Data S3**. Summary of results from gene expression analysis.
