# Male apoE*3‐Leiden.CETP mice on high‐fat high‐cholesterol diet exhibit a biphasic dyslipidemic response, mimicking the changes in plasma lipids observed through life in men

**DOI:** 10.14814/phy2.13608

**Published:** 2018-03-29

**Yared Paalvast, Albert Gerding, Yanan Wang, Vincent W. Bloks, Theo H. van Dijk, Rick Havinga, Ko Willems van Dijk, Patrick C. N. Rensen, Barbara M. Bakker, Jan Albert Kuivenhoven & Albert K. Groen**

Physiol Rep, 5 (19), 2017, e13376, https://doi.org/10.14814/phy2.13376

In Paalvast et al. (2017), the figures 8 and 9 were wrongly processed so that the wrong figures were attached to the figure captions. Both figures 8 and 9 are now the correct figures after a post publication correction so that both figures match the corresponding figure captions.

The authors apologise for the error.
