# Supplementary material for: Male apoE*3‐Leiden.CETP mice on high‐fat high‐cholesterol diet exhibit a biphasic dyslipidemic response, mimicking the changes in plasma lipids observed through life in men
Source: Physiol Rep. 2017 Oct 16;5(19):e13376. doi: 10.14814/phy2.13376 (PMC5641925; doi:10.14814/phy2.13376)
Supplement: Supplementary file 1 — Figure S1. Food intake of E3L.CETP – mice on HFCD diet through time. Figure S2. Liver weight correlates with body weight (ρ = 0.9, P > 0.001). Figure S3. Tracer kinetics of male E3L.CETP mice that were used to measure endogenous glucose production: Mean residence time (A), Bioavailibility (B), AUC (C), apparent volume of distribution (D), metabolic clearance rate (E) and pool size (F). Figure S4. No correlation between basal plasma TG and VLDL‐TG production was observed (rho =‐0.08, P =0.59). Figure S5. The relation between body weight and plasma TG (A), and body weight and plasma insulin (B). [file PHY2-5-e13376-s001.docx]

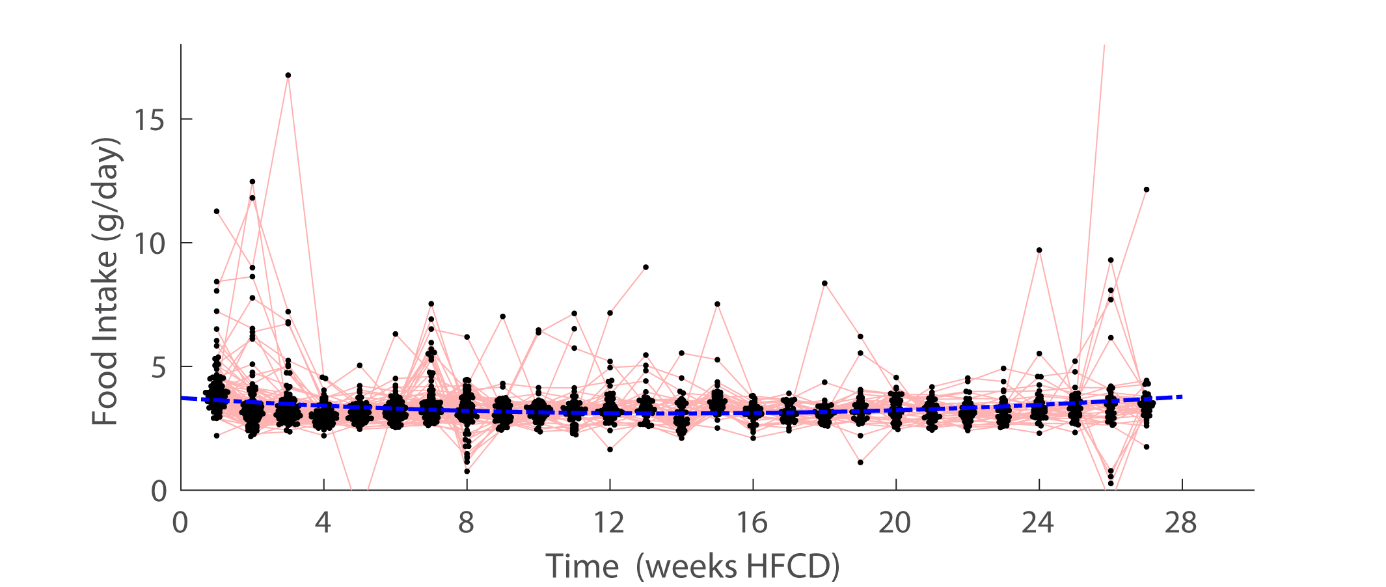


**Figure S1**

Food intake of E3L.CETP – mice on HFCD diet through time. The blue-dotted line represents the group mean response of the mixed model. Red lines connect measurements performed on the same individual animal.


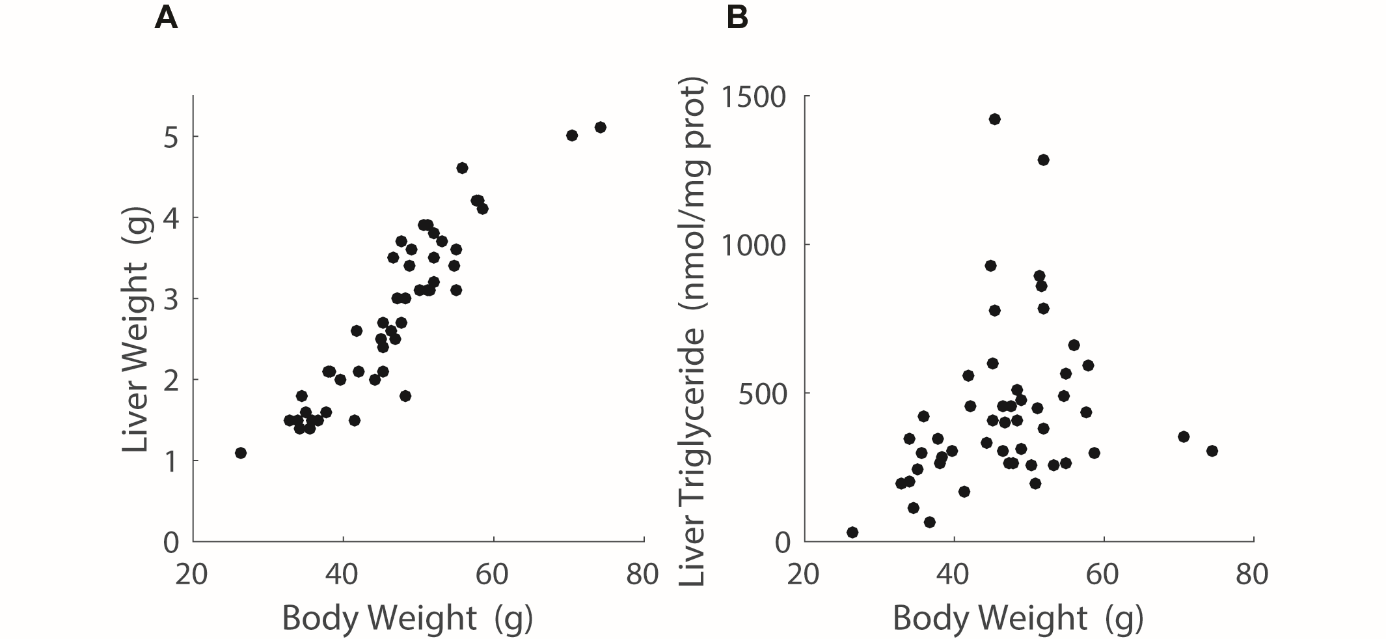


**Figure S2**

Liver weight correlates with body weight (ρ = 0.9, p > 0.001). The correlation between body weight and liver triglyceride concentration is much weaker (ρ = 0.27, p = 0.058).


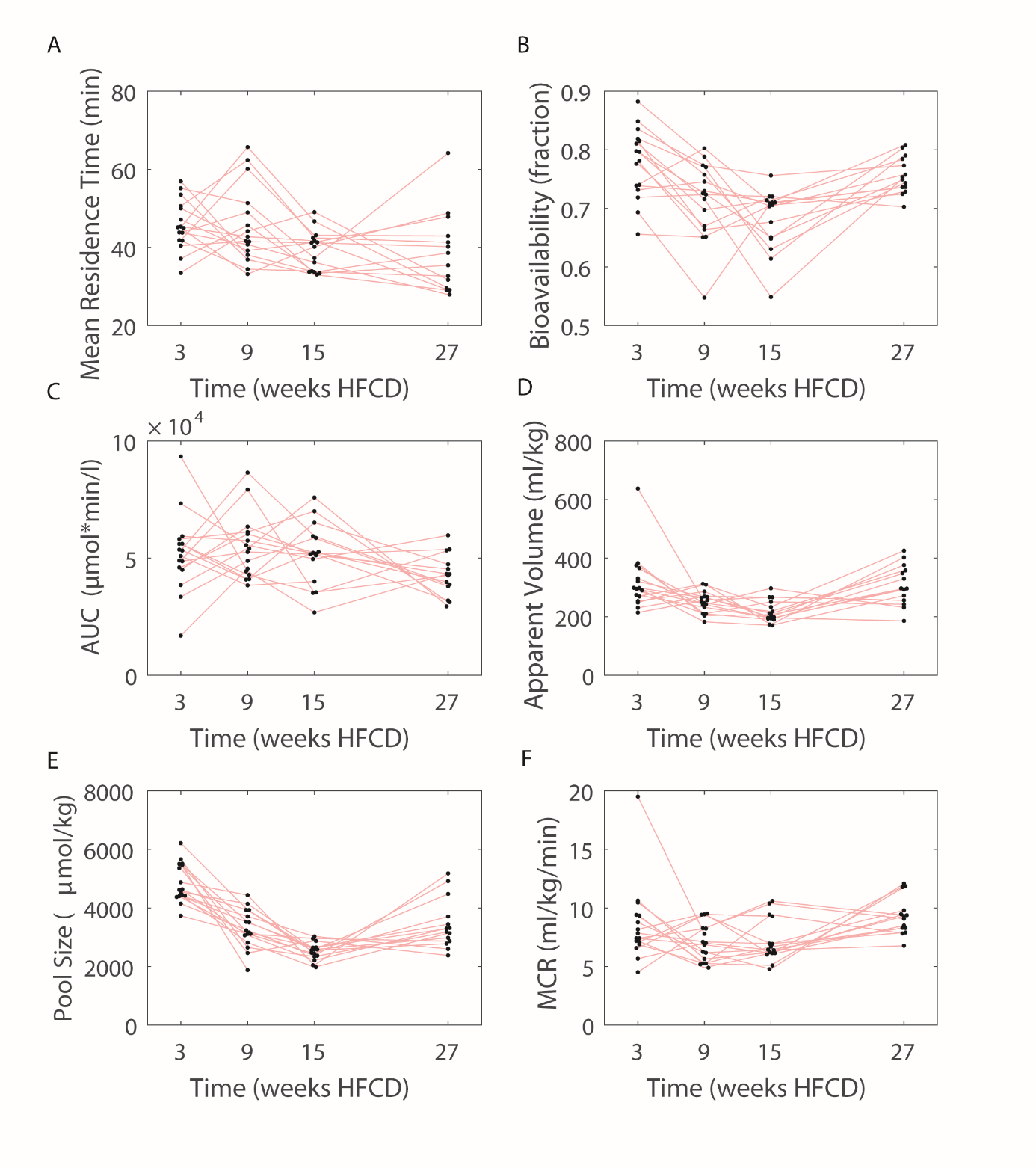


**Figure S3**

Tracer kinetics of male E3L.CETP mice that were used to measure endogenous glucose production: Mean residence time (A), Bioavailibility (B), AUC (C), apparent volume of distribution (D), metabolic clearance rate (E) and pool size (F).


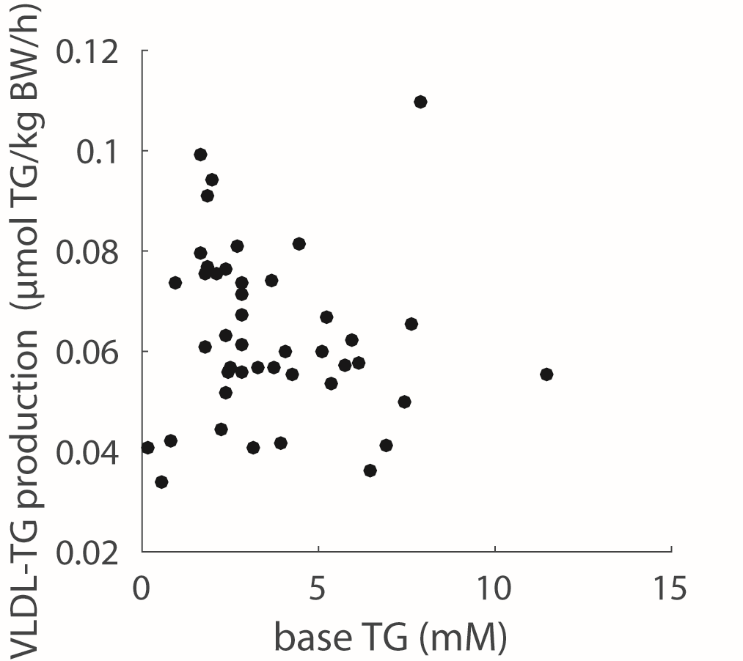


**Figure S4**

No correlation between basal plasma TG and VLDL-TG production was observed (rho =-0.08, p =0.59).


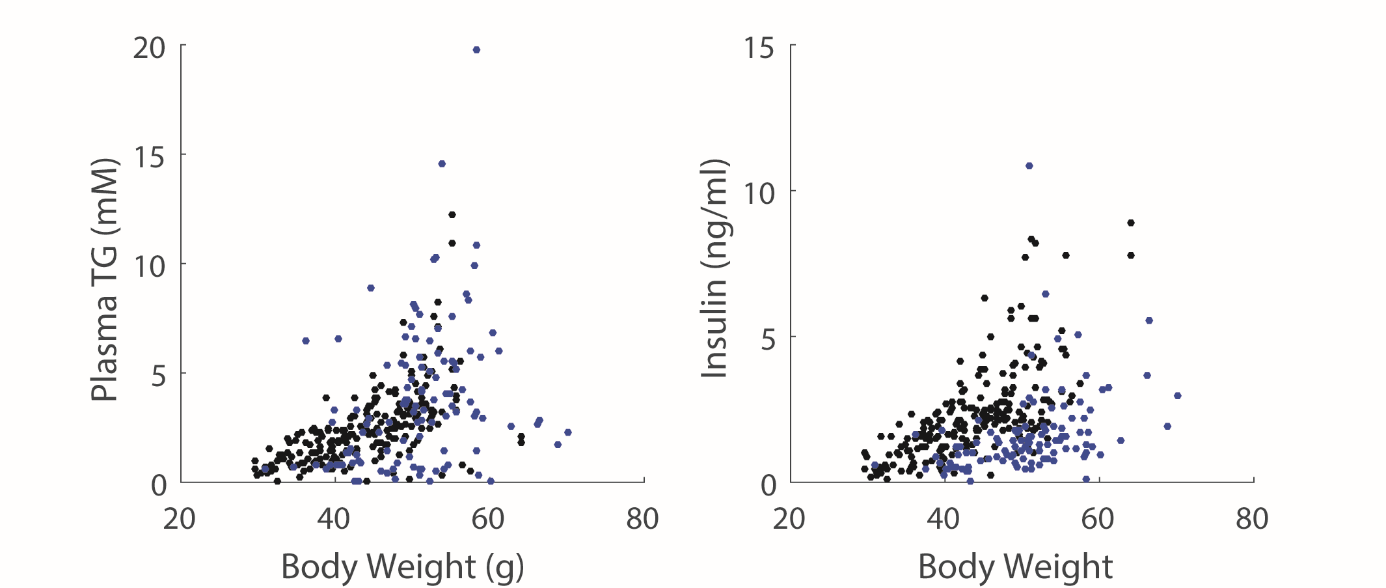


**Figure S5**

The relation between body weight and plasma TG (A), and body weight and plasma insulin (B). Measurements within the first three months (0 – 13 weeks) of HFCD are indicated in black and measurements after three months (17 – 26 weeks) are indicated in blue.
