# Supplementary material for: Male apoE*3‐Leiden.CETP mice on high‐fat high‐cholesterol diet exhibit a biphasic dyslipidemic response, mimicking the changes in plasma lipids observed through life in men
Source: Physiol Rep. 2017 Oct 16;5(19):e13376. doi: 10.14814/phy2.13376 (PMC5641925; doi:10.14814/phy2.13376)

Next Generation Sequencing report

Genome Analysis Facility (GAF), Genomics Coordination Centre (GCC)

University Medical Centre Groningen June 3, 2016

**Report**	
Created on	June 3, 2016
Generated by	MOLGENIS Compute
**Project**	
Project name	1605_Paalvast_Lexogen
Number of samples	
**Contact**	
Name	t.paalvast@umcg.nl
E-mail	t.paalvast@umcg.nl
Pipeline version	NGS_RNA/3.2.3-Molgenis-Compute-v16.05.1-Java-1.8.0_45

Introduction

This report describes a series of statistics about your sequencing data. Together with this report you'll receive fastq files, qc metrics files and geneCount tables. If you, in addition, also want the alignment data, then please notify us via e-mail. In any case we'll delete the raw data, three months after

June 3, 2016

Description of the RNA Isolation, Sample Preparation and sequencing and different steps used in the RNA analysis pipeline

RNA Isolation, Sample Preparation and sequencing
Initial quality check of and RNA quantification of the samples was performed by capillary electrophoresis using the LabChip GX (Perkin Elmer). Non-degraded RNA-samples were selected for subsequent sequencing analysis.
Sequence libraries were generated using the TruSeq RNA sample preparation kits (Illumina) using the Sciclone NGS Liquid Handler (Perkin Elmer). In case of contamination of adapter-duplexes an extra purification of the libraries was performed with the automated agarose gel separation system Labchip XT (PerkinElmer). The obtained cDNA fragment libraries were sequenced on an Illumina HiSeq2500 using default parameters (single read 1x50bp or Paired End 2 x 100 bp) in pools of multiple samples.

Gene expression quantification
The trimmed fastQ files where aligned to build b38 human reference genome using hisat/0.1.5-beta-goolf-1.7.20 [1] allowing for 2 mismatches. Before gene quantification SAMtools/1.2-goolf-1.7.20 [2] was used to sort the aligned reads.
The gene level quantification was performed by HTSeq/0.6.1p1 [3] using --mode=union --stranded=no and, Ensembl version 82 was used as gene annotation database which is included in folder expression/.

Calculate QC metrics on raw and aligned data
Quality control (QC) metrics are calculated for the raw sequencing data. This is done using the tool FastQC FastQC/0.11.3-Java-1.7.0_80 [4]. QC metrics are calculated for the aligned reads using Picard-tools picard/1.130-Java-1.7.0_80 [5] CollectRnaSeqMetrics, MarkDuplicates, CollectInsertSize-Metrics and SAMtools/1.2-goolf-1.7.20 flagstat.

These QC metrics form the basis in this final QC report.

Used toolversions:

Java/1.7.0_80
FastQC/0.11.3-Java-1.7.0_80
hisat/0.1.5-beta-goolf-1.7.20
SAMtools/1.2-goolf-1.7.20
R/3.2.1-goolf-1.7.20
wkhtmltopdf/0.11.0_rc1-static-amd64
picard/1.130-Java-1.7.0_80
HTSeq/0.6.1p1
Python/2.7.9-goolf-1.7.20
GATK/3.4-46-Java-1.7.0_80
Ghostscript/9.16-goolf-1.7.20

1. Dobin A, Davis C a, Schlesinger F, Drenkow J, Zaleski C, Jha S, Batut P, Chaisson M, Gingeras TR: STAR: ultrafast universal RNA-seq aligner. Bioinformatics 2013, 29:15â€“21.
2. Li H, Handsaker B, Wysoker A, Fennell T, Ruan J, Homer N, Marth G, Abecasis G, Durbin R, Subgroup 1000 Genome Project Data Processing: The Sequence Alignment/Map format and SAMtools. Bioinforma 2009, 25 (16):2078â€“2079.
3. Anders S, Pyl PT, Huber W: HTSeq â€“ A Python framework to work with high-throughput sequencing data HTSeq â€“ A Python framework to work with high-throughput sequencing data. 2014:0â€“5.
4. Andrews, S. (2010). FastQC a Quality Control Tool for High Throughput Sequence Data [Online]. Available online at: http://www.bioinformatics.babraham.ac.uk/projects/fastqc/ SAMtools/1.2-goolf-1.7.20
5. Picard Sourceforge Web site. http://picard.sourceforge.net/ picard/1.130-Java-1.7.0_80

Project analysis results

Table 1: Overview statistics

Sample:	M6	M8	M47
## PICARD:ALIGNMENTMETRICS ##			
Bad Cycles	0	0	0
Mean Read Length	39.478387	39.484612	39.486857
Percent PF Reads	1	1	1
Percent PF Reads Aligned	0.93229	0.937269	0.91767
Percent Reads Aligned In Pairs	0	0	0
PF Alinged Bases	85630146	95971005	104005790
PF HQ Aligned Bases	69864197	77446166	85105483
PF HQ Aligned Q20 Bases	69105328	76590988	84174876
PF HQ Aligned Reads	1768916	1960555	2154349
PF HQ Error Rate	0.00083	0.000712	0.000804
PF HQ Median Mismatches	0	0	0
PF Indel Rate	0.000042	0.000038	0.000039
PF Mismatch Rate	0.000807	0.000696	0.000774
PF Noise Reads	0	0	0
PF Reads	2325750	2592593	2869295
PF Reads Aligned	2168273	2429956	2633066
Reads Aligned In Pairs	0	0	0
Strand Balance	0.512756	0.503328	0.508242
Total Reads	2325750	2592593	2869295
## SAMTOOLS:FLAGSTAT ##			
duplicates	1362019	1636208	1806140
mappedReads	2878030	3316329	3475485
totalReads	3035507	3478966	3711714
## PICARD:COLLECTRNASEQMETRICS ##			
Coding Bases	16941300	22132092	23278514
Correct Strand Reads	1589104	1807681	1972473
Ignored Reads	0	0	0
Incorrect Strand Reads	4188	4463	5126
IntergenicB ases	6609574	7339510	7765359
Intronic Bases	6628120	6602857	6748850
Median 3Prime Bias	2.432865	1.963345	2.465865
Median 5Prime Bias	0.0	0.0	0.0
Median 5Prime To 3Prime Bias	0.0	0.0	0.0
Median Cv Coverage	2.669319	2.749523	2.748272
Percent Coding Bases	0.197843	0.230612	0.223819
Percent Correct Strand Reads	0.997371	0.997537	0.997408
Percent Intergenic Bases	0.077187	0.076476	0.074663
Percent Intronic Bases	0.077404	0.068801	0.064889
Percent Mrna Bases	0.839804	0.849841	0.854559
Percent Ribosomal Bases	0.005604	0.004882	0.00589
Percent Usable Bases	0.783218	0.796738	0.784461
Percent Utr Bases	0.641962	0.619229	0.630739
PF Aligned Bases	85630146	95971005	104005790
PF Bases	91816858	102367529	113299440
Ribosomal Bases	479883	468530	612548
Utr Bases	54971276	59428017	65600521
## PICARD:MARKDEDUPMETRICS ##			
Library	SN163_BHT22GBCXX_0712_1_AATGAA	SN163_BHT22GBCXX_0712_1_ACCAGT	SN163_BHT22GBCXX_0712_1_TGTGCA
Percent Duplication	0.628158	0.673349	0.685946
Read Pairs Examined	0	0	0
ReadPairs Duplicates	0	0	0
ReadPairs Optical Duplicates	0	0	0
Unmapped Reads	157477	162637	236229
Unmapped Reads Duplicates	1362019	1636208	1806140
Unpaired Reads Examined	2168273	2429956	2633066

Table 2: Overview statistics

Sample:	M51	M55	M61
## PICARD:ALIGNMENTMETRICS ##			
Bad Cycles	0	0	0
Mean Read Length	39.491221	39.491622	39.488174
Percent PF Reads	1	1	1
Percent PF Reads Aligned	0.923181	0.884297	0.901959
Percent Reads Aligned In Pairs	0	0	0
PF Alinged Bases	110579436	77072397	78179329
PF HQ Aligned Bases	89984997	62424867	64023220
PF HQ Aligned Q20 Bases	89020685	61752180	63295020
PF HQ Aligned Reads	2277724	1580072	1620583
PF HQ Error Rate	0.000703	0.000897	0.000848
PF HQ Median Mismatches	0	0	0
PF Indel Rate	0.000037	0.000037	0.00004
PF Mismatch Rate	0.000678	0.000844	0.000816
PF Noise Reads	0	0	0
PF Reads	3032354	2206252	2194270
PF Reads Aligned	2799413	1950981	1979141
Reads Aligned In Pairs	0	0	0
Strand Balance	0.511407	0.512474	0.519184
Total Reads	3032354	2206252	2194270
## SAMTOOLS:FLAGSTAT ##			
duplicates	1957180	1294152	1318634
mappedReads	3732844	2601395	2605153
totalReads	3965785	2856666	2820282
## PICARD:COLLECTRNASEQMETRICS ##			
Coding Bases	26619421	16494956	17019632
Correct Strand Reads	2108012	1436320	1470608
Ignored Reads	0	0	0
Incorrect Strand Reads	5014	3734	3792
IntergenicB ases	8197916	6339159	6161339
Intronic Bases	7006783	5452161	5249451
Median 3Prime Bias	1.940749	2.770036	2.662585
Median 5Prime Bias	0.0	0.0	0.0
Median 5Prime To 3Prime Bias	0.0	0.0	0.0
Median Cv Coverage	2.738106	2.773775	2.823253
Percent Coding Bases	0.240727	0.214019	0.2177
Percent Correct Strand Reads	0.997627	0.997407	0.997428
Percent Intergenic Bases	0.074136	0.082249	0.07881
Percent Intronic Bases	0.063364	0.070741	0.067146
Percent Mrna Bases	0.85788	0.840109	0.848125
Percent Ribosomal Bases	0.004619	0.006901	0.005918
Percent Usable Bases	0.792174	0.743147	0.765235
Percent Utr Bases	0.617154	0.62609	0.630425
PF Aligned Bases	110579436	77072397	78179329
PF Bases	119751363	87128469	86647715
Ribosomal Bases	510800	531862	462676
Utr Bases	68244518	48254266	49286236
## PICARD:MARKDEDUPMETRICS ##			
Library	SN163_BHT22GBCXX_0712_1_GTCAGG	SN163_BHT22GBCXX_0712_1_ACAGAT	SN163_BHT22GBCXX_0712_1_ACACGC
Percent Duplication	0.699139	0.663334	0.666266
Read Pairs Examined	0	0	0
ReadPairs Duplicates	0	0	0
ReadPairs Optical Duplicates	0	0	0
Unmapped Reads	232941	255271	215129
Unmapped Reads Duplicates	1957180	1294152	1318634
Unpaired Reads Examined	2799413	1950981	1979141

Table 3: Overview statistics

Sample:	M65	M69	M4
## PICARD:ALIGNMENTMETRICS ##			
Bad Cycles	0	0	0
Mean Read Length	39.493493	39.504451	39.486321
Percent PF Reads	1	1	1
Percent PF Reads Aligned	0.933696	0.934795	0.939039
Percent Reads Aligned In Pairs	0	0	0
PF Alinged Bases	116545471	89536181	107122571
PF HQ Aligned Bases	94031265	72948045	87088406
PF HQ Aligned Q20 Bases	93023134	72150630	86158691
PF HQ Aligned Reads	2379961	1845889	2204725
PF HQ Error Rate	0.000698	0.000713	0.000739
PF HQ Median Mismatches	0	0	0
PF Indel Rate	0.000037	0.000033	0.00004
PF Mismatch Rate	0.000679	0.000692	0.000719
PF Noise Reads	0	0	0
PF Reads	3159645	2423879	2888145
PF Reads Aligned	2950147	2265830	2712081
Reads Aligned In Pairs	0	0	0
Strand Balance	0.507264	0.49796	0.511115
Total Reads	3159645	2423879	2888145
## SAMTOOLS:FLAGSTAT ##			
duplicates	2045154	1558094	1779216
mappedReads	3988363	3017569	3612818
totalReads	4197861	3175618	3788882
## PICARD:COLLECTRNASEQMETRICS ##			
Coding Bases	25050969	16989371	20582010
Correct Strand Reads	2193516	1660497	1988794
Ignored Reads	0	0	0
Incorrect Strand Reads	4926	3944	5359
IntergenicB ases	8874607	6775482	8228049
Intronic Bases	7854726	6333026	8333411
Median 3Prime Bias	2.568352	3.398132	2.415349
Median 5Prime Bias	0.0	0.0	0.0
Median 5Prime To 3Prime Bias	0.0	0.0	0.0
Median Cv Coverage	2.755357	2.999692	2.702117
Percent Coding Bases	0.214946	0.189749	0.192135
Percent Correct Strand Reads	0.997759	0.99763	0.997313
Percent Intergenic Bases	0.076147	0.075673	0.07681
Percent Intronic Bases	0.067396	0.070731	0.077793
Percent Mrna Bases	0.850743	0.848152	0.840127
Percent Ribosomal Bases	0.005714	0.005443	0.00527
Percent Usable Bases	0.794566	0.793077	0.789151
Percent Utr Bases	0.635797	0.658404	0.647992
PF Aligned Bases	116545471	89536181	107122571
PF Bases	124785418	95754008	114042221
Ribosomal Bases	665937	487350	564557
Utr Bases	74099240	58950955	69414550
## PICARD:MARKDEDUPMETRICS ##			
Library	SN163_BHT22GBCXX_0712_1_GTGTAG	SN163_BHT22GBCXX_0712_1_GGTATA	SN163_BHT22GBCXX_0712_1_GATTGT
Percent Duplication	0.693238	0.687648	0.656034
Read Pairs Examined	0	0	0
ReadPairs Duplicates	0	0	0
ReadPairs Optical Duplicates	0	0	0
Unmapped Reads	209498	158049	176064
Unmapped Reads Duplicates	2045154	1558094	1779216
Unpaired Reads Examined	2950147	2265830	2712081

Table 4: Overview statistics

Sample:	M11	M15	M17
## PICARD:ALIGNMENTMETRICS ##			
Bad Cycles	0	0	0
Mean Read Length	39.479442	39.473712	39.479608
Percent PF Reads	1	1	1
Percent PF Reads Aligned	0.931349	0.936752	0.931462
Percent Reads Aligned In Pairs	0	0	0
PF Alinged Bases	100586515	54018396	124026049
PF HQ Aligned Bases	82451509	44425695	101291594
PF HQ Aligned Q20 Bases	81539012	43936310	100181939
PF HQ Aligned Reads	2087586	1124909	2564527
PF HQ Error Rate	0.000828	0.000855	0.00082
PF HQ Median Mismatches	0	0	0
PF Indel Rate	0.000041	0.000043	0.000039
PF Mismatch Rate	0.000804	0.000829	0.000797
PF Noise Reads	0	0	0
PF Reads	2734567	1460293	3371396
PF Reads Aligned	2546836	1367933	3140326
Reads Aligned In Pairs	0	0	0
Strand Balance	0.505074	0.507428	0.508233
Total Reads	2734567	1460293	3371396
## SAMTOOLS:FLAGSTAT ##			
duplicates	1646349	789526	2160754
mappedReads	3348184	1798153	4159837
totalReads	3535915	1890513	4390907
## PICARD:COLLECTRNASEQMETRICS ##			
Coding Bases	18265852	9529420	21512536
Correct Strand Reads	1845238	985278	2275104
Ignored Reads	0	0	0
Incorrect Strand Reads	5136	2804	6209
IntergenicB ases	7901783	4366772	9828324
Intronic Bases	8354968	4675617	10110084
Median 3Prime Bias	3.074491	3.3013	3.442177
Median 5Prime Bias	0.0	0.0	0.0
Median 5Prime To 3Prime Bias	0.0	0.0	0.0
Median Cv Coverage	2.717896	2.759506	2.865359
Percent Coding Bases	0.181593	0.176411	0.173452
Percent Correct Strand Reads	0.997224	0.997162	0.997278
Percent Intergenic Bases	0.078557	0.080839	0.079244
Percent Intronic Bases	0.083063	0.086556	0.081516
Percent Mrna Bases	0.830921	0.825236	0.832418
Percent Ribosomal Bases	0.00746	0.00737	0.006822
Percent Usable Bases	0.774176	0.773342	0.775661
Percent Utr Bases	0.649327	0.648825	0.658966
PF Aligned Bases	100586515	54018396	124026049
PF Bases	107959178	57643186	133101393
Ribosomal Bases	750372	398109	846150
Utr Bases	65313548	35048483	81728965
## PICARD:MARKDEDUPMETRICS ##			
Library	SN163_BHT22GBCXX_0712_1_GTGCCA	SN163_BHT22GBCXX_0712_1_ACCGTG	SN163_BHT22GBCXX_0712_1_ACGTCT
Percent Duplication	0.646429	0.577167	0.688067
Read Pairs Examined	0	0	0
ReadPairs Duplicates	0	0	0
ReadPairs Optical Duplicates	0	0	0
Unmapped Reads	187731	92360	231070
Unmapped Reads Duplicates	1646349	789526	2160754
Unpaired Reads Examined	2546836	1367933	3140326

Table 5: Overview statistics

Sample:	M29	M53	M71
## PICARD:ALIGNMENTMETRICS ##			
Bad Cycles	0	0	0
Mean Read Length	39.478905	39.492666	39.48222
Percent PF Reads	1	1	1
Percent PF Reads Aligned	0.931054	0.908071	0.925856
Percent Reads Aligned In Pairs	0	0	0
PF Alinged Bases	70068308	124919334	105087429
PF HQ Aligned Bases	57361905	103079143	85826217
PF HQ Aligned Q20 Bases	56740128	101976631	84895906
PF HQ Aligned Reads	1452378	2609171	2172910
PF HQ Error Rate	0.000804	0.000738	0.000825
PF HQ Median Mismatches	0	0	0
PF Indel Rate	0.000041	0.000037	0.000039
PF Mismatch Rate	0.000784	0.000712	0.000799
PF Noise Reads	0	0	0
PF Reads	1905583	3482340	2873784
PF Reads Aligned	1774201	3162211	2660710
Reads Aligned In Pairs	0	0	0
Strand Balance	0.512959	0.515946	0.509688
Total Reads	1905583	3482340	2873784
## SAMTOOLS:FLAGSTAT ##			
duplicates	1065577	2235763	1751744
mappedReads	2333980	4120258	3511869
totalReads	2465362	4440387	3724943
## PICARD:COLLECTRNASEQMETRICS ##			
Coding Bases	13946656	25972659	20671033
Correct Strand Reads	1296978	2310292	1955531
Ignored Reads	0	0	0
Incorrect Strand Reads	3576	6512	5358
IntergenicB ases	5398483	9770684	8091224
Intronic Bases	5609338	9814802	7832138
Median 3Prime Bias	2.373196	2.667249	2.508595
Median 5Prime Bias	0.0	0.0	0.0
Median 5Prime To 3Prime Bias	0.0	0.0	0.0
Median Cv Coverage	2.610993	2.796956	2.693351
Percent Coding Bases	0.199044	0.207915	0.196703
Percent Correct Strand Reads	0.99725	0.997189	0.997268
Percent Intergenic Bases	0.077046	0.078216	0.076995
Percent Intronic Bases	0.080055	0.078569	0.07453
Percent Mrna Bases	0.835738	0.835807	0.841782
Percent Ribosomal Bases	0.007161	0.007408	0.006694
Percent Usable Bases	0.778393	0.759186	0.779641
Percent Utr Bases	0.636694	0.627892	0.645078
PF Aligned Bases	70068308	124919334	105087429
PF Bases	75230331	137526892	113463372
Ribosomal Bases	501754	925401	703403
Utr Bases	44612080	78435799	67789634
## PICARD:MARKDEDUPMETRICS ##			
Library	SN163_BHT22GBCXX_0712_1_AGACCA	SN163_BHT22GBCXX_0712_1_GTAGAA	SN163_BHT22GBCXX_0712_1_ACTCTT
Percent Duplication	0.600595	0.707025	0.658375
Read Pairs Examined	0	0	0
ReadPairs Duplicates	0	0	0
ReadPairs Optical Duplicates	0	0	0
Unmapped Reads	131382	320129	213074
Unmapped Reads Duplicates	1065577	2235763	1751744
Unpaired Reads Examined	1774201	3162211	2660710

Table 6: Overview statistics

Sample:	M73	M75	M77
## PICARD:ALIGNMENTMETRICS ##			
Bad Cycles	0	0	0
Mean Read Length	39.485559	39.485814	39.487209
Percent PF Reads	1	1	1
Percent PF Reads Aligned	0.926352	0.935725	0.930197
Percent Reads Aligned In Pairs	0	0	0
PF Alinged Bases	84631094	149686932	121893506
PF HQ Aligned Bases	69349129	121918566	99999554
PF HQ Aligned Q20 Bases	68581816	120634185	98937161
PF HQ Aligned Reads	1755633	3086540	2531455
PF HQ Error Rate	0.000794	0.000667	0.000768
PF HQ Median Mismatches	0	0	0
PF Indel Rate	0.000039	0.00004	0.000039
PF Mismatch Rate	0.00077	0.000652	0.000744
PF Noise Reads	0	0	0
PF Reads	2312975	4050096	3317456
PF Reads Aligned	2142628	3789778	3085889
Reads Aligned In Pairs	0	0	0
Strand Balance	0.514152	0.508701	0.511999
Total Reads	2312975	4050096	3317456
## SAMTOOLS:FLAGSTAT ##			
duplicates	1374679	2616178	2108472
mappedReads	2816556	5026842	4056755
totalReads	2986903	5287160	4288322
## PICARD:COLLECTRNASEQMETRICS ##			
Coding Bases	16810854	27727866	23179572
Correct Strand Reads	1570447	2747923	2275370
Ignored Reads	0	0	0
Incorrect Strand Reads	3899	7069	5724
IntergenicB ases	6447760	11765382	9089631
Intronic Bases	6384890	12553860	8952936
Median 3Prime Bias	2.75932	2.683046	2.996271
Median 5Prime Bias	0.0	0.0	0.0
Median 5Prime To 3Prime Bias	0.0	0.0	0.0
Median Cv Coverage	2.719256	2.752642	2.767225
Percent Coding Bases	0.198637	0.185239	0.190162
Percent Correct Strand Reads	0.997523	0.997434	0.997491
Percent Intergenic Bases	0.076187	0.0786	0.07457
Percent Intronic Bases	0.075444	0.083867	0.073449
Percent Mrna Bases	0.840899	0.829877	0.845687
Percent Ribosomal Bases	0.00747	0.007656	0.006294
Percent Usable Bases	0.779228	0.776767	0.786917
Percent Utr Bases	0.642262	0.644638	0.655525
PF Aligned Bases	84631094	149686932	121893506
PF Bases	91329110	159921337	130997077
Ribosomal Bases	632222	1146001	767153
Utr Bases	54355371	96493831	79904223
## PICARD:MARKDEDUPMETRICS ##			
Library	SN163_BHT22GBCXX_0712_1_TTCCGC	SN163_BHT22GBCXX_0712_1_ATAAGA	SN163_BHT22GBCXX_0712_1_AGATAG
Percent Duplication	0.641585	0.690325	0.683262
Read Pairs Examined	0	0	0
ReadPairs Duplicates	0	0	0
ReadPairs Optical Duplicates	0	0	0
Unmapped Reads	170347	260318	231567
Unmapped Reads Duplicates	1374679	2616178	2108472
Unpaired Reads Examined	2142628	3789778	3085889

Table 7: Overview statistics

Sample:	M80	M85	M90
## PICARD:ALIGNMENTMETRICS ##			
Bad Cycles	0	0	0
Mean Read Length	39.486046	39.481786	39.473051
Percent PF Reads	1	1	1
Percent PF Reads Aligned	0.925593	0.926812	0.934717
Percent Reads Aligned In Pairs	0	0	0
PF Alinged Bases	96327738	90781096	54710740
PF HQ Aligned Bases	78987103	74161138	44756696
PF HQ Aligned Q20 Bases	78140612	73342056	44259916
PF HQ Aligned Reads	1999635	1877546	1133267
PF HQ Error Rate	0.000811	0.000803	0.000911
PF HQ Median Mismatches	0	0	0
PF Indel Rate	0.000039	0.000039	0.000038
PF Mismatch Rate	0.000786	0.000775	0.00088
PF Noise Reads	0	0	0
PF Reads	2634779	2479993	1482157
PF Reads Aligned	2438733	2298488	1385397
Reads Aligned In Pairs	0	0	0
Strand Balance	0.511327	0.516496	0.5079
Total Reads	2634779	2479993	1482157
## SAMTOOLS:FLAGSTAT ##			
duplicates	1628711	1481788	800997
mappedReads	3199016	3030011	1833021
totalReads	3395062	3211516	1929781
## PICARD:COLLECTRNASEQMETRICS ##			
Coding Bases	18515359	18897230	9861021
Correct Strand Reads	1803465	1692683	1007522
Ignored Reads	0	0	0
Incorrect Strand Reads	4678	4616	2730
IntergenicB ases	7225531	6920231	4180197
Intronic Bases	6790500	6811971	4372617
Median 3Prime Bias	3.084666	2.344658	3.204934
Median 5Prime Bias	0.0	0.0	0.0
Median 5Prime To 3Prime Bias	0.0	0.0	0.0
Median Cv Coverage	2.759724	2.642707	2.719248
Percent Coding Bases	0.192212	0.208163	0.180239
Percent Correct Strand Reads	0.997413	0.99728	0.997298
Percent Intergenic Bases	0.07501	0.07623	0.076405
Percent Intronic Bases	0.070494	0.075037	0.079922
Percent Mrna Bases	0.84777	0.841681	0.836672
Percent Ribosomal Bases	0.006727	0.007052	0.007
Percent Usable Bases	0.784949	0.780361	0.782407
Percent Utr Bases	0.655558	0.633518	0.656433
PF Aligned Bases	96327738	90781096	54710740
PF Bases	104037004	97914554	58505259
Ribosomal Bases	647954	640196	382975
Utr Bases	63148400	57511473	35913932
## PICARD:MARKDEDUPMETRICS ##			
Library	SN163_BHT22GBCXX_0712_1_ATACTG	SN163_BHT22GBCXX_0712_1_GAGTCC	SN163_BHT22GBCXX_0712_1_GCTCGA
Percent Duplication	0.667851	0.644679	0.578171
Read Pairs Examined	0	0	0
ReadPairs Duplicates	0	0	0
ReadPairs Optical Duplicates	0	0	0
Unmapped Reads	196046	181505	96760
Unmapped Reads Duplicates	1628711	1481788	800997
Unpaired Reads Examined	2438733	2298488	1385397

Table 8: Overview statistics

Sample:	M94	M97	M99
## PICARD:ALIGNMENTMETRICS ##			
Bad Cycles	0	0	0
Mean Read Length	39.479206	39.481067	39.473161
Percent PF Reads	1	1	1
Percent PF Reads Aligned	0.932977	0.938581	0.93632
Percent Reads Aligned In Pairs	0	0	0
PF Alinged Bases	64238516	104208643	132850338
PF HQ Aligned Bases	52511748	85917478	108422492
PF HQ Aligned Q20 Bases	51948485	85002045	107230523
PF HQ Aligned Reads	1329506	2175251	2745427
PF HQ Error Rate	0.000823	0.000756	0.000875
PF HQ Median Mismatches	0	0	0
PF Indel Rate	0.000038	0.000039	0.00004
PF Mismatch Rate	0.0008	0.000738	0.000848
PF Noise Reads	0	0	0
PF Reads	1743377	2811214	3592976
PF Reads Aligned	1626530	2638553	3364177
Reads Aligned In Pairs	0	0	0
Strand Balance	0.511285	0.508143	0.508743
Total Reads	1743377	2811214	3592976
## SAMTOOLS:FLAGSTAT ##			
duplicates	971358	1760316	2291619
mappedReads	2149338	3448722	4467881
totalReads	2266185	3621383	4696680
## PICARD:COLLECTRNASEQMETRICS ##			
Coding Bases	11987794	20066893	22744582
Correct Strand Reads	1188937	1936577	2458109
Ignored Reads	0	0	0
Incorrect Strand Reads	3163	5028	6276
IntergenicB ases	4891263	7627706	10261742
Intronic Bases	5047795	7963386	9870827
Median 3Prime Bias	2.790428	2.851007	3.47416
Median 5Prime Bias	0.0	0.0	0.0
Median 5Prime To 3Prime Bias	0.0	0.0	0.0
Median Cv Coverage	2.685563	2.765275	2.712416
Percent Coding Bases	0.186614	0.192565	0.171205
Percent Correct Strand Reads	0.997347	0.99741	0.997453
Percent Intergenic Bases	0.076142	0.073196	0.077243
Percent Intronic Bases	0.078579	0.076418	0.0743
Percent Mrna Bases	0.838495	0.843243	0.843083
Percent Ribosomal Bases	0.006784	0.007143	0.005374
Percent Usable Bases	0.782593	0.791724	0.789727
Percent Utr Bases	0.651881	0.650678	0.671879
PF Aligned Bases	64238516	104208643	132850338
PF Bases	68827139	110989728	141826120
Ribosomal Bases	435810	744352	713901
Utr Bases	41875855	67806312	89259291
## PICARD:MARKDEDUPMETRICS ##			
Library	SN163_BHT22GBCXX_0712_1_AGTACT	SN163_BHT22GBCXX_0712_1_TCAGGA	SN163_BHT22GBCXX_0712_1_TAGGCT
Percent Duplication	0.597196	0.667152	0.681183
Read Pairs Examined	0	0	0
ReadPairs Duplicates	0	0	0
ReadPairs Optical Duplicates	0	0	0
Unmapped Reads	116847	172661	228799
Unmapped Reads Duplicates	971358	1760316	2291619
Unpaired Reads Examined	1626530	2638553	3364177

Table 9: Overview statistics

Sample:	M101	M103	M105
## PICARD:ALIGNMENTMETRICS ##			
Bad Cycles	0	0	0
Mean Read Length	39.48686	39.471293	39.477248
Percent PF Reads	1	1	1
Percent PF Reads Aligned	0.905818	0.90748	0.85019
Percent Reads Aligned In Pairs	0	0	0
PF Alinged Bases	105759148	61822185	87369985
PF HQ Aligned Bases	88065140	51587920	72451571
PF HQ Aligned Q20 Bases	87018354	51022240	71667595
PF HQ Aligned Reads	2229101	1306251	1834129
PF HQ Error Rate	0.000943	0.001062	0.001206
PF HQ Median Mismatches	0	0	0
PF Indel Rate	0.000043	0.000043	0.000044
PF Mismatch Rate	0.000923	0.001034	0.00116
PF Noise Reads	0	0	0
PF Reads	2955407	1725004	2601427
PF Reads Aligned	2677060	1565407	2211706
Reads Aligned In Pairs	0	0	0
Strand Balance	0.522583	0.523204	0.527209
Total Reads	2955407	1725004	2601427
## SAMTOOLS:FLAGSTAT ##			
duplicates	1660323	868740	1415119
mappedReads	3421282	2003946	2850848
totalReads	3699629	2163543	3240569
## PICARD:COLLECTRNASEQMETRICS ##			
Coding Bases	16727128	9471306	14084344
Correct Strand Reads	1802915	1039002	1482641
Ignored Reads	0	0	0
Incorrect Strand Reads	5590	3636	4841
IntergenicB ases	8388871	4993666	7230920
Intronic Bases	12159681	7614252	9365871
Median 3Prime Bias	3.277904	3.72961	4.040278
Median 5Prime Bias	0.0	0.0	0.0
Median 5Prime To 3Prime Bias	0.0	0.0	0.0
Median Cv Coverage	2.750588	2.827539	2.838979
Percent Coding Bases	0.158162	0.153202	0.161203
Percent Correct Strand Reads	0.996909	0.996513	0.996746
Percent Intergenic Bases	0.079321	0.080775	0.082762
Percent Intronic Bases	0.114975	0.123164	0.107198
Percent Mrna Bases	0.772118	0.763119	0.767356
Percent Ribosomal Bases	0.033587	0.032943	0.042685
Percent Usable Bases	0.699732	0.692891	0.652831
Percent Utr Bases	0.613956	0.609916	0.606153
PF Aligned Bases	105759148	61822185	87369985
PF Bases	116699741	68088138	102697178
Ribosomal Bases	3552097	2036632	3729384
Utr Bases	64931447	37706370	52959549
## PICARD:MARKDEDUPMETRICS ##			
Library	SN163_BHT22GBCXX_0712_1_AGGCAT	SN163_BHT22GBCXX_0712_1_GAAGTG	SN163_BHT22GBCXX_0712_1_AACAAG
Percent Duplication	0.620204	0.554961	0.639831
Read Pairs Examined	0	0	0
ReadPairs Duplicates	0	0	0
ReadPairs Optical Duplicates	0	0	0
Unmapped Reads	278347	159597	389721
Unmapped Reads Duplicates	1660323	868740	1415119
Unpaired Reads Examined	2677060	1565407	2211706

Table 10: Overview statistics

Sample:	M111	M113	M115
## PICARD:ALIGNMENTMETRICS ##			
Bad Cycles	0	0	0
Mean Read Length	39.467348	39.472348	39.470142
Percent PF Reads	1	1	1
Percent PF Reads Aligned	0.83472	0.844879	0.852353
Percent Reads Aligned In Pairs	0	0	0
PF Alinged Bases	71172605	67682387	79256315
PF HQ Aligned Bases	58950005	55970747	65596840
PF HQ Aligned Q20 Bases	58302565	55349434	64873804
PF HQ Aligned Reads	1492518	1416851	1660821
PF HQ Error Rate	0.00138	0.001211	0.001272
PF HQ Median Mismatches	0	0	0
PF Indel Rate	0.000043	0.000043	0.000046
PF Mismatch Rate	0.00132	0.001168	0.001229
PF Noise Reads	0	0	0
PF Reads	2158668	2027867	2354142
PF Reads Aligned	1801883	1713303	2006560
Reads Aligned In Pairs	0	0	0
Strand Balance	0.529799	0.536301	0.529377
Total Reads	2158668	2027867	2354142
## SAMTOOLS:FLAGSTAT ##			
duplicates	1108466	1047301	1238578
mappedReads	2326518	2191832	2584711
totalReads	2683303	2506396	2932293
## PICARD:COLLECTRNASEQMETRICS ##			
Coding Bases	11648987	11535009	12875997
Correct Strand Reads	1191676	1134484	1330517
Ignored Reads	0	0	0
Incorrect Strand Reads	4090	3732	4457
IntergenicB ases	6017047	5724934	6580746
Intronic Bases	8124753	7394539	8623490
Median 3Prime Bias	3.844815	3.286836	3.941747
Median 5Prime Bias	0.0	0.0	0.0
Median 5Prime To 3Prime Bias	0.0	0.0	0.0
Median Cv Coverage	2.809651	2.783852	2.769022
Percent Coding Bases	0.163672	0.170429	0.16246
Percent Correct Strand Reads	0.99658	0.996721	0.996661
Percent Intergenic Bases	0.084542	0.084585	0.083031
Percent Intronic Bases	0.114156	0.109254	0.108805
Percent Mrna Bases	0.756228	0.756263	0.759081
Percent Ribosomal Bases	0.045075	0.0499	0.049084
Percent Usable Bases	0.631745	0.639464	0.647472
Percent Utr Bases	0.592556	0.585834	0.596621
PF Aligned Bases	71172605	67682387	79256315
PF Bases	85196902	80044672	92918319
Ribosomal Bases	3208129	3377367	3890208
Utr Bases	42173761	39650654	47285975
## PICARD:MARKDEDUPMETRICS ##			
Library	SN163_BHT22GBCXX_0712_1_TTGGTA	SN163_BHT22GBCXX_0712_1_ACCTAC	SN163_BHT22GBCXX_0712_1_AGAATC
Percent Duplication	0.615171	0.611276	0.617264
Read Pairs Examined	0	0	0
ReadPairs Duplicates	0	0	0
ReadPairs Optical Duplicates	0	0	0
Unmapped Reads	356785	314564	347582
Unmapped Reads Duplicates	1108466	1047301	1238578
Unpaired Reads Examined	1801883	1713303	2006560

Table 11: Overview statistics

Sample:	M117	M121	M2
## PICARD:ALIGNMENTMETRICS ##			
Bad Cycles	0	0	0
Mean Read Length	39.459062	39.456325	39.481318
Percent PF Reads	1	1	1
Percent PF Reads Aligned	0.842008	0.832355	0.913696
Percent Reads Aligned In Pairs	0	0	0
PF Alinged Bases	36247673	69493706	127344253
PF HQ Aligned Bases	30092551	57741807	106126527
PF HQ Aligned Q20 Bases	29751464	57087733	104968137
PF HQ Aligned Reads	761919	1462067	2686722
PF HQ Error Rate	0.001384	0.001496	0.000961
PF HQ Median Mismatches	0	0	0
PF Indel Rate	0.000043	0.000042	0.000046
PF Mismatch Rate	0.001347	0.001454	0.000942
PF Noise Reads	0	0	0
PF Reads	1089939	2113959	3528462
PF Reads Aligned	917737	1759565	3223941
Reads Aligned In Pairs	0	0	0
Strand Balance	0.533473	0.530675	0.519046
Total Reads	1089939	2113959	3528462
## SAMTOOLS:FLAGSTAT ##			
duplicates	493637	1084849	2042427
mappedReads	1168183	2256700	4123865
totalReads	1340385	2611094	4428386
## PICARD:COLLECTRNASEQMETRICS ##			
Coding Bases	5538907	10678909	19098843
Correct Strand Reads	604053	1152790	2170174
Ignored Reads	0	0	0
Incorrect Strand Reads	1949	4154	7203
IntergenicB ases	3062714	5862363	10187999
Intronic Bases	3780533	7657126	14835197
Median 3Prime Bias	4.363448	4.529737	3.558498
Median 5Prime Bias	0.0	0.0	0.0
Median 5Prime To 3Prime Bias	0.0	0.0	0.0
Median Cv Coverage	2.831264	2.933133	2.790279
Percent Coding Bases	0.152807	0.153667	0.149978
Percent Correct Strand Reads	0.996784	0.99641	0.996692
Percent Intergenic Bases	0.084494	0.084358	0.080004
Percent Intronic Bases	0.104297	0.110184	0.116497
Percent Mrna Bases	0.755989	0.752663	0.772602
Percent Ribosomal Bases	0.055221	0.052796	0.030898
Percent Usable Bases	0.637158	0.627094	0.706249
Percent Utr Bases	0.603182	0.598996	0.622624
PF Aligned Bases	36247673	69493706	127344253
PF Bases	43007971	83409053	139308329
Ribosomal Bases	2001627	3668980	3934705
Utr Bases	21863952	41626418	79287584
## PICARD:MARKDEDUPMETRICS ##			
Library	SN163_BHT22GBCXX_0712_1_AACCGA	SN163_BHT22GBCXX_0712_1_CAACAG	SN163_BHT22GBCXX_0712_1_TGGATT
Percent Duplication	0.537885	0.616544	0.633519
Read Pairs Examined	0	0	0
ReadPairs Duplicates	0	0	0
ReadPairs Optical Duplicates	0	0	0
Unmapped Reads	172202	354394	304521
Unmapped Reads Duplicates	493637	1084849	2042427
Unpaired Reads Examined	917737	1759565	3223941

Table 12: Overview statistics

Sample:	M13	M20	M24
## PICARD:ALIGNMENTMETRICS ##			
Bad Cycles	0	0	0
Mean Read Length	39.476139	39.465239	39.475607
Percent PF Reads	1	1	1
Percent PF Reads Aligned	0.888708	0.875919	0.893133
Percent Reads Aligned In Pairs	0	0	0
PF Alinged Bases	91703691	43857239	89303734
PF HQ Aligned Bases	76284424	36484674	74123720
PF HQ Aligned Q20 Bases	75439636	36073642	73291354
PF HQ Aligned Reads	1931252	923827	1876589
PF HQ Error Rate	0.001113	0.001215	0.00109
PF HQ Median Mismatches	0	0	0
PF Indel Rate	0.000045	0.000046	0.000044
PF Mismatch Rate	0.00109	0.001187	0.001068
PF Noise Reads	0	0	0
PF Reads	2612407	1267840	2531487
PF Reads Aligned	2321666	1110525	2260955
Reads Aligned In Pairs	0	0	0
Strand Balance	0.523149	0.525153	0.523072
Total Reads	2612407	1267840	2531487
## SAMTOOLS:FLAGSTAT ##			
duplicates	1353079	571044	1310720
mappedReads	2966395	1418892	2904335
totalReads	3257136	1576207	3174867
## PICARD:COLLECTRNASEQMETRICS ##			
Coding Bases	14354827	6536107	13989186
Correct Strand Reads	1526704	727651	1480306
Ignored Reads	0	0	0
Incorrect Strand Reads	5526	2528	5390
IntergenicB ases	7828386	3634188	7411579
Intronic Bases	11467862	5297548	11118357
Median 3Prime Bias	3.488368	4.046486	3.690924
Median 5Prime Bias	0.0	0.0	0.0
Median 5Prime To 3Prime Bias	0.0	0.0	0.0
Median Cv Coverage	2.663911	2.75275	2.590834
Percent Coding Bases	0.156535	0.149031	0.156647
Percent Correct Strand Reads	0.996393	0.996538	0.996372
Percent Intergenic Bases	0.085366	0.082864	0.082993
Percent Intronic Bases	0.125053	0.120791	0.1245
Percent Mrna Bases	0.751882	0.752905	0.752219
Percent Ribosomal Bases	0.0377	0.043442	0.040289
Percent Usable Bases	0.668591	0.659936	0.672217
Percent Utr Bases	0.595347	0.603873	0.595571
PF Aligned Bases	91703691	43857239	89303734
PF Bases	103127743	50035609	99931986
Ribosomal Bases	3457224	1905231	3597950
Utr Bases	54595487	26484213	53186757
## PICARD:MARKDEDUPMETRICS ##			
Library	SN163_BHT22GBCXX_0712_1_GCGAAT	SN163_BHT22GBCXX_0712_1_TGGCGA	SN163_BHT22GBCXX_0712_1_CAATGC
Percent Duplication	0.582805	0.514211	0.57972
Read Pairs Examined	0	0	0
ReadPairs Duplicates	0	0	0
ReadPairs Optical Duplicates	0	0	0
Unmapped Reads	290741	157315	270532
Unmapped Reads Duplicates	1353079	571044	1310720
Unpaired Reads Examined	2321666	1110525	2260955

Table 13: Overview statistics

Sample:	M26	M49	M57
## PICARD:ALIGNMENTMETRICS ##			
Bad Cycles	0	0	0
Mean Read Length	39.445226	39.464546	39.464192
Percent PF Reads	1	1	1
Percent PF Reads Aligned	0.826532	0.844281	0.872828
Percent Reads Aligned In Pairs	0	0	0
PF Alinged Bases	72639055	67624641	95922193
PF HQ Aligned Bases	59896326	55776183	79946416
PF HQ Aligned Q20 Bases	59193987	55147003	79051408
PF HQ Aligned Reads	1517078	1412189	2024376
PF HQ Error Rate	0.001817	0.001439	0.001287
PF HQ Median Mismatches	0	0	0
PF Indel Rate	0.000044	0.000042	0.000046
PF Mismatch Rate	0.001754	0.001389	0.001253
PF Noise Reads	0	0	0
PF Reads	2225827	2027906	2782769
PF Reads Aligned	1839718	1712122	2428879
Reads Aligned In Pairs	0	0	0
Strand Balance	0.525666	0.52532	0.523567
Total Reads	2225827	2027906	2782769
## SAMTOOLS:FLAGSTAT ##			
duplicates	1092218	1018750	1473585
mappedReads	2375537	2200383	3108129
totalReads	2761646	2516167	3462019
## PICARD:COLLECTRNASEQMETRICS ##			
Coding Bases	11770429	11227652	15536605
Correct Strand Reads	1213264	1149976	1591671
Ignored Reads	0	0	0
Incorrect Strand Reads	4345	4450	5581
IntergenicB ases	6203840	5651048	7941206
Intronic Bases	7703667	6872419	11639313
Median 3Prime Bias	4.372382	4.103014	3.810554
Median 5Prime Bias	0.0	0.0	0.0
Median 5Prime To 3Prime Bias	0.0	0.0	0.0
Median Cv Coverage	2.635379	2.623375	2.658529
Percent Coding Bases	0.16204	0.166029	0.161971
Percent Correct Strand Reads	0.996432	0.996145	0.996506
Percent Intergenic Bases	0.085406	0.083565	0.082788
Percent Intronic Bases	0.106054	0.101626	0.121341
Percent Mrna Bases	0.755091	0.766908	0.750372
Percent Ribosomal Bases	0.05345	0.047903	0.0455
Percent Usable Bases	0.624717	0.648027	0.655414
Percent Utr Bases	0.593051	0.600879	0.588401
PF Aligned Bases	72639055	67624641	95922193
PF Bases	87798249	80030389	109819730
Ribosomal Bases	3882564	3239412	4364449
Utr Bases	43078644	40634204	56440738
## PICARD:MARKDEDUPMETRICS ##			
Library	SN163_BHT22GBCXX_0712_1_GCAGCC	SN163_BHT22GBCXX_0712_1_CGATCT	SN163_BHT22GBCXX_0712_1_CACTAA
Percent Duplication	0.593688	0.595022	0.606693
Read Pairs Examined	0	0	0
ReadPairs Duplicates	0	0	0
ReadPairs Optical Duplicates	0	0	0
Unmapped Reads	386109	315784	353890
Unmapped Reads Duplicates	1092218	1018750	1473585
Unpaired Reads Examined	1839718	1712122	2428879

Table 14: Overview statistics

Sample:	M59	M63	M67
## PICARD:ALIGNMENTMETRICS ##			
Bad Cycles	0	0	0
Mean Read Length	39.471586	39.464332	39.466892
Percent PF Reads	1	1	1
Percent PF Reads Aligned	0.853384	0.842327	0.853985
Percent Reads Aligned In Pairs	0	0	0
PF Alinged Bases	65271239	61633324	64382161
PF HQ Aligned Bases	54219245	51544588	53336595
PF HQ Aligned Q20 Bases	53611834	50951047	52736119
PF HQ Aligned Reads	1372599	1304919	1350366
PF HQ Error Rate	0.001231	0.001261	0.001247
PF HQ Median Mismatches	0	0	0
PF Indel Rate	0.000043	0.000042	0.000043
PF Mismatch Rate	0.001187	0.001227	0.001212
PF Noise Reads	0	0	0
PF Reads	1936282	1852370	1908713
PF Reads Aligned	1652392	1560301	1630012
Reads Aligned In Pairs	0	0	0
Strand Balance	0.527677	0.533093	0.522187
Total Reads	1936282	1852370	1908713
## SAMTOOLS:FLAGSTAT ##			
duplicates	990955	937369	922299
mappedReads	2116251	1975944	2093437
totalReads	2400141	2268013	2372138
## PICARD:COLLECTRNASEQMETRICS ##			
Coding Bases	10812531	10387913	9745316
Correct Strand Reads	1106602	1038732	1069784
Ignored Reads	0	0	0
Incorrect Strand Reads	3822	3780	3712
IntergenicB ases	5429950	5031455	5337584
Intronic Bases	7060203	6612176	7476594
Median 3Prime Bias	3.955686	4.032913	4.088169
Median 5Prime Bias	0.0	0.0	0.0
Median 5Prime To 3Prime Bias	0.0	0.0	0.0
Median Cv Coverage	2.771911	2.806835	2.741626
Percent Coding Bases	0.165655	0.168544	0.151367
Percent Correct Strand Reads	0.996558	0.996374	0.996542
Percent Intergenic Bases	0.083191	0.081635	0.082905
Percent Intronic Bases	0.108167	0.107282	0.116128
Percent Mrna Bases	0.764339	0.762747	0.754991
Percent Ribosomal Bases	0.044305	0.048336	0.045977
Percent Usable Bases	0.652762	0.643078	0.645259
Percent Utr Bases	0.598684	0.594203	0.603624
PF Aligned Bases	65271239	61633324	64382161
PF Bases	76428121	73102544	75330969
Ribosomal Bases	2891810	2979118	2960128
Utr Bases	39076828	36622727	38862639
## PICARD:MARKDEDUPMETRICS ##			
Library	SN163_BHT22GBCXX_0712_1_GGAGGT	SN163_BHT22GBCXX_0712_1_CGCCTG	SN163_BHT22GBCXX_0712_1_CATCTA
Percent Duplication	0.599709	0.600762	0.565823
Read Pairs Examined	0	0	0
ReadPairs Duplicates	0	0	0
ReadPairs Optical Duplicates	0	0	0
Unmapped Reads	283890	292069	278701
Unmapped Reads Duplicates	990955	937369	922299
Unpaired Reads Examined	1652392	1560301	1630012

Table 15: Overview statistics

Sample:	M83	M87	M92
## PICARD:ALIGNMENTMETRICS ##			
Bad Cycles	0	0	0
Mean Read Length	39.468073	39.471422	39.469227
Percent PF Reads	1	1	1
Percent PF Reads Aligned	0.845788	0.877275	0.889654
Percent Reads Aligned In Pairs	0	0	0
PF Alinged Bases	63328548	98156226	72850732
PF HQ Aligned Bases	52039847	81984473	60399693
PF HQ Aligned Q20 Bases	51460566	81079293	59705683
PF HQ Aligned Reads	1317559	2075695	1529324
PF HQ Error Rate	0.00125	0.001106	0.001087
PF HQ Median Mismatches	0	0	0
PF Indel Rate	0.000045	0.000044	0.000044
PF Mismatch Rate	0.001209	0.001081	0.001058
PF Noise Reads	0	0	0
PF Reads	1895699	2832759	2073441
PF Reads Aligned	1603359	2485108	1844645
Reads Aligned In Pairs	0	0	0
Strand Balance	0.527081	0.521329	0.526082
Total Reads	1895699	2832759	2073441
## SAMTOOLS:FLAGSTAT ##			
duplicates	925321	1482396	1038312
mappedReads	2075003	3159507	2371854
totalReads	2367343	3507158	2600650
## PICARD:COLLECTRNASEQMETRICS ##			
Coding Bases	10628097	15903973	12263347
Correct Strand Reads	1057373	1630592	1212303
Ignored Reads	0	0	0
Incorrect Strand Reads	3869	5338	4201
IntergenicB ases	5539945	7913020	6199606
Intronic Bases	6820233	11593588	8634134
Median 3Prime Bias	3.709091	3.429195	3.008384
Median 5Prime Bias	0.0	0.0	0.0
Median 5Prime To 3Prime Bias	0.0	0.0	0.0
Median Cv Coverage	2.658601	2.542269	2.602863
Percent Coding Bases	0.167825	0.162027	0.168335
Percent Correct Strand Reads	0.996354	0.996737	0.996547
Percent Intergenic Bases	0.087479	0.080617	0.0851
Percent Intronic Bases	0.107696	0.118114	0.118518
Percent Mrna Bases	0.75483	0.75687	0.754697
Percent Ribosomal Bases	0.049996	0.044401	0.041686
Percent Usable Bases	0.638901	0.664427	0.671825
Percent Utr Bases	0.587005	0.594843	0.586361
PF Aligned Bases	63328548	98156226	72850732
PF Bases	74819586	111813026	81837113
Ribosomal Bases	3166186	4358199	3036866
Utr Bases	37174184	58387568	42716855
## PICARD:MARKDEDUPMETRICS ##			
Library	SN163_BHT22GBCXX_0712_1_AAGCTC	SN163_BHT22GBCXX_0712_1_CAGGAC	SN163_BHT22GBCXX_0712_1_CCGACC
Percent Duplication	0.577114	0.596512	0.562879
Read Pairs Examined	0	0	0
ReadPairs Duplicates	0	0	0
ReadPairs Optical Duplicates	0	0	0
Unmapped Reads	292340	347651	228796
Unmapped Reads Duplicates	925321	1482396	1038312
Unpaired Reads Examined	1603359	2485108	1844645

Table 16: Overview statistics

Sample:	M107	M109	M119
## PICARD:ALIGNMENTMETRICS ##			
Bad Cycles	0	0	0
Mean Read Length	39.474015	39.473029	39.477244
Percent PF Reads	1	1	1
Percent PF Reads Aligned	0.855587	0.863067	0.874004
Percent Reads Aligned In Pairs	0	0	0
PF Alinged Bases	64210669	67405307	90838646
PF HQ Aligned Bases	52920929	55820911	75726969
PF HQ Aligned Q20 Bases	52338798	55210178	74872111
PF HQ Aligned Reads	1339732	1413230	1917044
PF HQ Error Rate	0.001213	0.001176	0.001065
PF HQ Median Mismatches	0	0	0
PF Indel Rate	0.000043	0.000044	0.000042
PF Mismatch Rate	0.001157	0.001133	0.001038
PF Noise Reads	0	0	0
PF Reads	1899922	1977233	2631157
PF Reads Aligned	1625549	1706485	2299641
Reads Aligned In Pairs	0	0	0
Strand Balance	0.526868	0.524493	0.527818
Total Reads	1899922	1977233	2631157
## SAMTOOLS:FLAGSTAT ##			
duplicates	939129	994532	1410773
mappedReads	2100511	2187412	2923939
totalReads	2374884	2458160	3255455
## PICARD:COLLECTRNASEQMETRICS ##			
Coding Bases	12106369	11827412	15827206
Correct Strand Reads	1083056	1140489	1552191
Ignored Reads	0	0	0
Incorrect Strand Reads	3537	3678	4973
IntergenicB ases	5431143	5582695	7228032
Intronic Bases	6939998	7199455	9532187
Median 3Prime Bias	2.956834	3.27714	3.296964
Median 5Prime Bias	0.0	0.0	0.0
Median 5Prime To 3Prime Bias	0.0	0.0	0.0
Median Cv Coverage	2.54345	2.600172	2.581008
Percent Coding Bases	0.188541	0.175467	0.174234
Percent Correct Strand Reads	0.996745	0.996785	0.996806
Percent Intergenic Bases	0.084583	0.082823	0.07957
Percent Intronic Bases	0.108082	0.106808	0.104935
Percent Mrna Bases	0.761092	0.765067	0.773146
Percent Ribosomal Bases	0.046244	0.045303	0.04235
Percent Usable Bases	0.651624	0.660747	0.676143
Percent Utr Bases	0.57255	0.589599	0.598912
PF Aligned Bases	64210669	67405307	90838646
PF Bases	74997549	78047375	103870827
Ribosomal Bases	2969381	3053683	3847011
Utr Bases	36763844	39742134	54404327
## PICARD:MARKDEDUPMETRICS ##			
Library	SN163_BHT22GBCXX_0712_1_AAGTGG	SN163_BHT22GBCXX_0712_1_TACCTT	SN163_BHT22GBCXX_0712_1_GGCCAA
Percent Duplication	0.57773	0.582796	0.613475
Read Pairs Examined	0	0	0
ReadPairs Duplicates	0	0	0
ReadPairs Optical Duplicates	0	0	0
Unmapped Reads	274373	270748	331516
Unmapped Reads Duplicates	939129	994532	1410773
Unpaired Reads Examined	1625549	1706485	2299641

Distribution of GC percentage

The following figures show the GC distribution per sample.

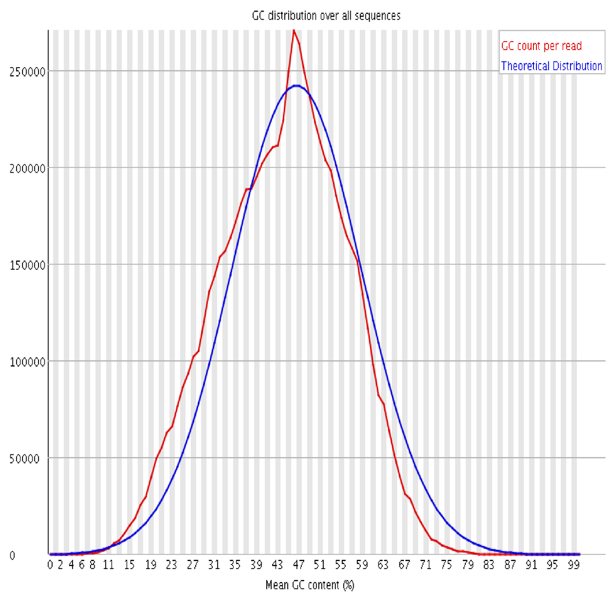

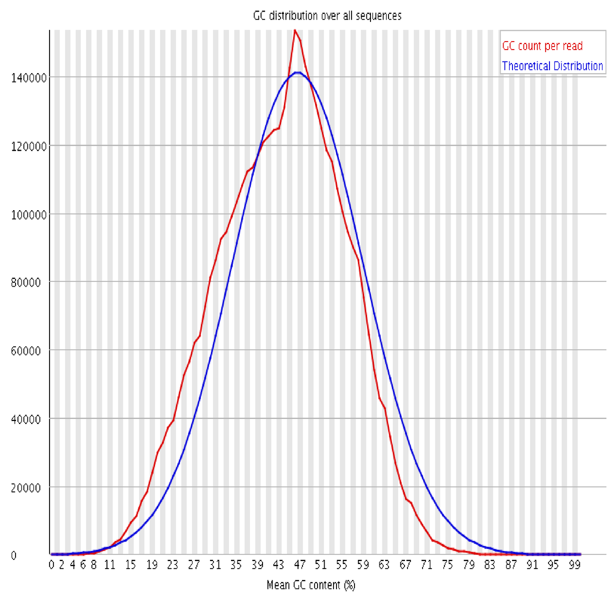

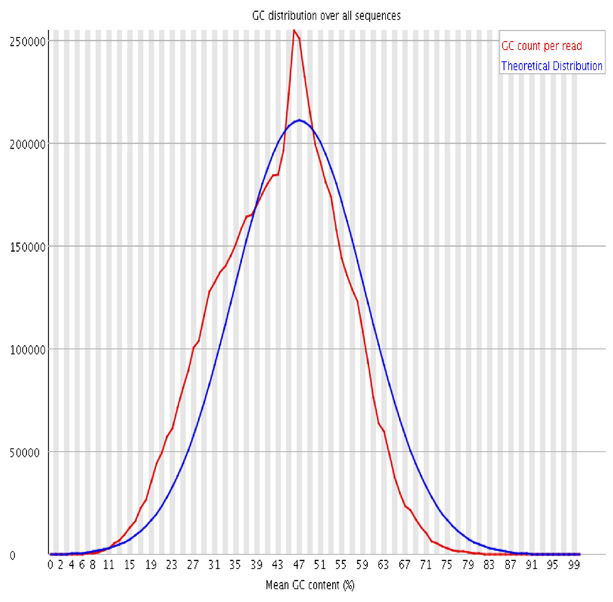

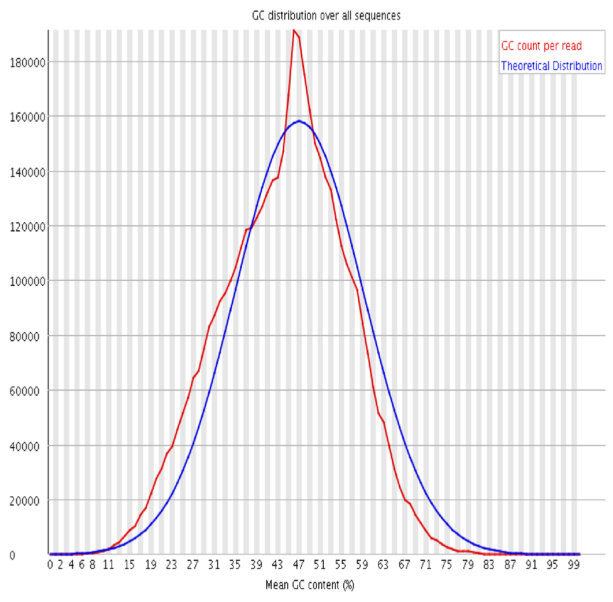

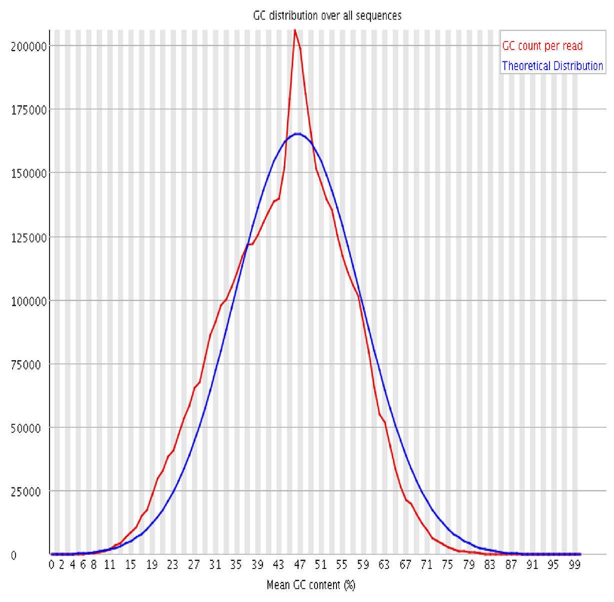

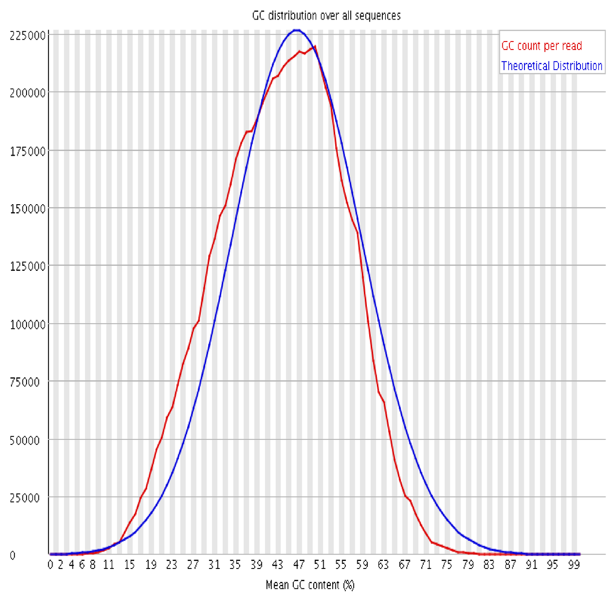

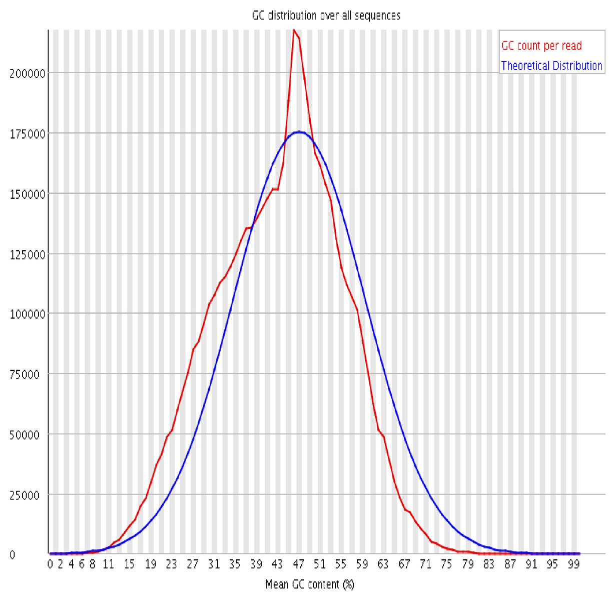

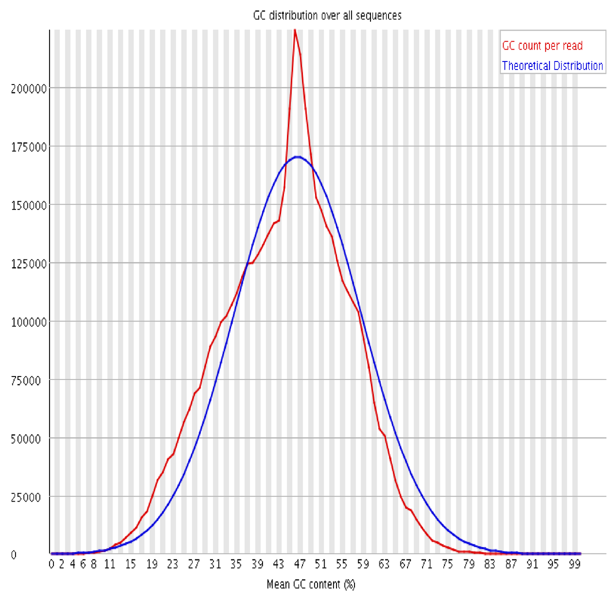

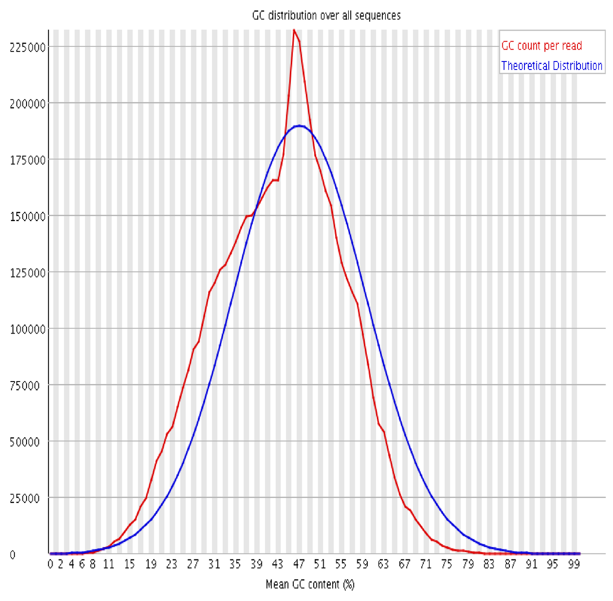

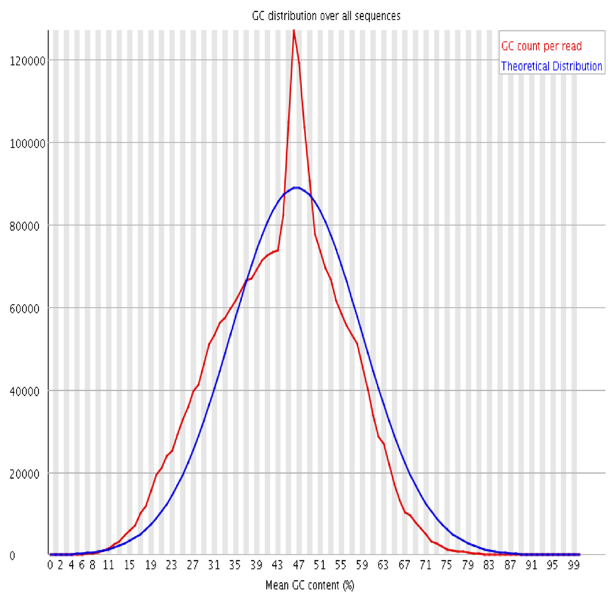

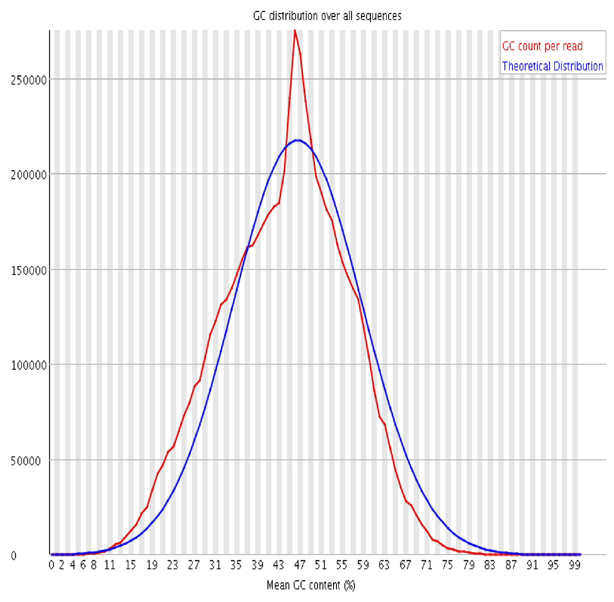

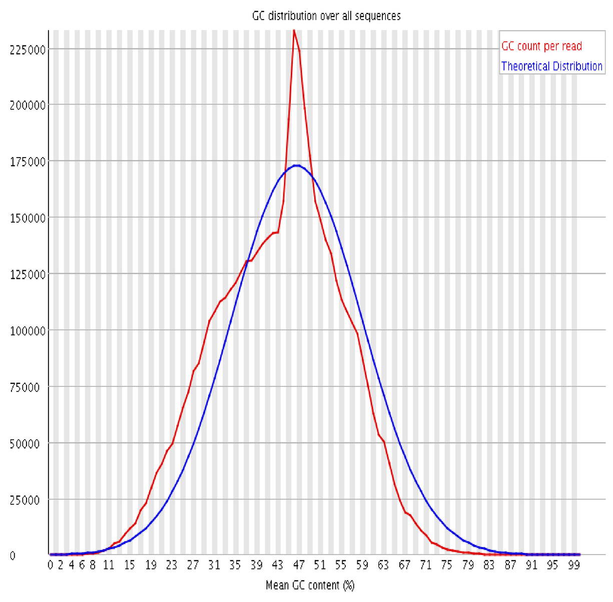

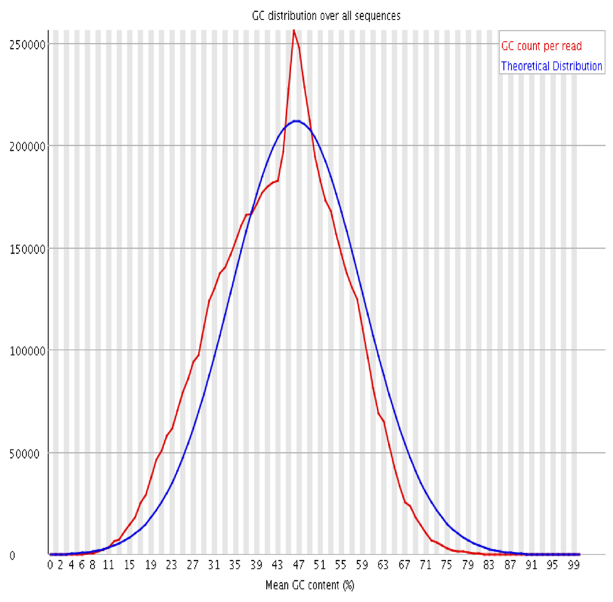

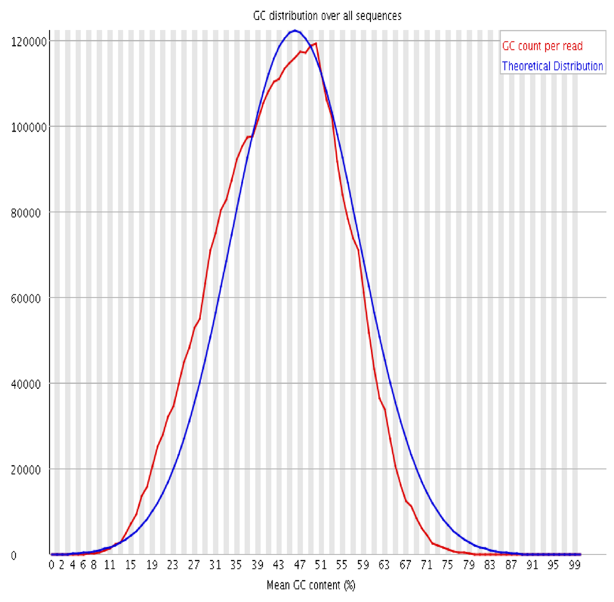

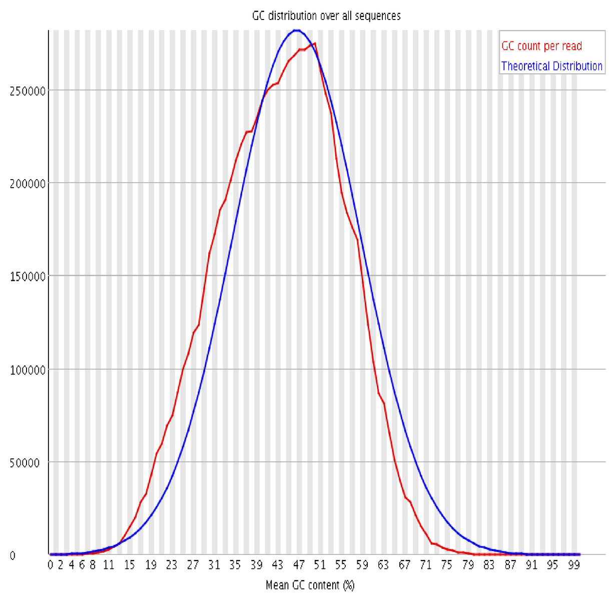

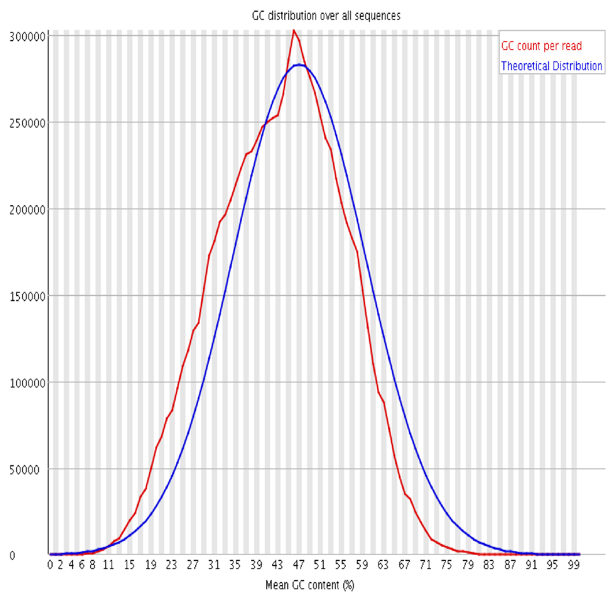

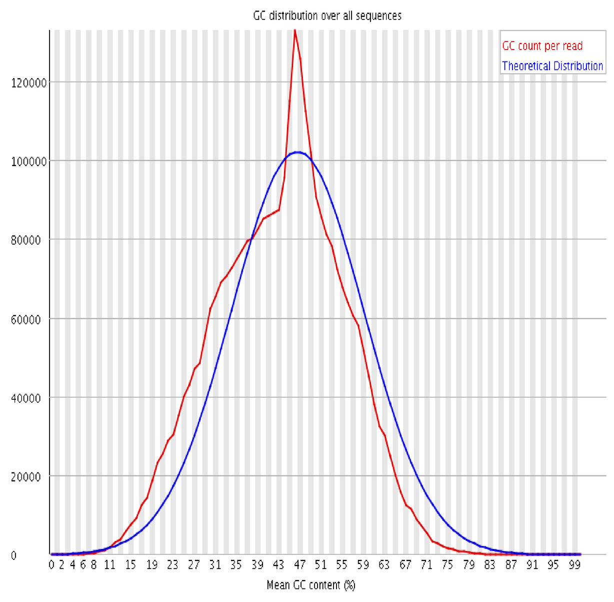

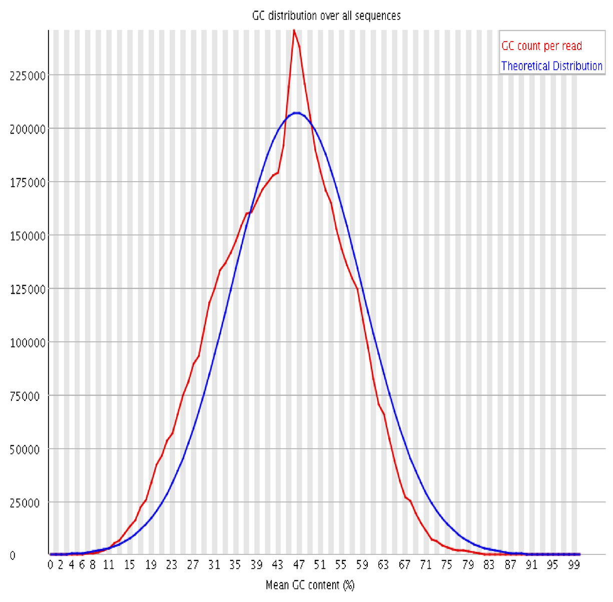

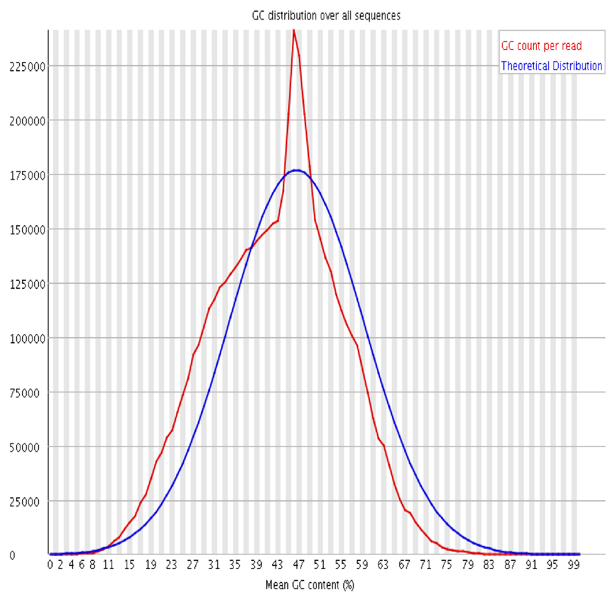

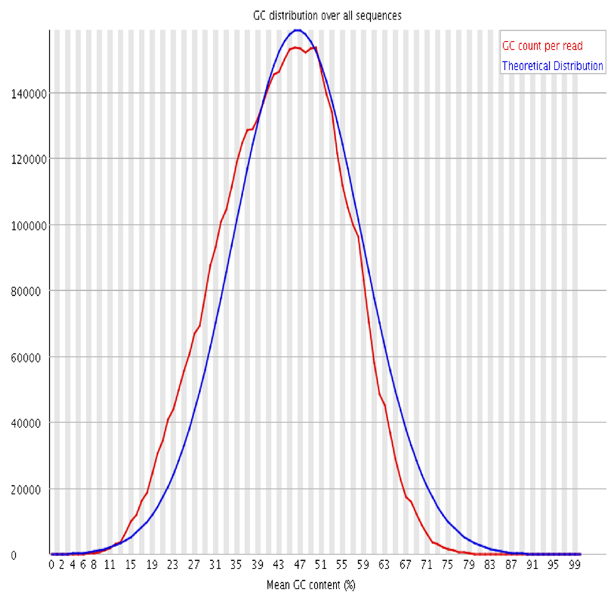

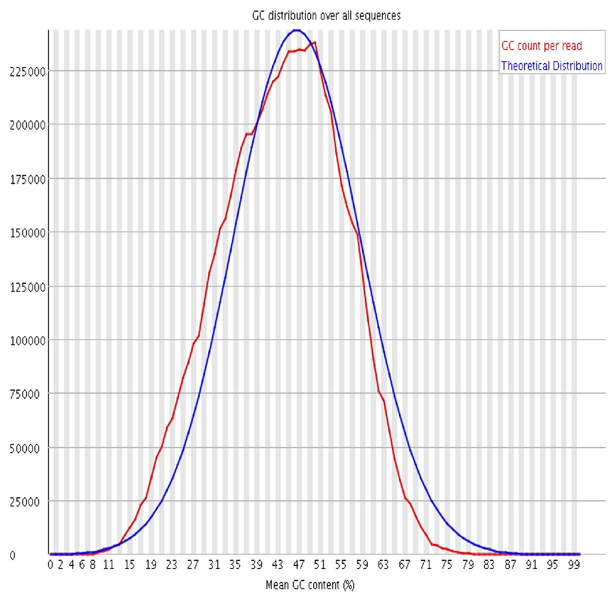

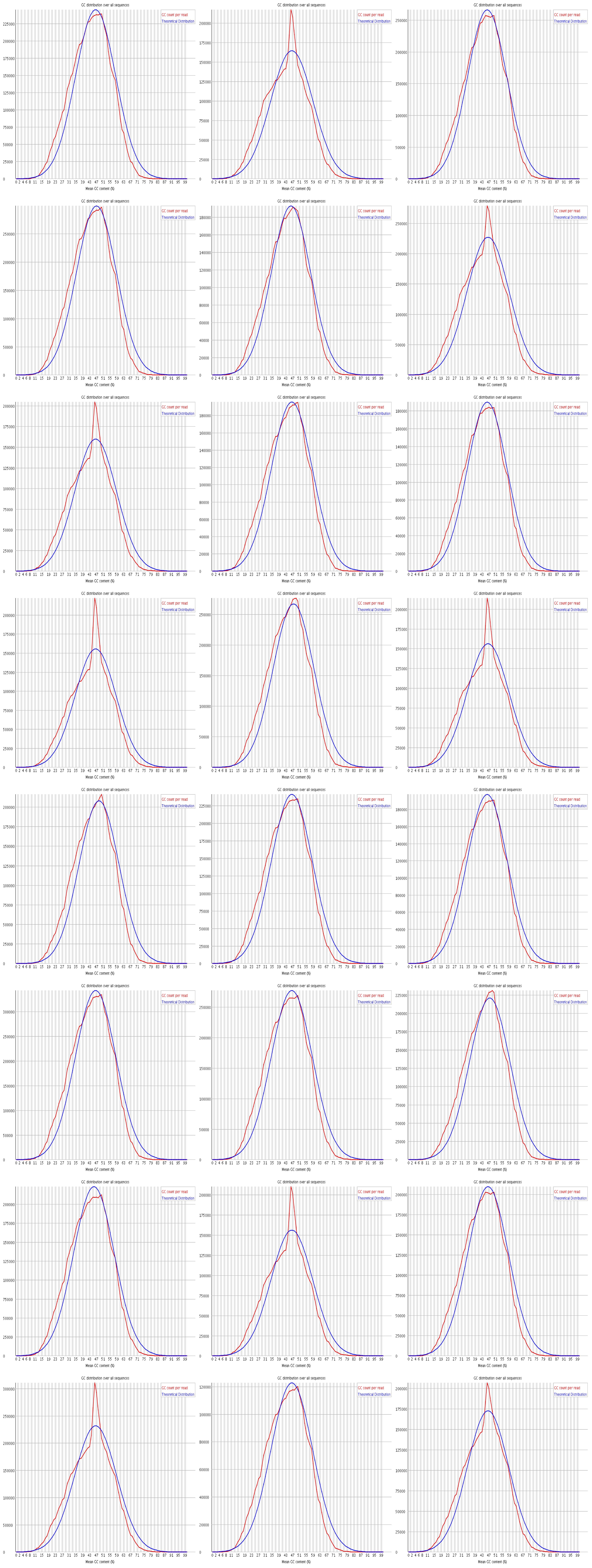
GC distribution over all sequences
GC count per read
Theoretical Distribution
Mean GC content (%)

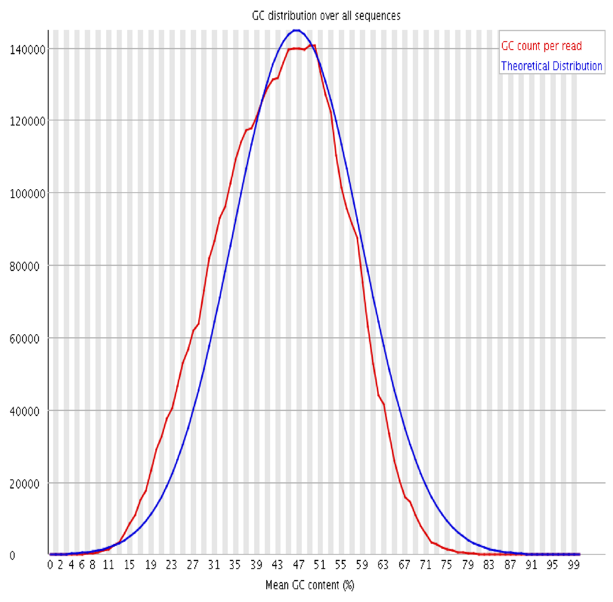
GC distribution over all sequences
GC count per read
Theoretical Distribution
0
20000
40000
60000
80000
100000
120000
140000
0 2 4 6 8 11 15 19 23 27 31 35 39 43 47 51 55 59 63 67 71 75 79 83 87 91 95 99
Mean GC content (%)

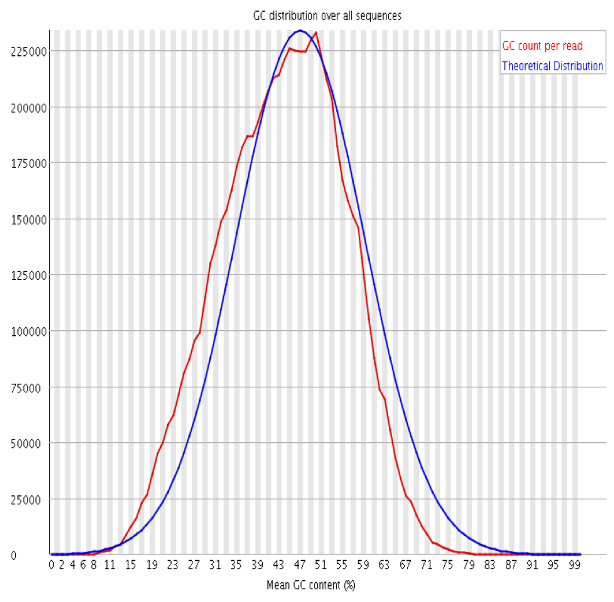
GC distribution over all sequences
GC count per read
Theoretical Distribution
0
25000
50000
75000
100000
125000
150000
175000
200000
225000
0 2 4 6 8 11 15 19 23 27 31 35 39 43 47 51 55 59 63 67 71 75 79 83 87 91 95 99
Mean GC content (%)

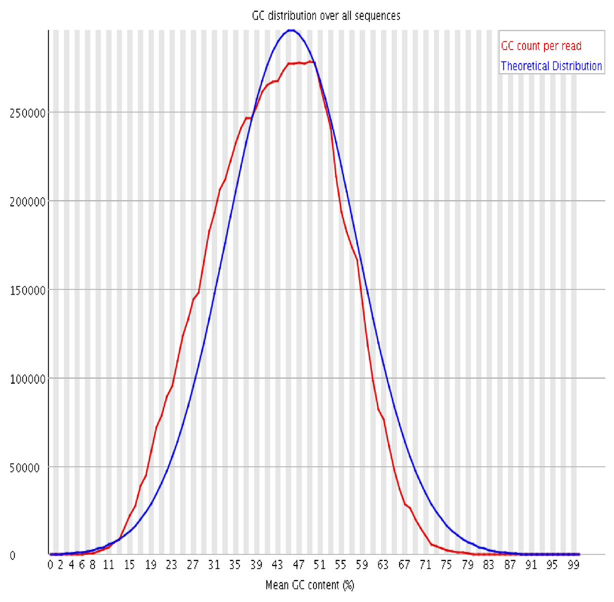
GC distribution over all sequences
GC count per read
Theoretical Distribution
0
50000
100000
150000
200000
250000
0 2 4 6 8 11 15 19 23 27 31 35 39 43 47 51 55 59 63 67 71 75 79 83 87 91 95 99
Mean GC content (%)

Normalized position vs. coverage

The following figures show the a plot of normalized position vs. coverage.

RNA–Seq Coverage vs. Transcript Position
All_Reads (SN163_BHT22GBCXX_0712_1_AGGCAT)
in file M101.sorted.merged.dedup.bam

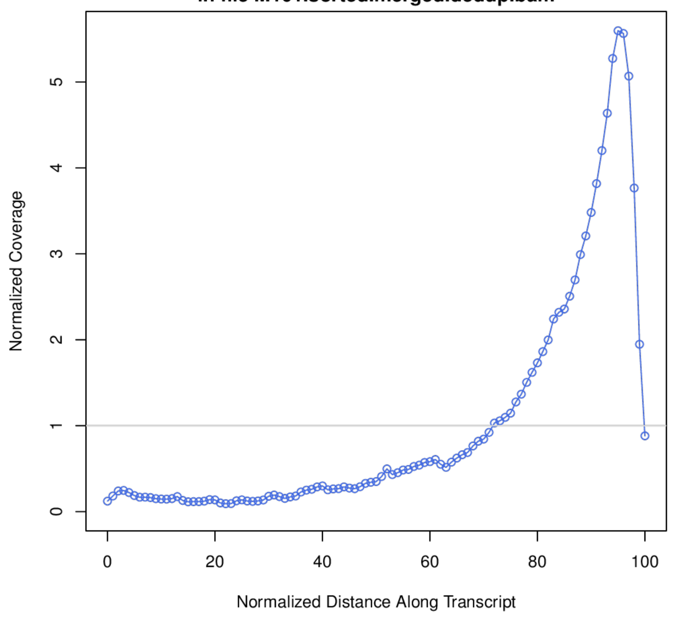

RNA–Seq Coverage vs. Transcript Position
All_Reads (SN163_BHT22GBCXX_0712_1_GAAGTG)
in file M103.sorted.merged.dedup.bam

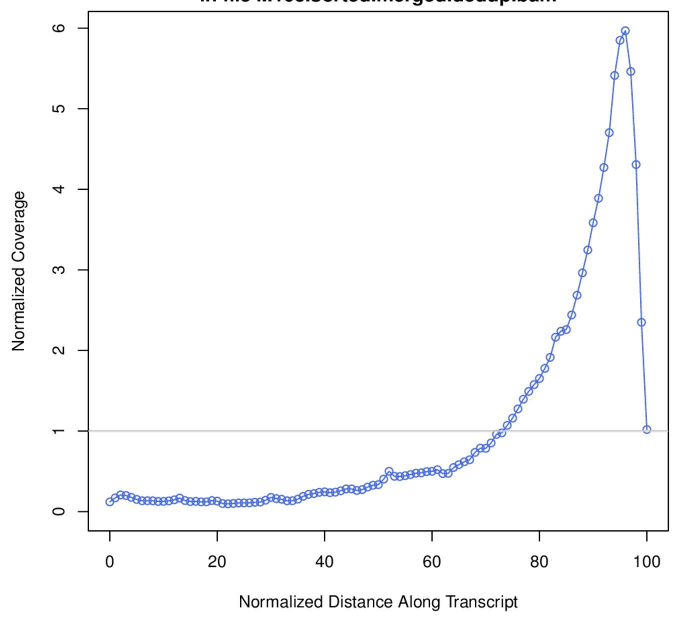

RNA–Seq Coverage vs. Transcript Position
All_Reads (SN163_BHT22GBCXX_0712_1_AACAAG)
in file M105.sorted.merged.dedup.bam

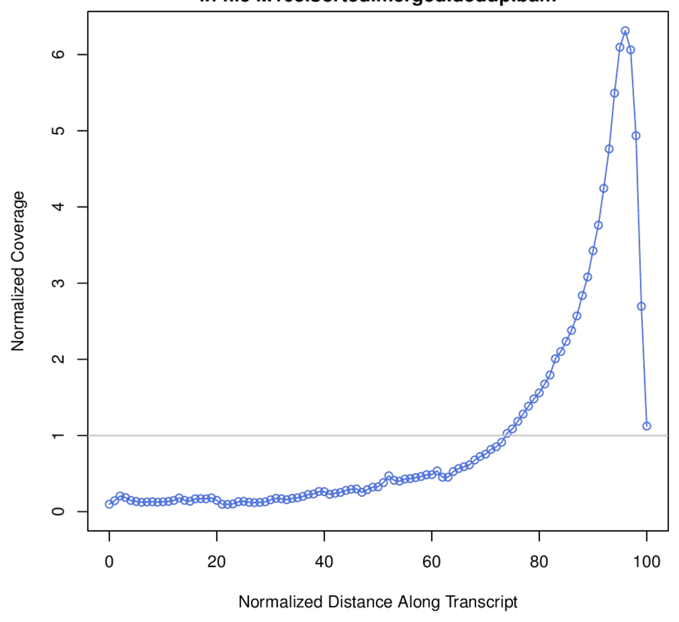

RNA–Seq Coverage vs. Transcript Position
All_Reads (SN163_BHT22GBCXX_0712_1_AAGTGG)
in file M107.sorted.merged.dedup.bam

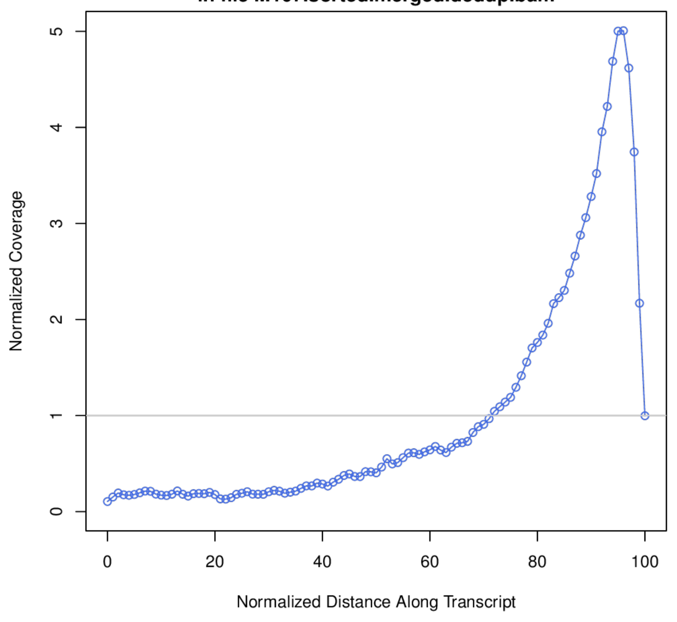

RNA–Seq Coverage vs. Transcript Position
All_Reads (SN163_BHT22GBCXX_0712_1_TACCTT)
in file M109.sorted.merged.dedup.bam

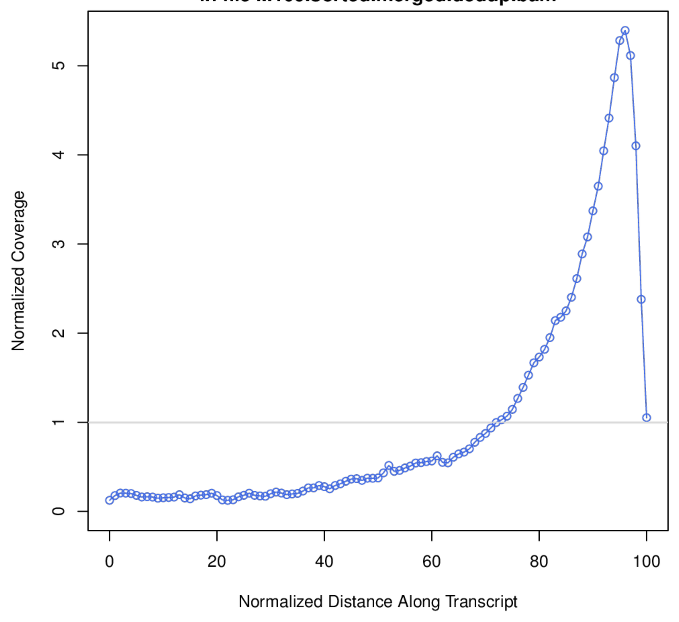

RNA–Seq Coverage vs. Transcript Position
All_Reads (SN163_BHT22GBCXX_0712_1_GTGCCA)
in file M11.sorted.merged.dedup.bam

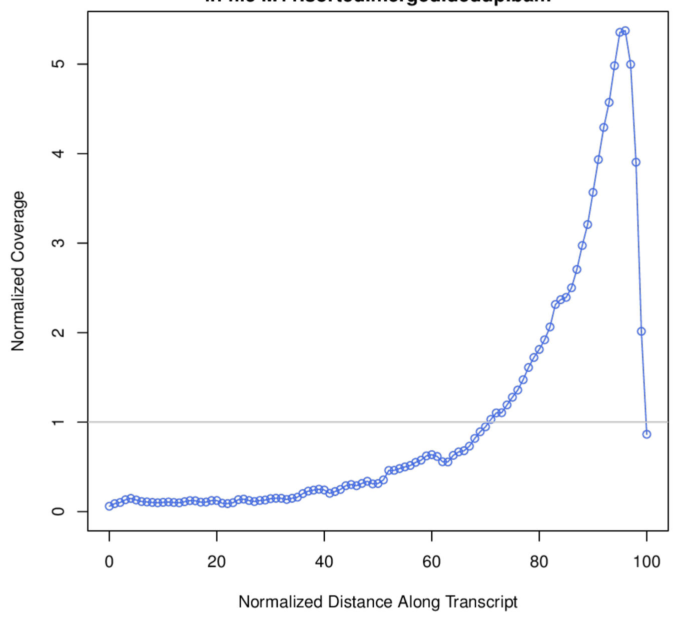

RNA–Seq Coverage vs. Transcript Position
All_Reads (SN163_BHT22GBCXX_0712_1_TTGGTA)
in file M111.sorted.merged.dedup.bam

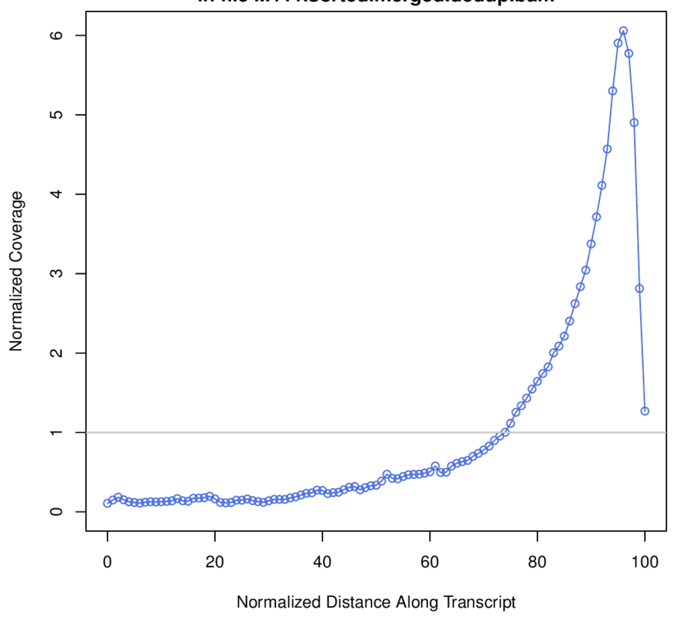

RNA–Seq Coverage vs. Transcript Position
All_Reads (SN163_BHT22GBCXX_0712_1_ACCTAC)
in file M113.sorted.merged.dedup.bam

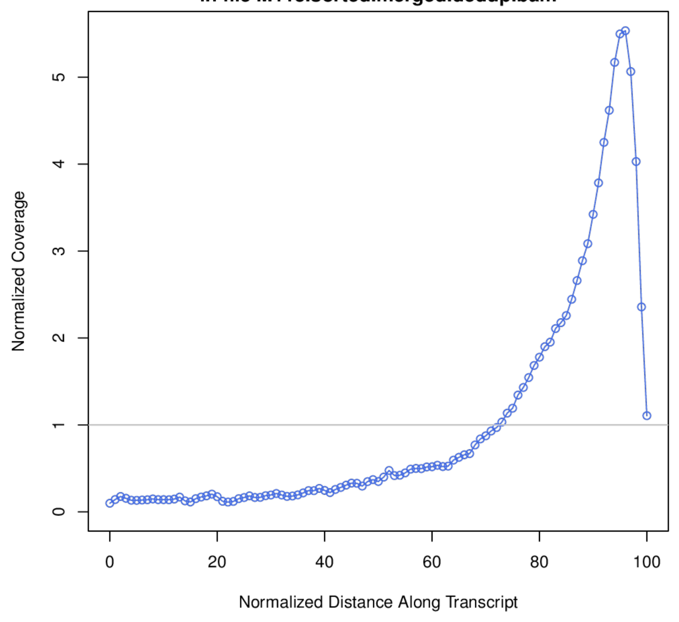

RNA–Seq Coverage vs. Transcript Position
All_Reads (SN163_BHT22GBCXX_0712_1_AGAATC)
in file M115.sorted.merged.dedup.bam

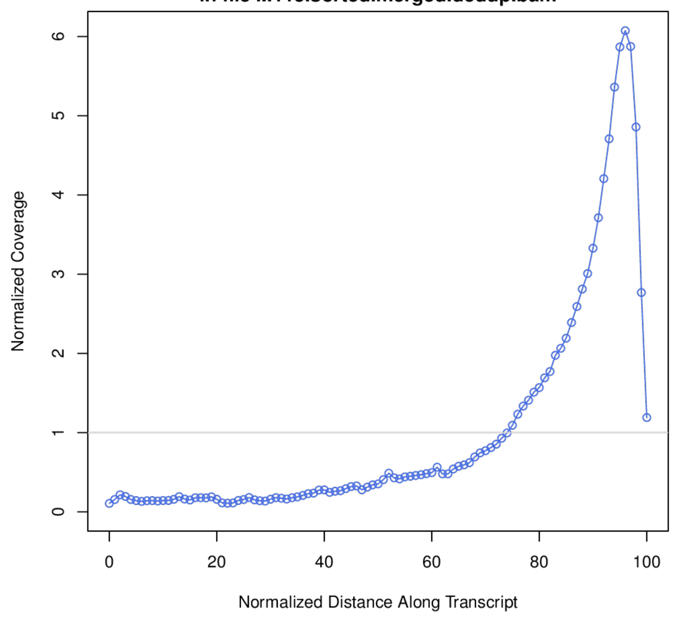

RNA–Seq Coverage vs. Transcript Position
All_Reads (SN163_BHT22GBCXX_0712_1_AACCGA)
in file M117.sorted.merged.dedup.bam

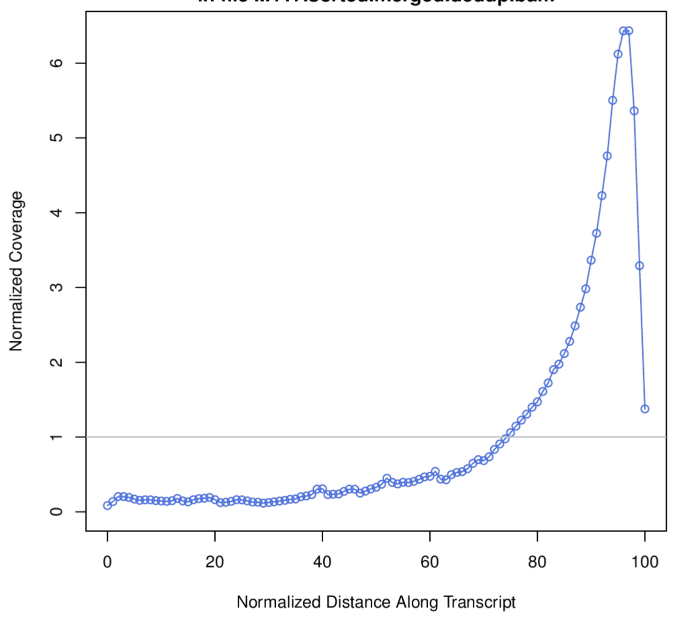

RNA–Seq Coverage vs. Transcript Position
All_Reads (SN163_BHT22GBCXX_0712_1_GGCCAA)
in file M119.sorted.merged.dedup.bam

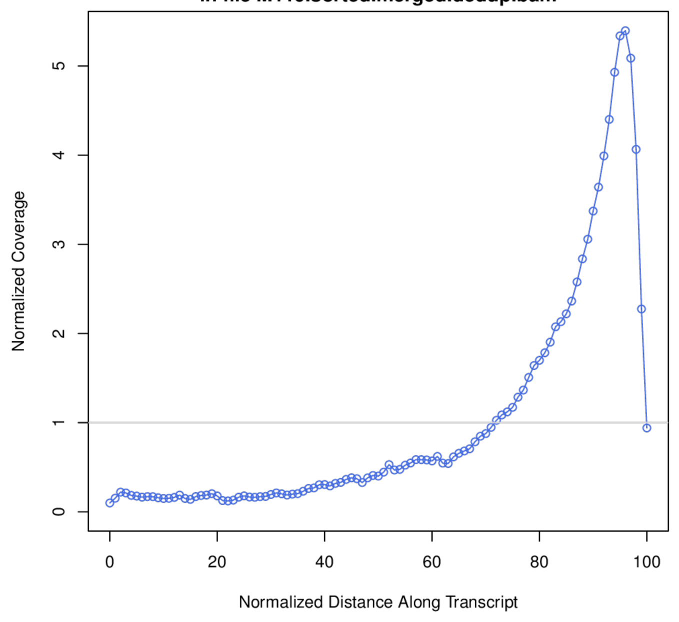

RNA–Seq Coverage vs. Transcript Position
All_Reads (SN163_BHT22GBCXX_0712_1_CAACAG)
in file M121.sorted.merged.dedup.bam

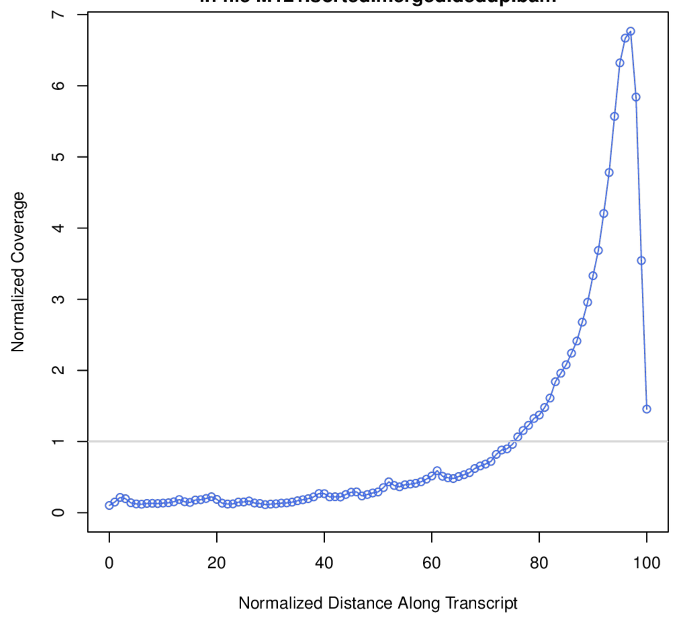

RNA–Seq Coverage vs. Transcript Position
All_Reads (SN163_BHT22GBCXX_0712_1_GCGAAT)
in file M13.sorted.merged.dedup.bam

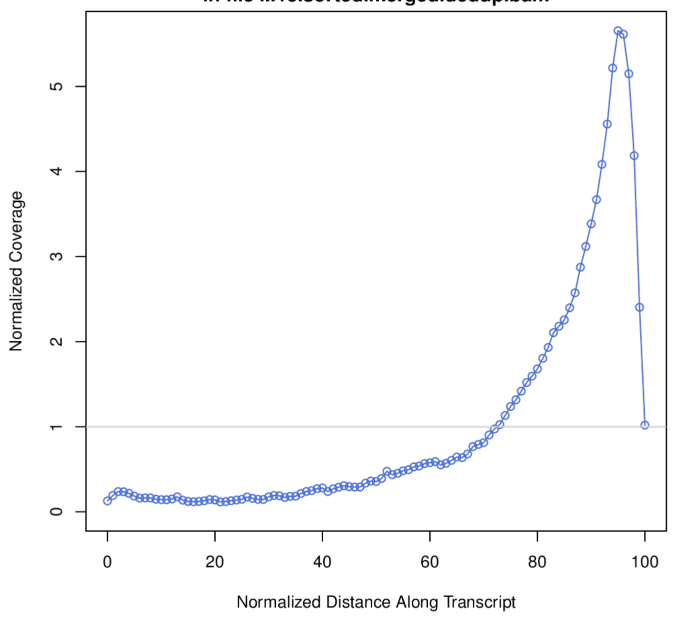

RNA–Seq Coverage vs. Transcript Position
All_Reads (SN163_BHT22GBCXX_0712_1_ACCGTG)
in file M15.sorted.merged.dedup.bam

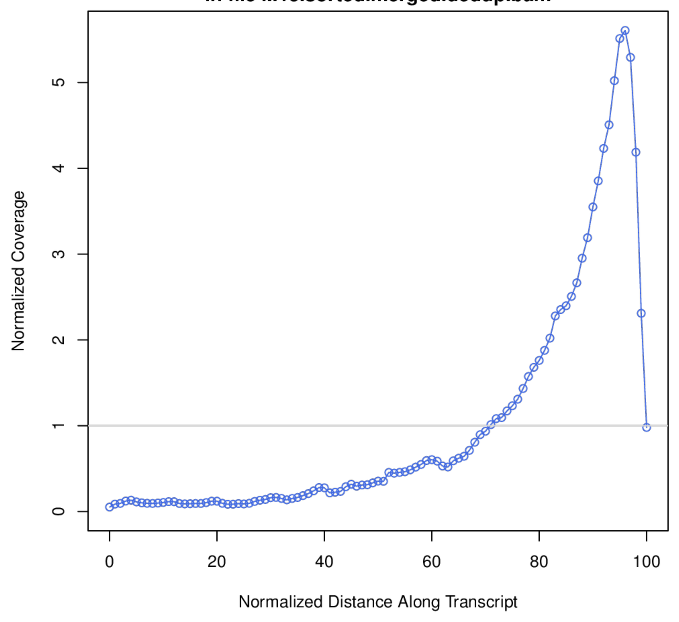

RNA–Seq Coverage vs. Transcript Position
All_Reads (SN163_BHT22GBCXX_0712_1_ACGTCT)
in file M17.sorted.merged.dedup.bam

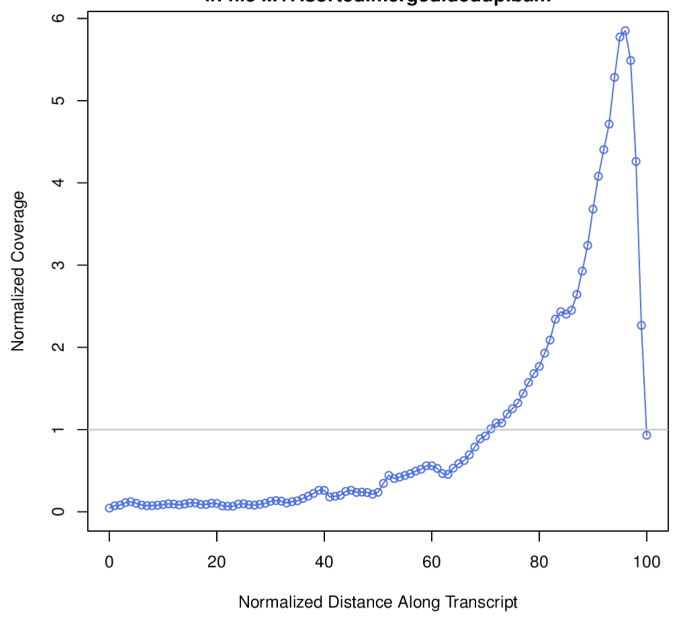

RNA–Seq Coverage vs. Transcript Position
All_Reads (SN163_BHT22GBCXX_0712_1_TGGATT)
in file M2.sorted.merged.dedup.bam

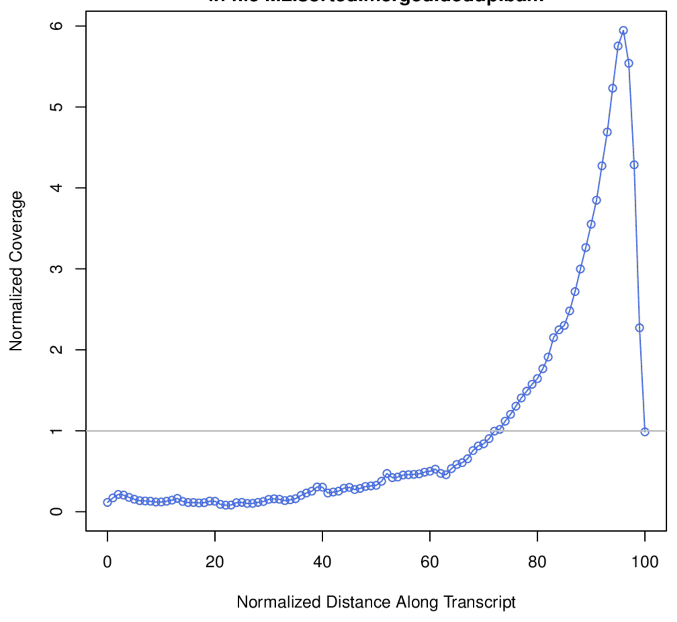

RNA–Seq Coverage vs. Transcript Position
All_Reads (SN163_BHT22GBCXX_0712_1_TGGCGA)
in file M20.sorted.merged.dedup.bam

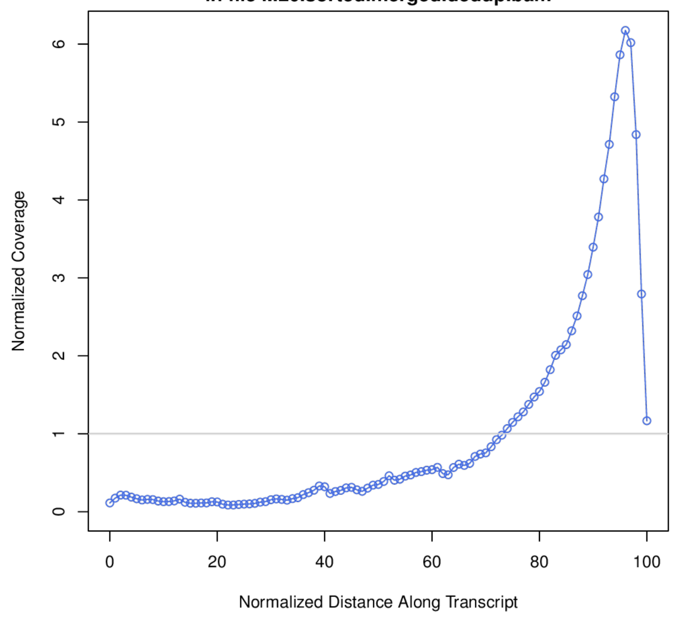

RNA–Seq Coverage vs. Transcript Position
All_Reads (SN163_BHT22GBCXX_0712_1_CAATGC)
in file M24.sorted.merged.dedup.bam

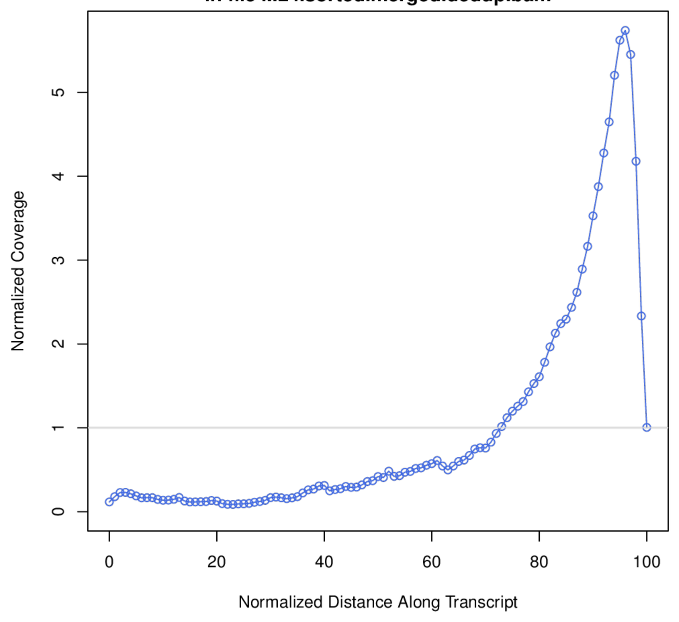

RNA–Seq Coverage vs. Transcript Position
All_Reads (SN163_BHT22GBCXX_0712_1_GCAGCC)
in file M26.sorted.merged.dedup.bam

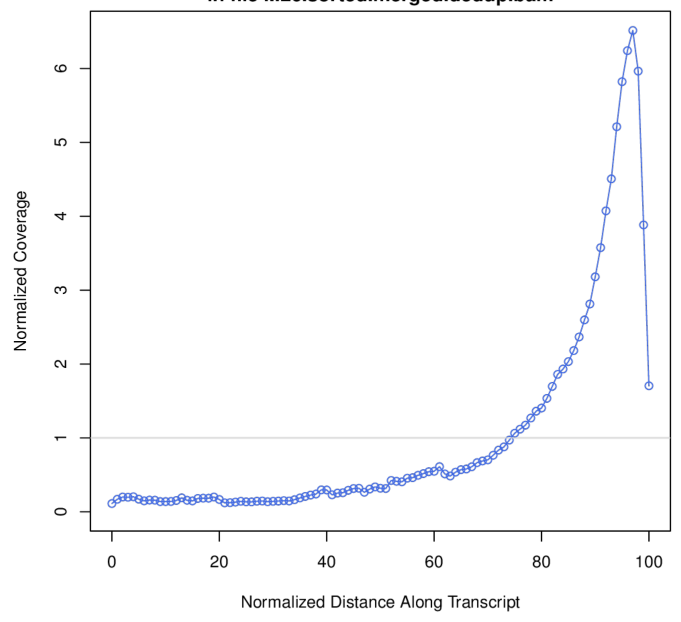

RNA–Seq Coverage vs. Transcript Position
All_Reads (SN163_BHT22GBCXX_0712_1_AGACCA)
in file M29.sorted.merged.dedup.bam

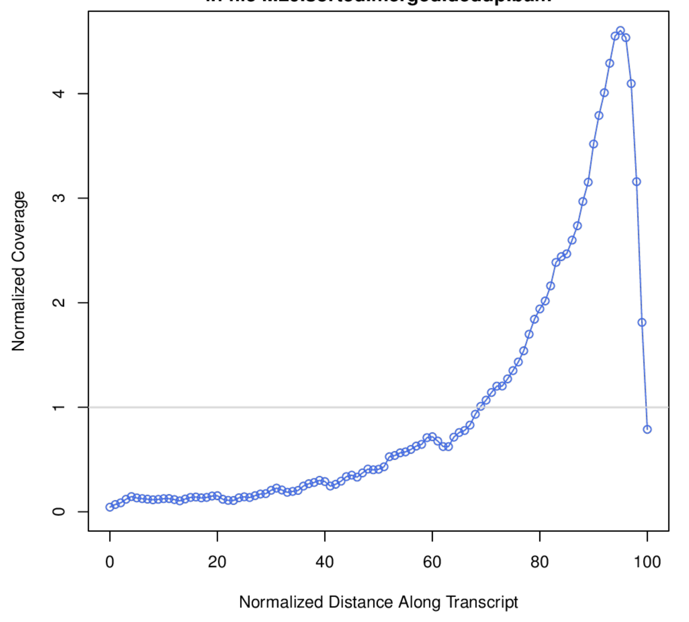

RNA–Seq Coverage vs. Transcript Position
All_Reads (SN163_BHT22GBCXX_0712_1_GATTGT)
in file M4.sorted.merged.dedup.bam

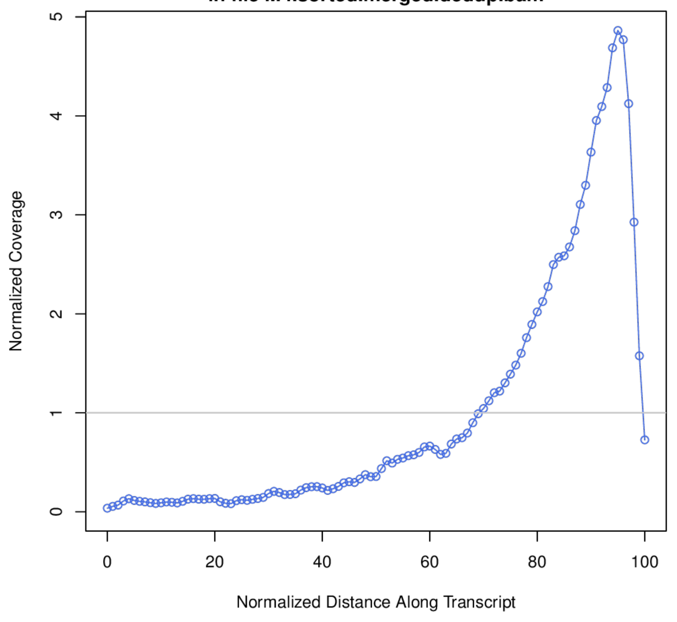

RNA–Seq Coverage vs. Transcript Position
All_Reads (SN163_BHT22GBCXX_0712_1_TGTGCA)
in file M47.sorted.merged.dedup.bam

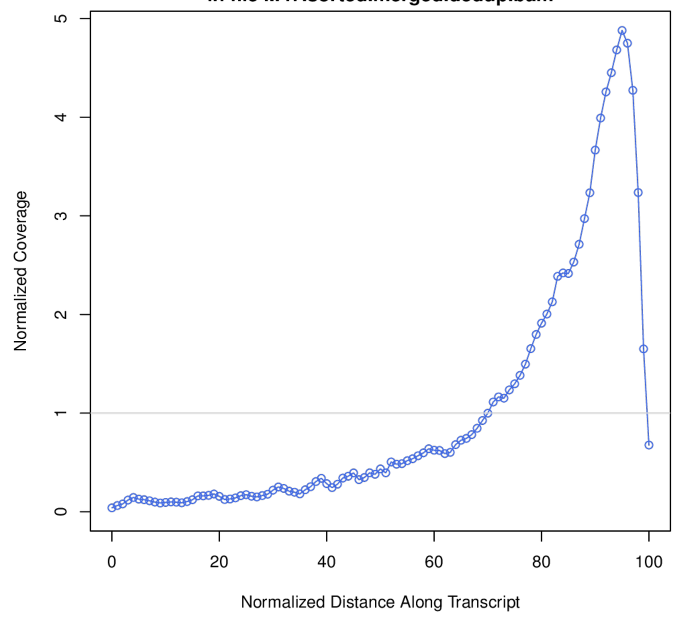

RNA–Seq Coverage vs. Transcript Position
All_Reads (SN163_BHT22GBCXX_0712_1_CGATCT)
in file M49.sorted.merged.dedup.bam

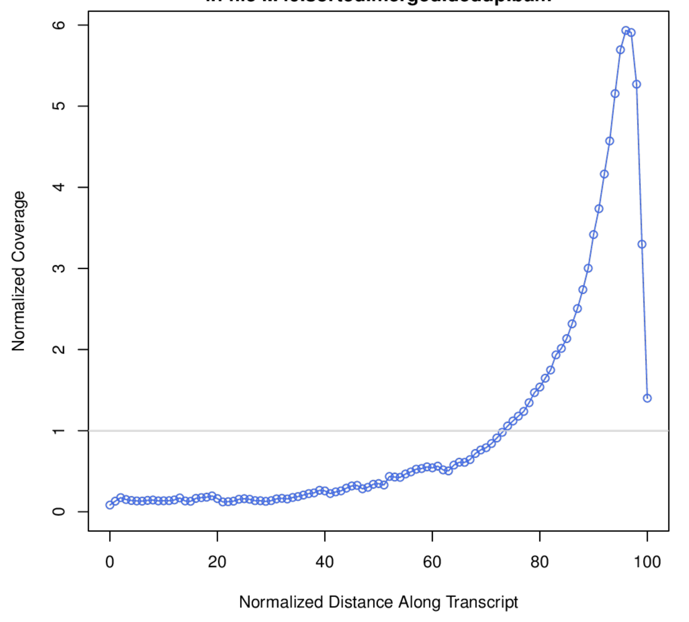

RNA–Seq Coverage vs. Transcript Position
All_Reads (SN163_BHT22GBCXX_0712_1_GTCAGG)
in file M51.sorted.merged.dedup.bam

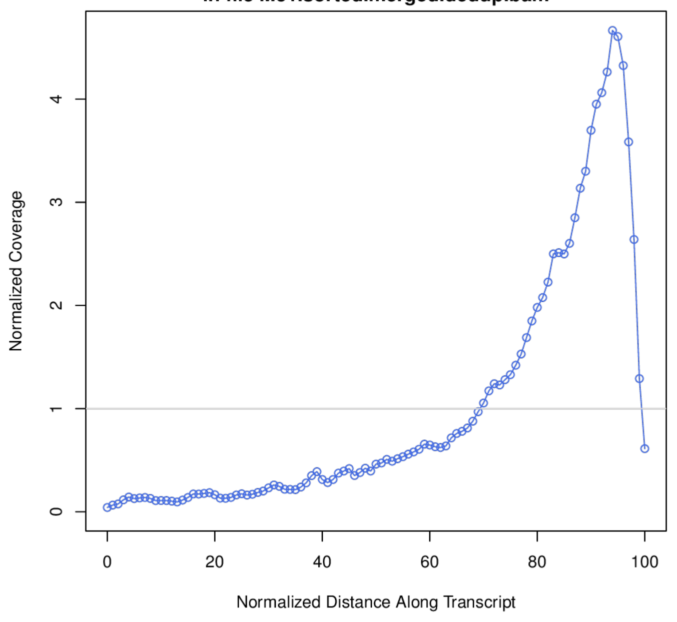

RNA–Seq Coverage vs. Transcript Position
All_Reads (SN163_BHT22GBCXX_0712_1_GTAGAA)
in file M53.sorted.merged.dedup.bam

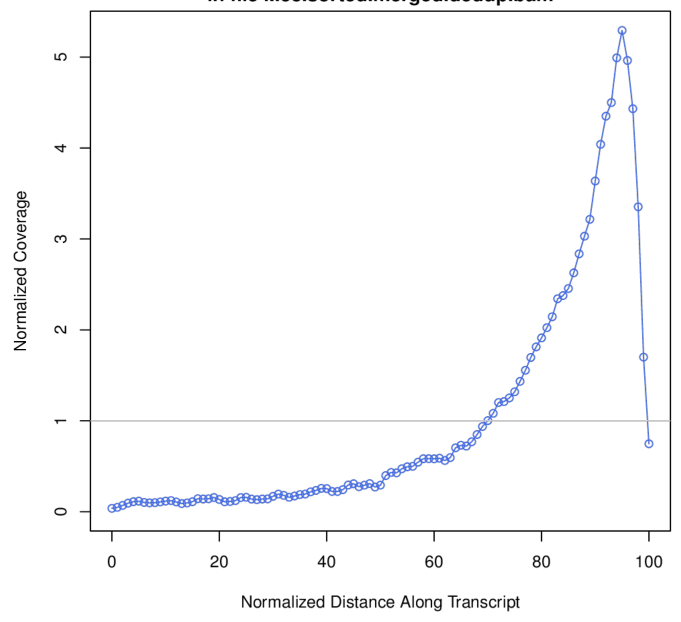

RNA–Seq Coverage vs. Transcript Position
All_Reads (SN163_BHT22GBCXX_0712_1_ACAGAT)
in file M55.sorted.merged.dedup.bam

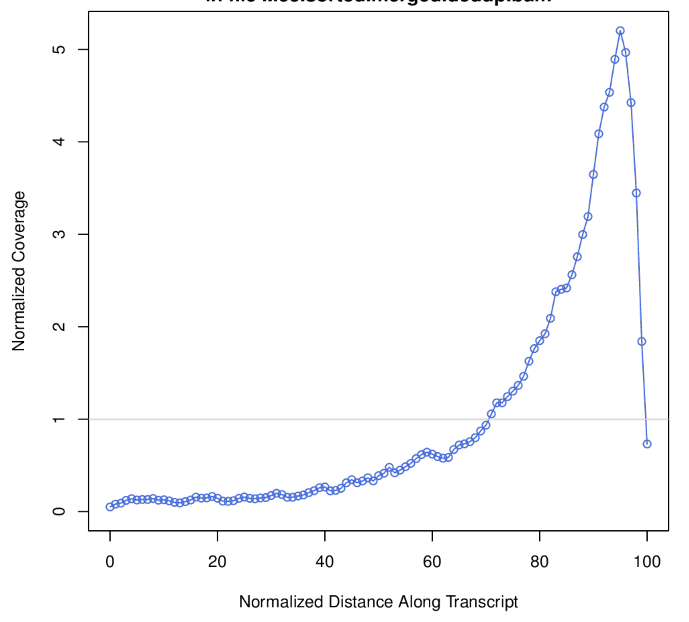

RNA–Seq Coverage vs. Transcript Position
All_Reads (SN163_BHT22GBCXX_0712_1_CACTAA)
in file M57.sorted.merged.dedup.bam

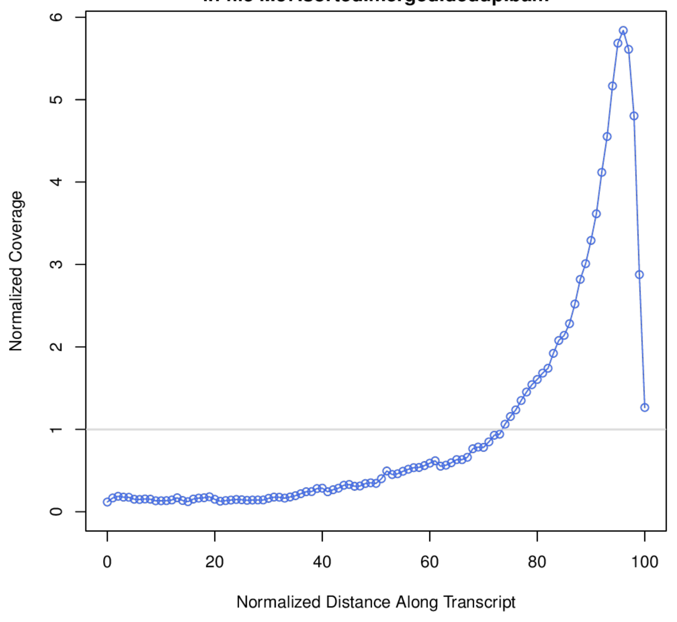

RNA–Seq Coverage vs. Transcript Position
All_Reads (SN163_BHT22GBCXX_0712_1_GGAGGT)
in file M59.sorted.merged.dedup.bam

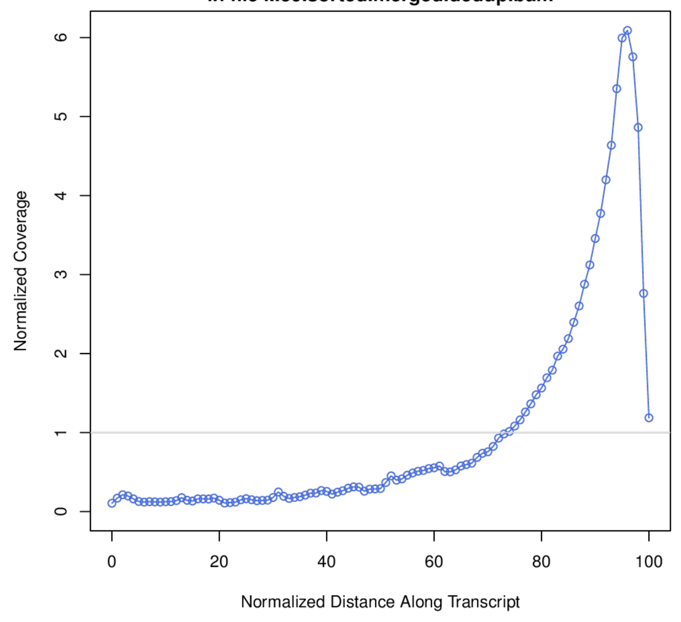

RNA–Seq Coverage vs. Transcript Position
All_Reads (SN163_BHT22GBCXX_0712_1_AATGAA)
in file M6.sorted.merged.dedup.bam

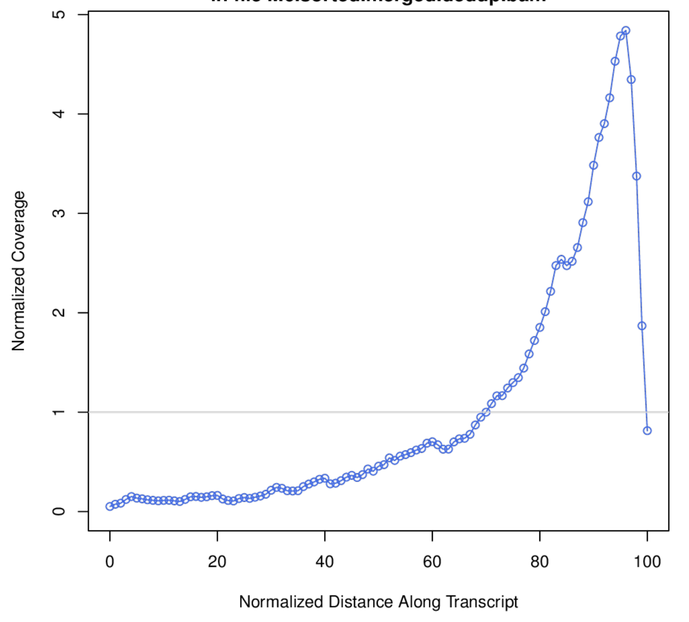

RNA–Seq Coverage vs. Transcript Position
All_Reads (SN163_BHT22GBCXX_0712_1_ACACGC)
in file M61.sorted.merged.dedup.bam

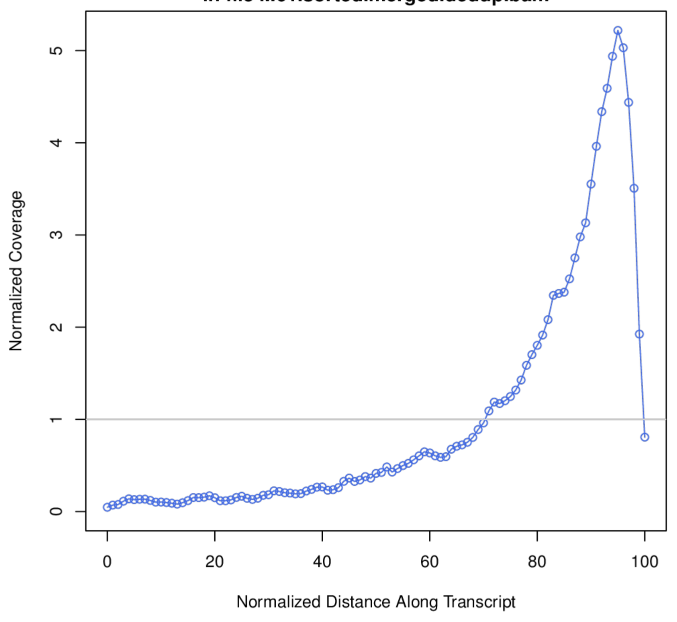

RNA–Seq Coverage vs. Transcript Position
All_Reads (SN163_BHT22GBCXX_0712_1_CGCCTG)
in file M63.sorted.merged.dedup.bam

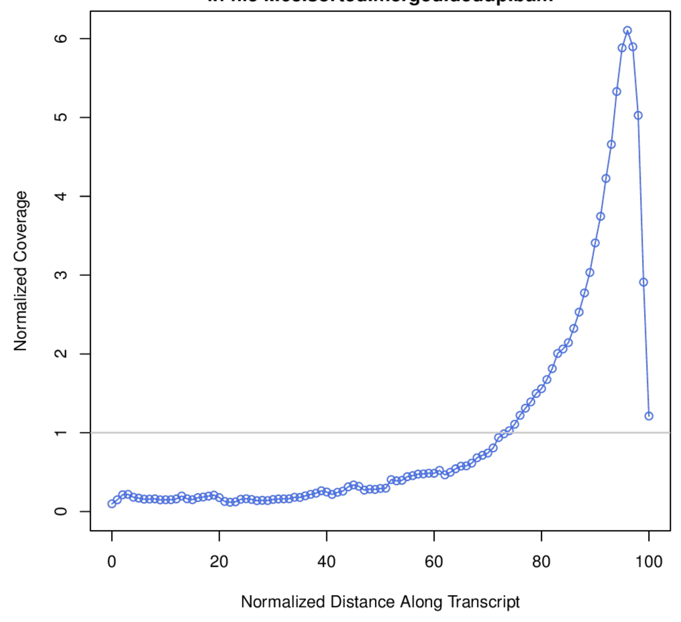

RNA–Seq Coverage vs. Transcript Position
All_Reads (SN163_BHT22GBCXX_0712_1_GTGTAG)
in file M65.sorted.merged.dedup.bam

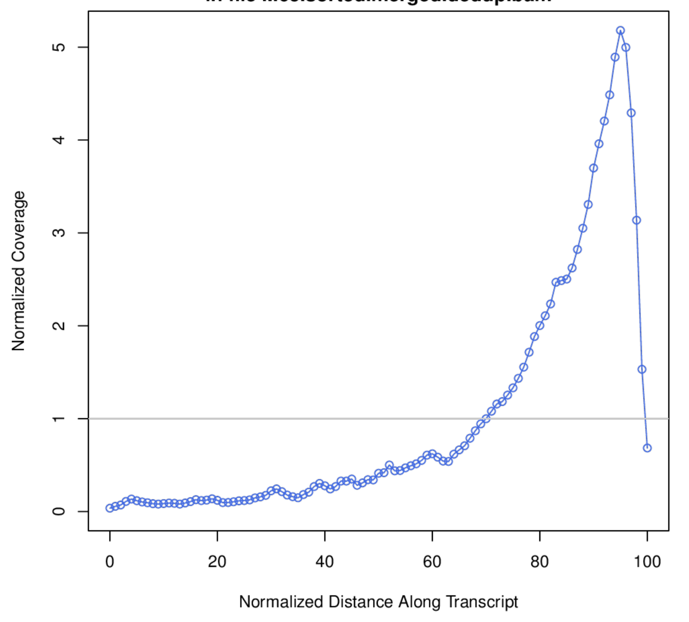

RNA–Seq Coverage vs. Transcript Position
All_Reads (SN163_BHT22GBCXX_0712_1_CATCTA)
in file M67.sorted.merged.dedup.bam

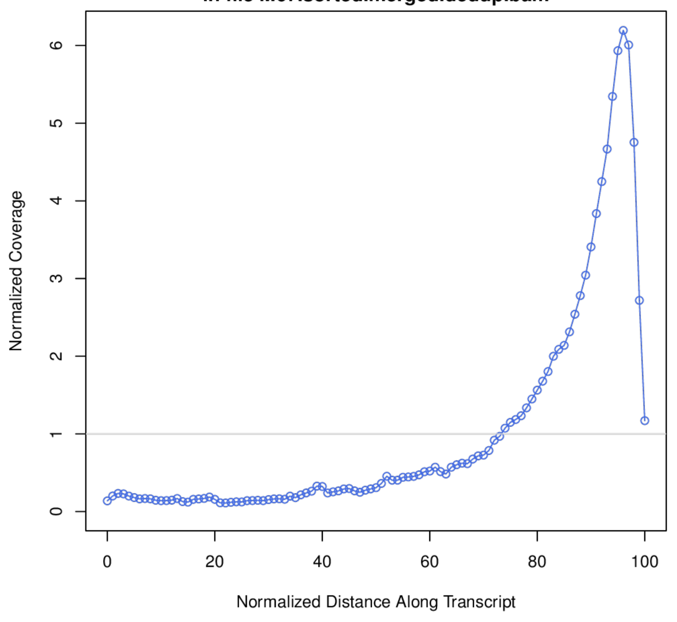

RNA–Seq Coverage vs. Transcript Position
All_Reads (SN163_BHT22GBCXX_0712_1_GGTATA)
in file M69.sorted.merged.dedup.bam

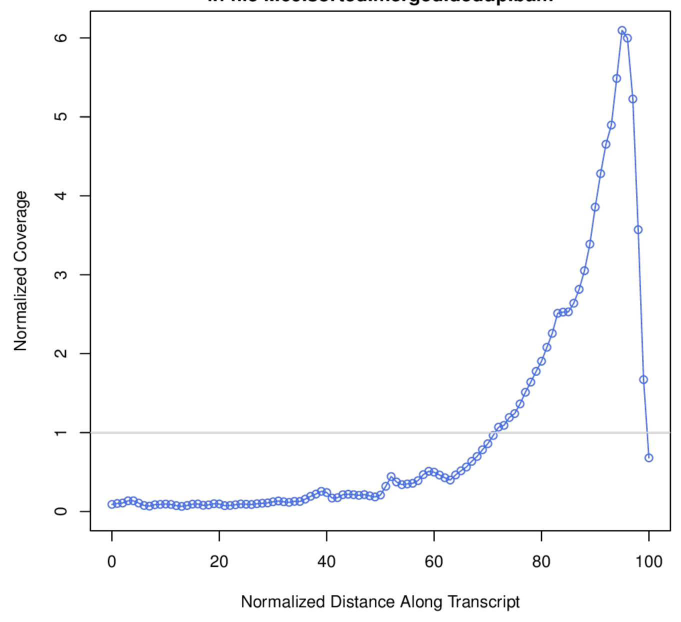

RNA–Seq Coverage vs. Transcript Position
All_Reads (SN163_BHT22GBCXX_0712_1_ACTCTT)
in file M71.sorted.merged.dedup.bam

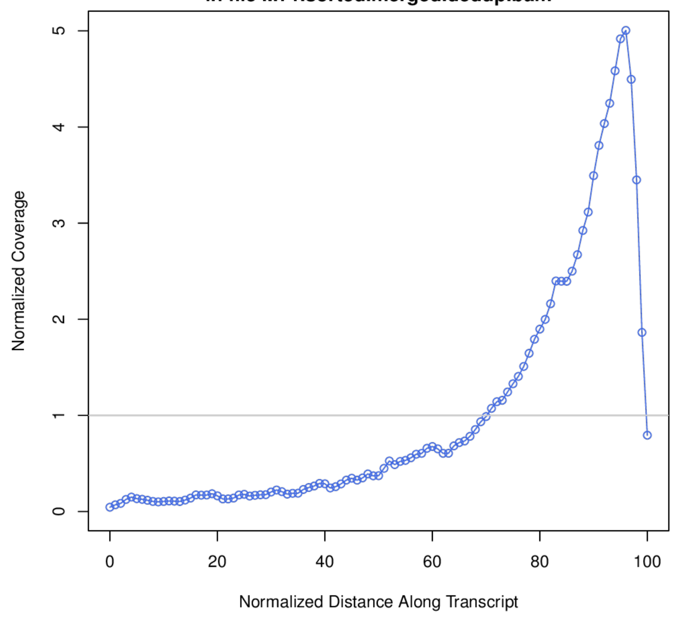

RNA–Seq Coverage vs. Transcript Position
All_Reads (SN163_BHT22GBCXX_0712_1_TTCCGC)
in file M73.sorted.merged.dedup.bam

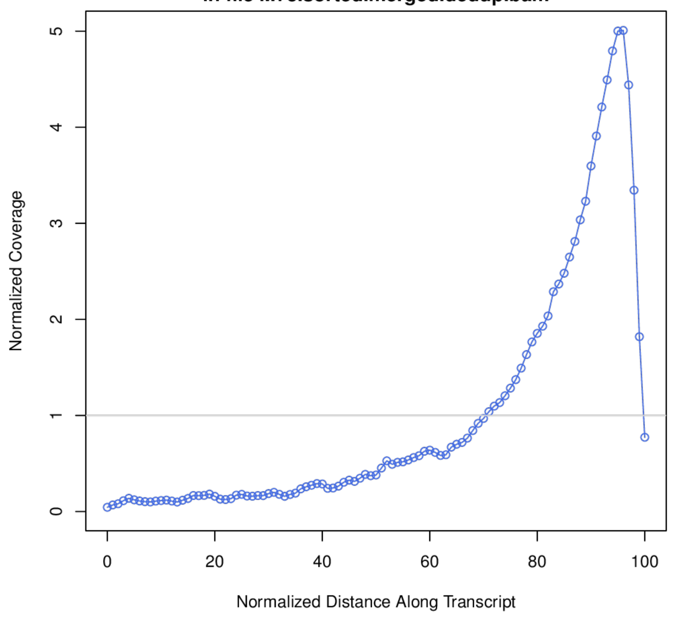

RNA–Seq Coverage vs. Transcript Position
All_Reads (SN163_BHT22GBCXX_0712_1_ATAAGA)
in file M75.sorted.merged.dedup.bam

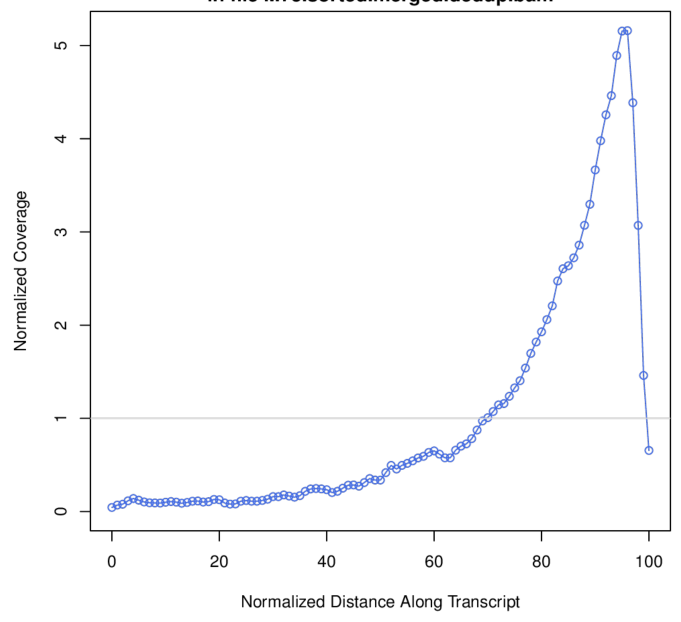

RNA–Seq Coverage vs. Transcript Position
All_Reads (SN163_BHT22GBCXX_0712_1_AGATAG)
in file M77.sorted.merged.dedup.bam

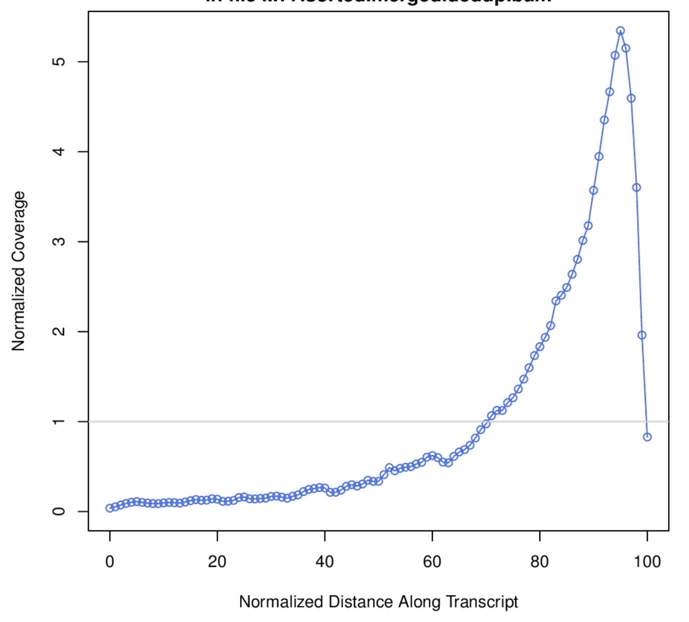

RNA–Seq Coverage vs. Transcript Position
All_Reads (SN163_BHT22GBCXX_0712_1_ACCAGT)
in file M8.sorted.merged.dedup.bam

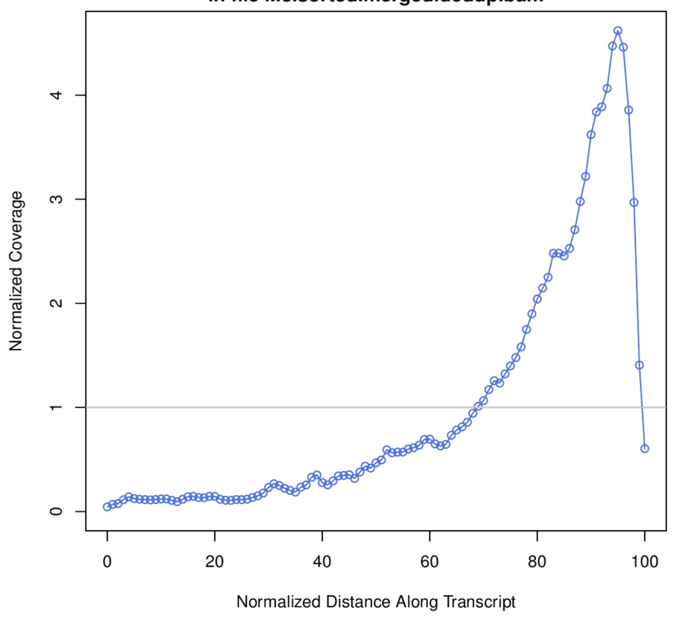

RNA–Seq Coverage vs. Transcript Position
All_Reads (SN163_BHT22GBCXX_0712_1_ATACTG)
in file M80.sorted.merged.dedup.bam

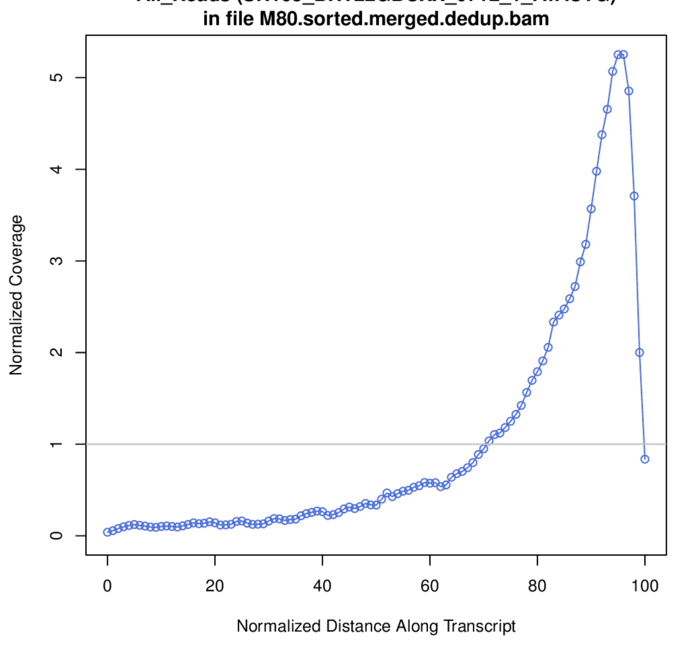

RNA–Seq Coverage vs. Transcript Position
All_Reads (SN163_BHT22GBCXX_0712_1_AAGCTC)
in file M83.sorted.merged.dedup.bam

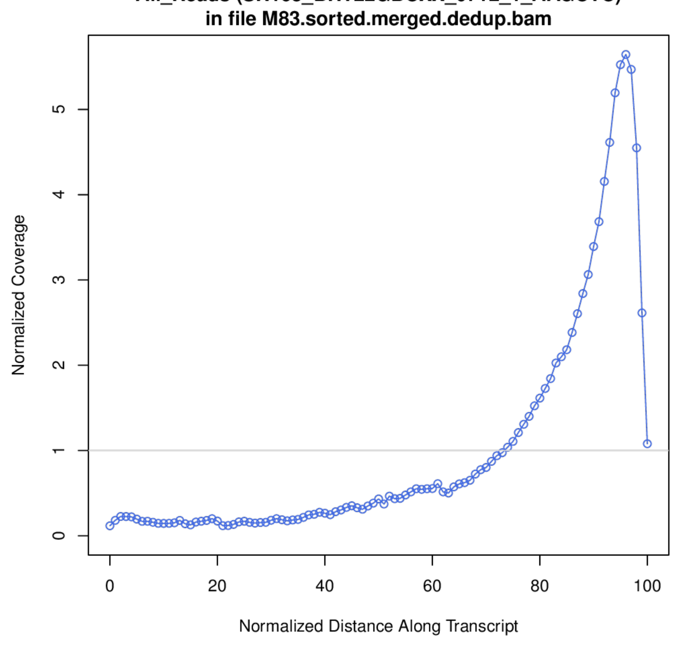

RNA–Seq Coverage vs. Transcript Position
All_Reads (SN163_BHT22GBCXX_0712_1_GAGTCC)
in file M85.sorted.merged.dedup.bam

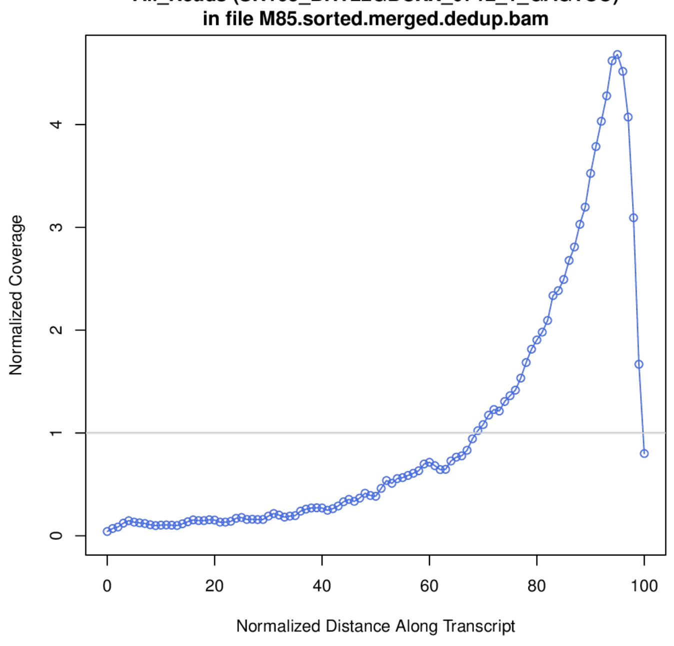

RNA–Seq Coverage vs. Transcript Position
All_Reads (SN163_BHT22GBCXX_0712_1_CAGGAC)
in file M87.sorted.merged.dedup.bam

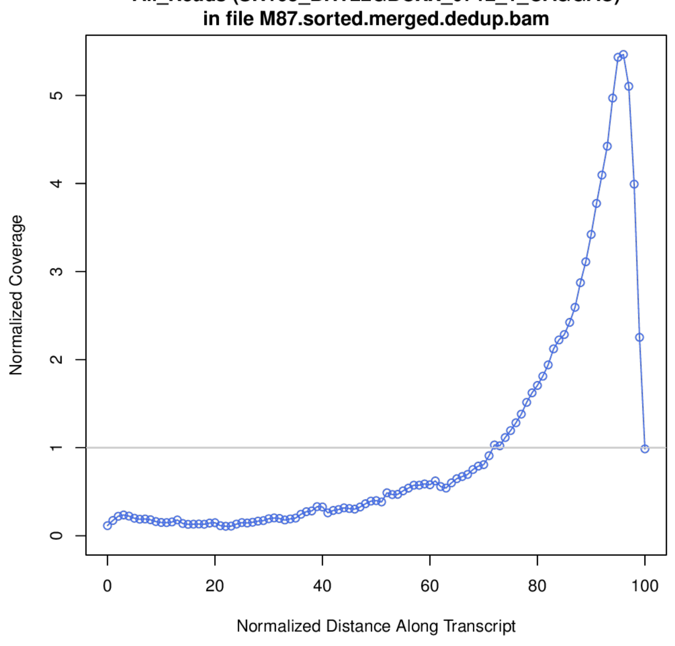

RNA–Seq Coverage vs. Transcript Position
All_Reads (SN163_BHT22GBCXX_0712_1_GCTCGA)
in file M90.sorted.merged.dedup.bam

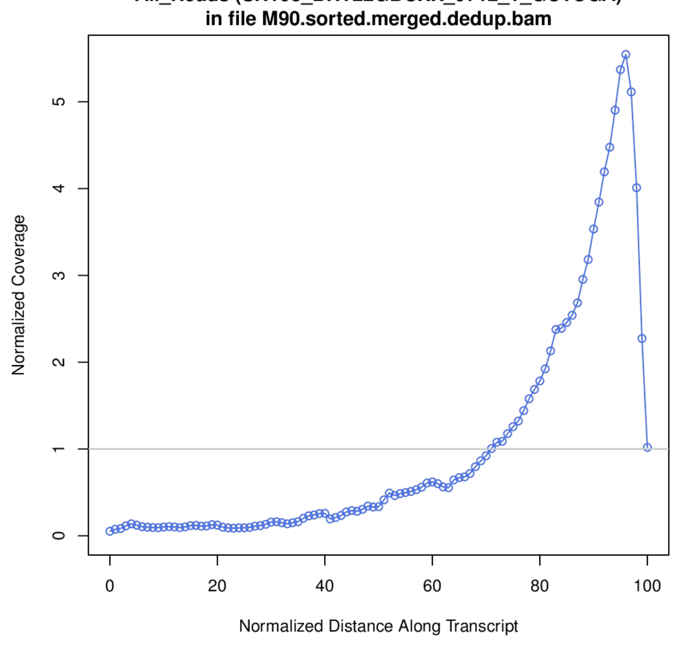

RNA–Seq Coverage vs. Transcript Position
All_Reads (SN163_BHT22GBCXX_0712_1_CCGACC)
in file M92.sorted.merged.dedup.bam

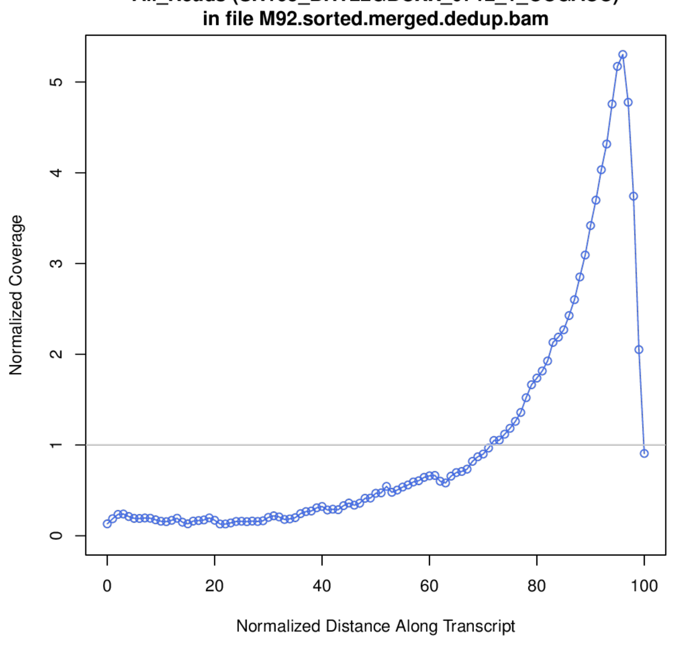

RNA–Seq Coverage vs. Transcript Position
All_Reads (SN163_BHT22GBCXX_0712_1_AGTACT)
in file M94.sorted.merged.dedup.bam

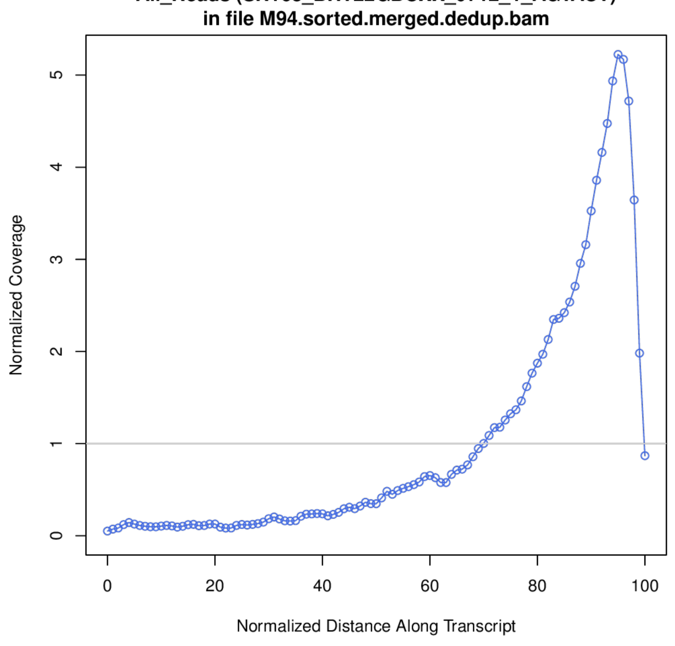

RNA–Seq Coverage vs. Transcript Position
All_Reads (SN163_BHT22GBCXX_0712_1_TCAGGA)
in file M97.sorted.merged.dedup.bam

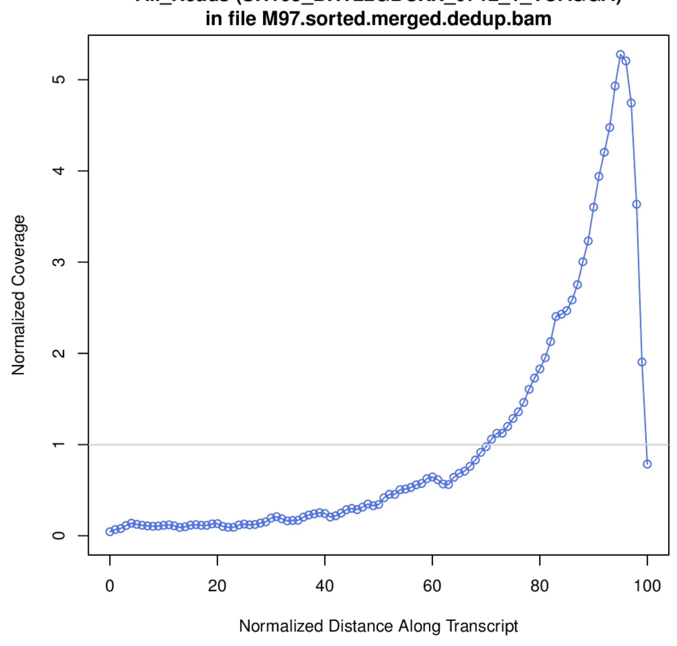

RNA–Seq Coverage vs. Transcript Position
All_Reads (SN163_BHT22GBCXX_0712_1_TAGGCT)
in file M99.sorted.merged.dedup.bam

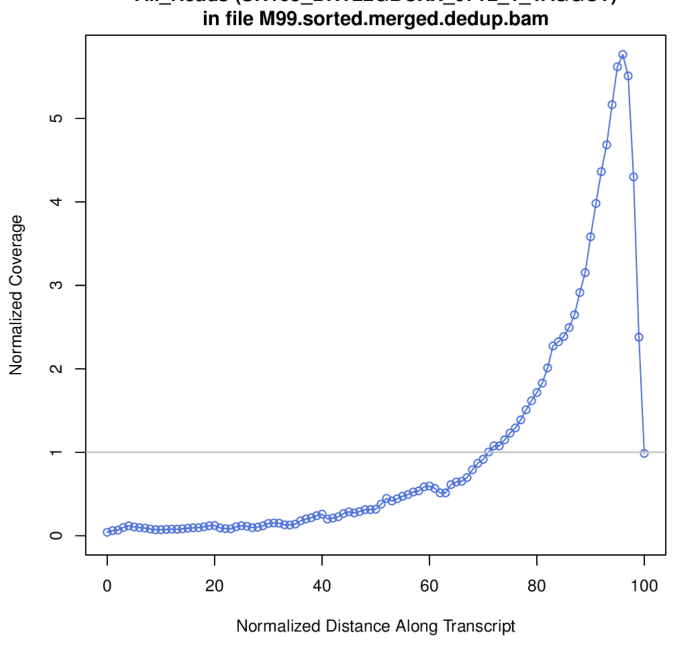

Supplement: Supplementary file 3 — Data S1. The R‐code to reproduce the gene expression analysis. [file PHY2-5-e13376-s003.zip › phy213376-sup-0003-DataS1/SimDataFig_Manuscript_Longitudinal_RNA_Z/SimDataFig_Manuscript_Longitudinal_RNA_Z/1605_Paalvast_Lexogen_QCReport.pdf]
